# Supplementary material for: Circumventing bottlenecks in H2O2 photosynthesis over carbon nitride with iodine redox chemistry and electric field effects
Source: Nat Commun. 2024 Jun 3;15:4718. doi: 10.1038/s41467-024-49046-x (PMC11535034; doi:10.1038/s41467-024-49046-x)
Supplement: Supplementary file 1 — Supplementary Information [file 41467_2024_49046_MOESM1_ESM.pdf]

Supplementary Information for

Circumventing bottlenecks in H_2O_2 photosynthesis over carbon nitride with iodine redox chemistry and electric field effects

Chang-Wei Bai[1, #], Lian-Lian Liu[2, #], Jie-Jie Chen[2], Fei Chen[1, **], Zhi-Quan Zhang[1], Yi-Jiao Sun[1], Xin-Jia Chen[1], Qi Yang[3], Han-Qing Yu[2, **]

[1]Key Laboratory of the Three Gorges Reservoir Region's Eco-Environment, Ministry of Education, College of Environment and Ecology, Chongqing University, Chongqing 400045, China

[2]CAS Key Laboratory of Urban Pollutant Conversion, Department of Environmental Science and Engineering, University of Science and Technology of China, Hefei, 230026, China

[3]Key Laboratory of Environmental Biology and Pollution Control, Ministry of Education, College of Environmental Science and Engineering, Hunan University, Changsha 410082, China

Contents: Pages 91

Supplementary Figures **1-63**

Supplementary Tables **1-14**

Supplementary References

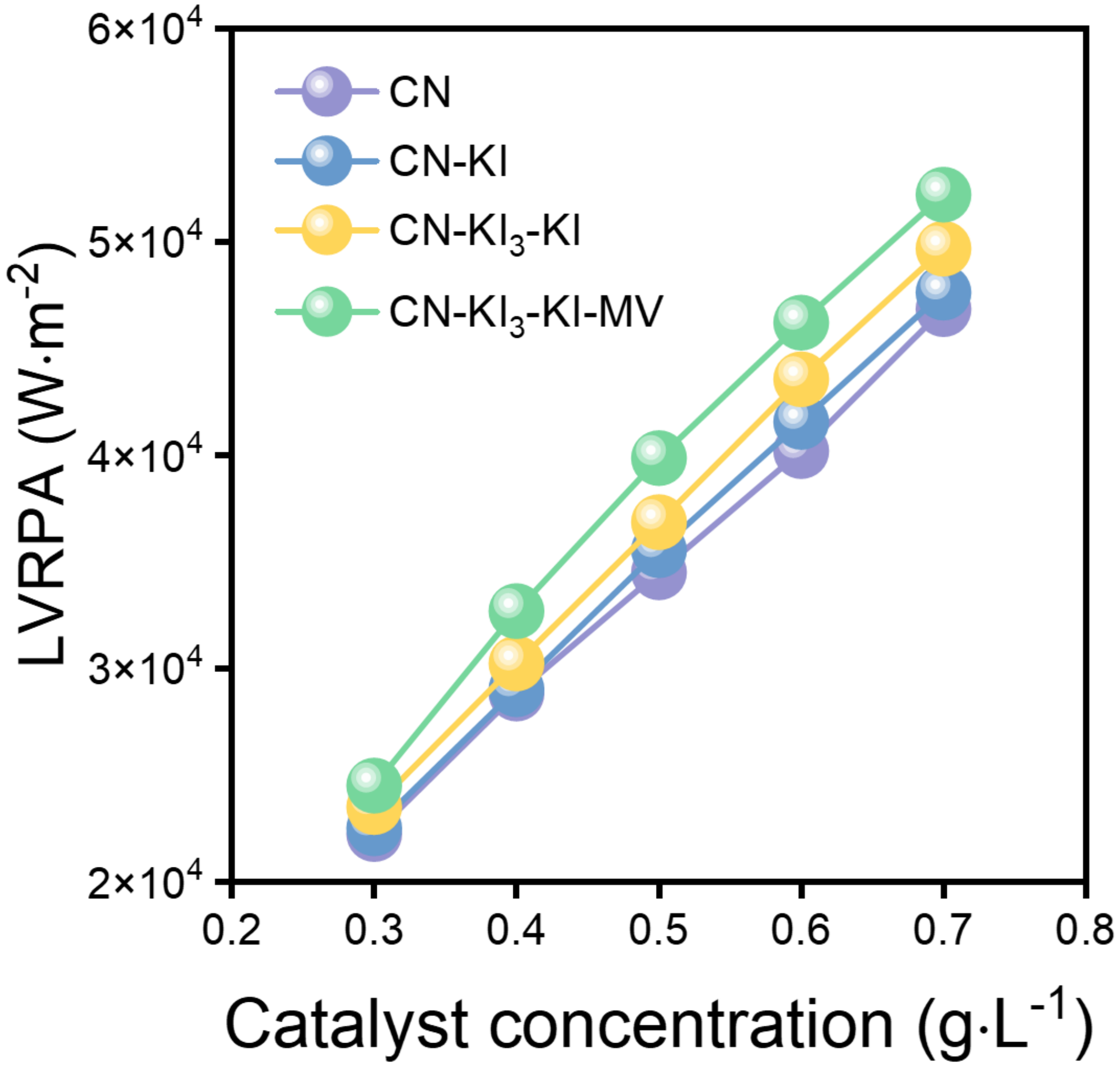

Supplementary Figure 1 | Surface LVRPA at different photocatalyst concentrations.

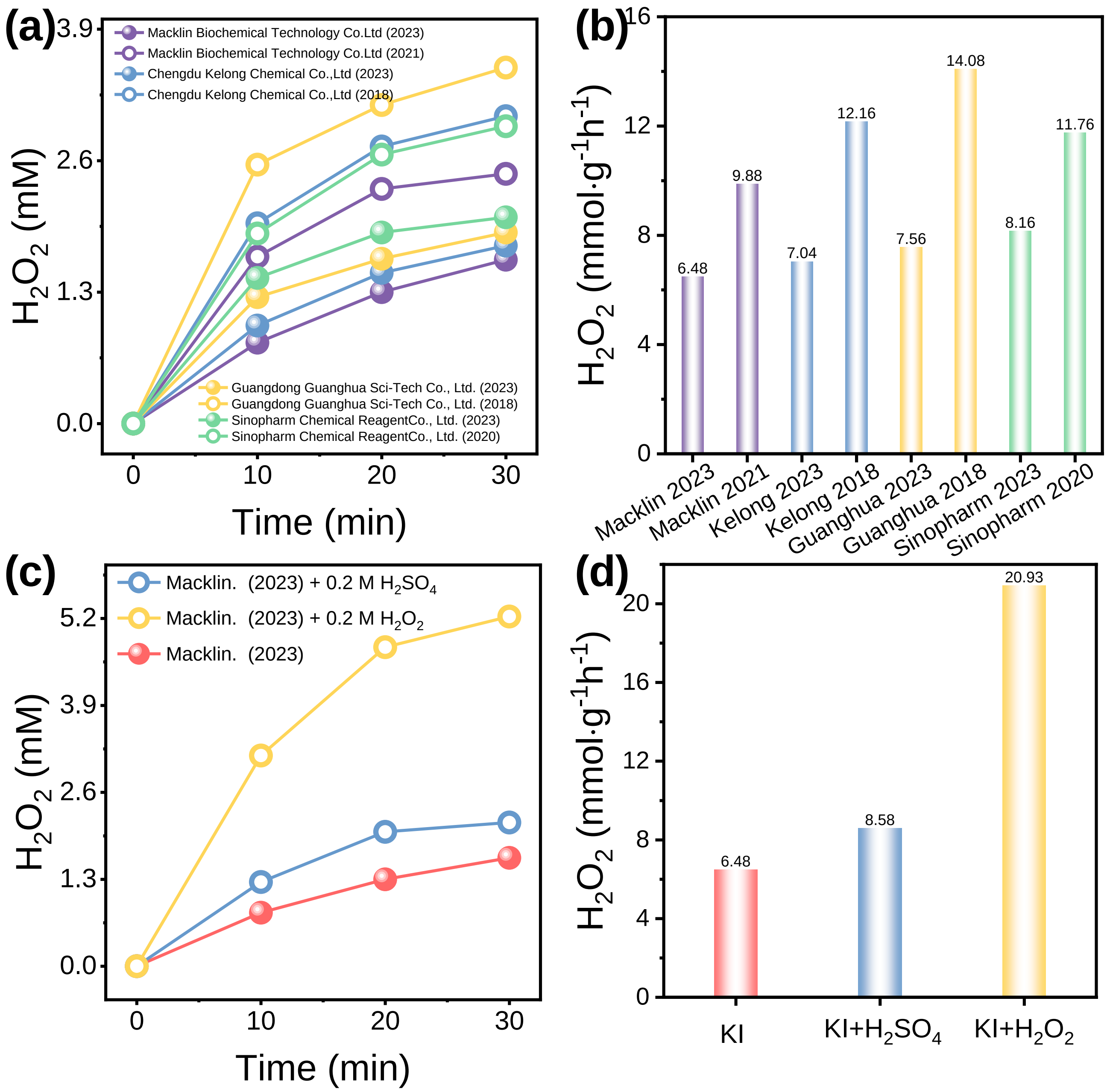

Supplementary Figure 2 | Photocatalytic H_2O_2 production activities (a) and corresponding evolution rates (b) for the samples obtained from different production dates of KI (Outdated KI or oxidized KI). Photocatalytic H_2O_2 production activities (c) and corresponding evolution rate (d) of artificially oxidized KI samples.

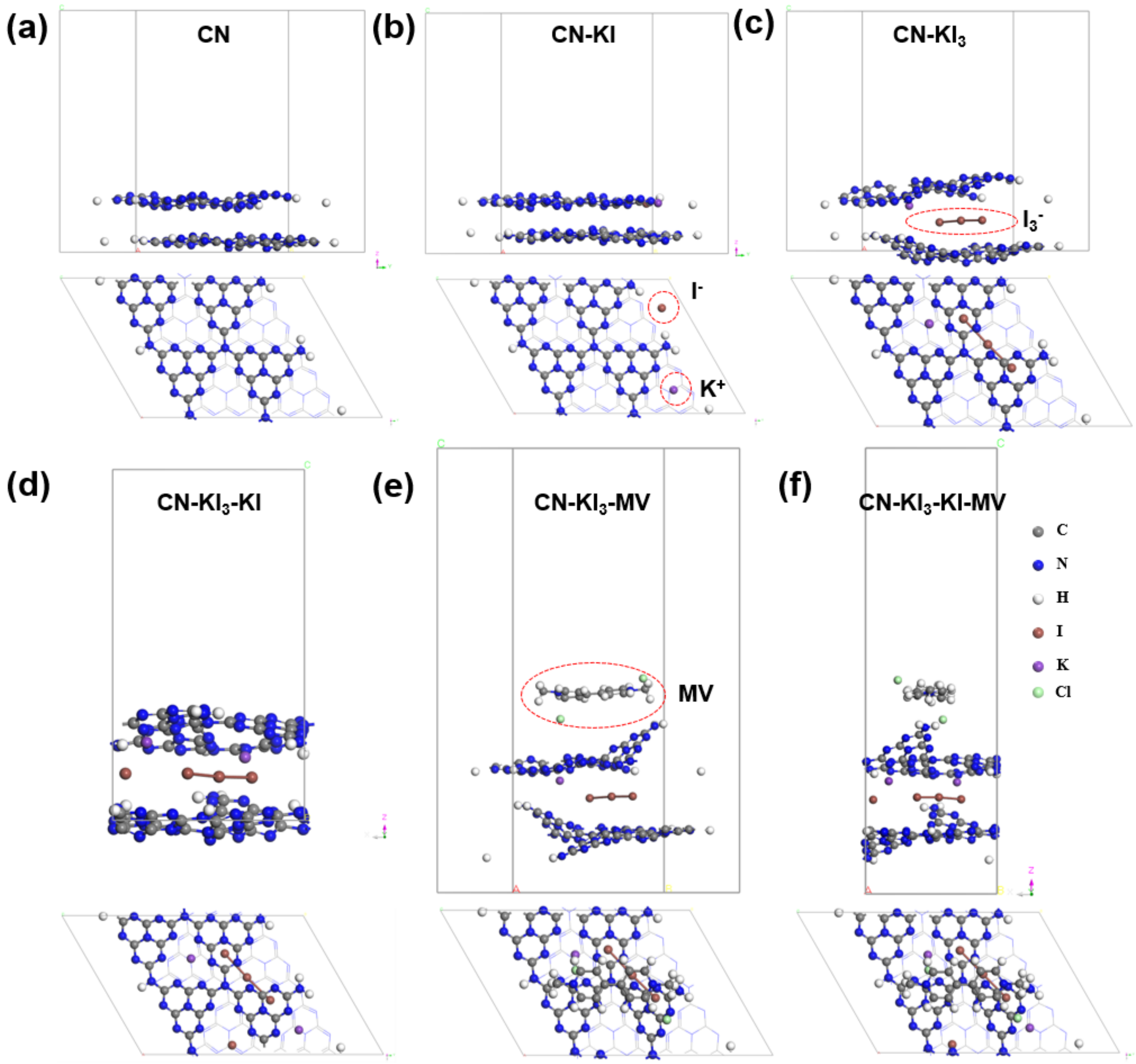

Supplementary Figure 3 | Detailed optimized geometry structures: (a) CN, (b) CN-KI, (c) CN-KI_3, (d) CN-KI_3-KI, (e) CN-KI_3-MV and (f) CN-KI_3-KI-MV.

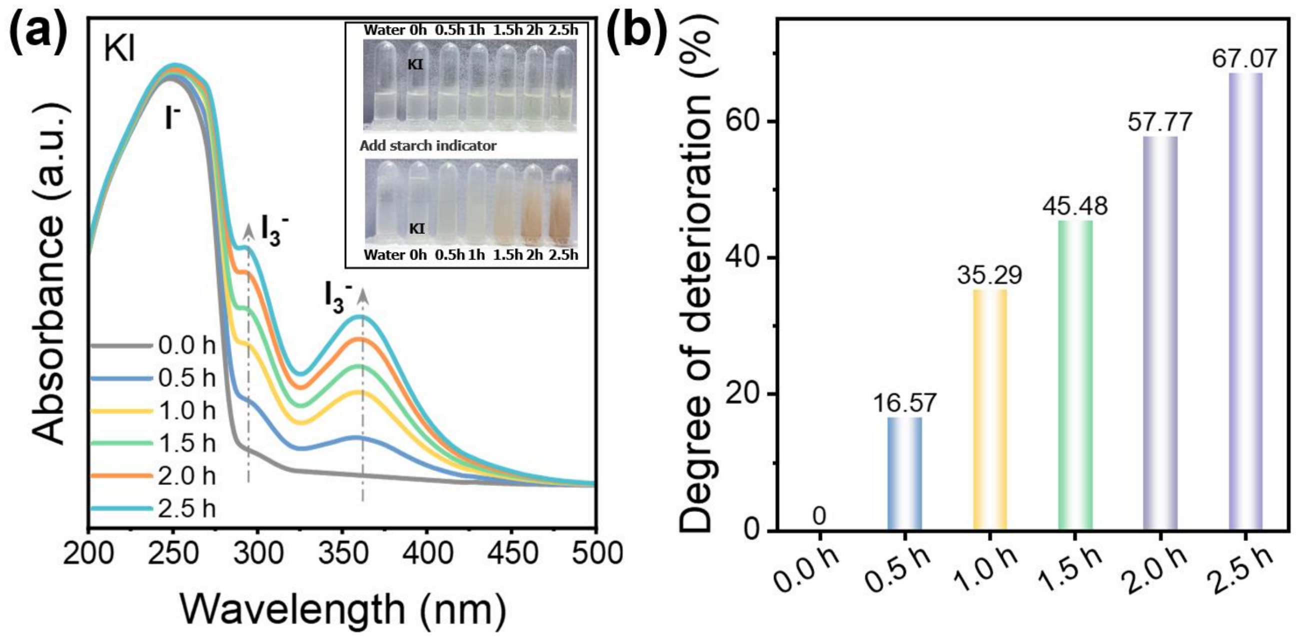

Supplementary Figure 4 | UV/Vis absorption spectra (a) and the corresponding degree of deterioration (b) of KI solutions with different irradiation times. Inset: Color changes in KI solution with starch indicator under different illumination times.

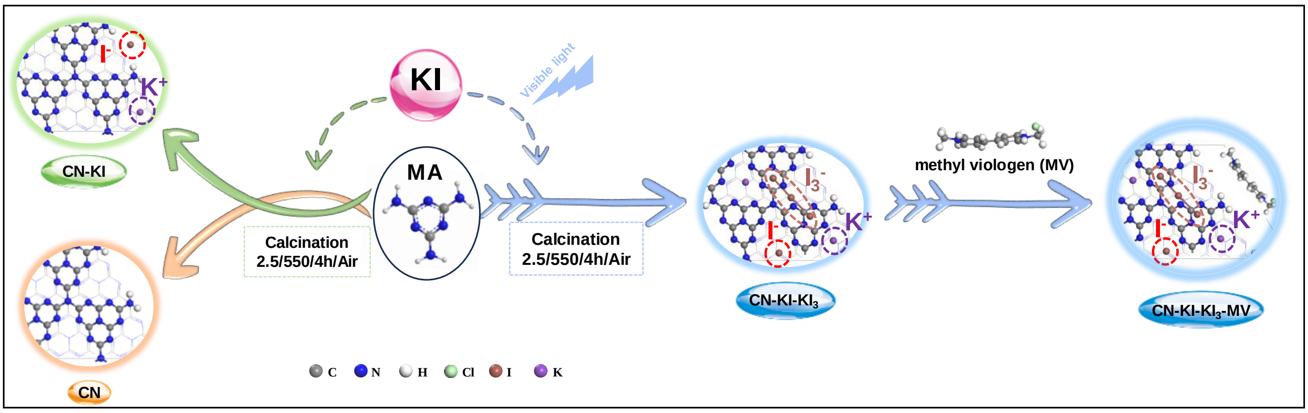

Supplementary Figure 5 | Schematic synthesis of CN, CN-KI, CN-KI_3-KI, and CN-KI_3-KI-MV.

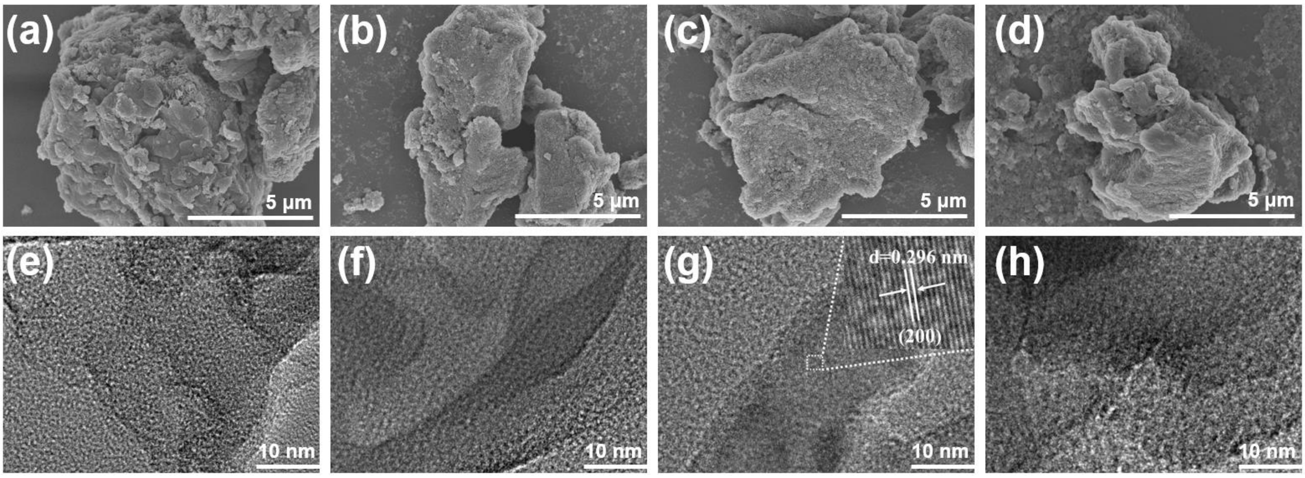

Supplementary Figure 6 | SEM and HRTEM images of the prepared samples. (a, e) CN. (b, f) CN-KI. (c, g) CN-KI_3-KI, Inset: Magnified and clearly distinguishable lattice spacing. (d, h) CN-KI_3-KI-MV.

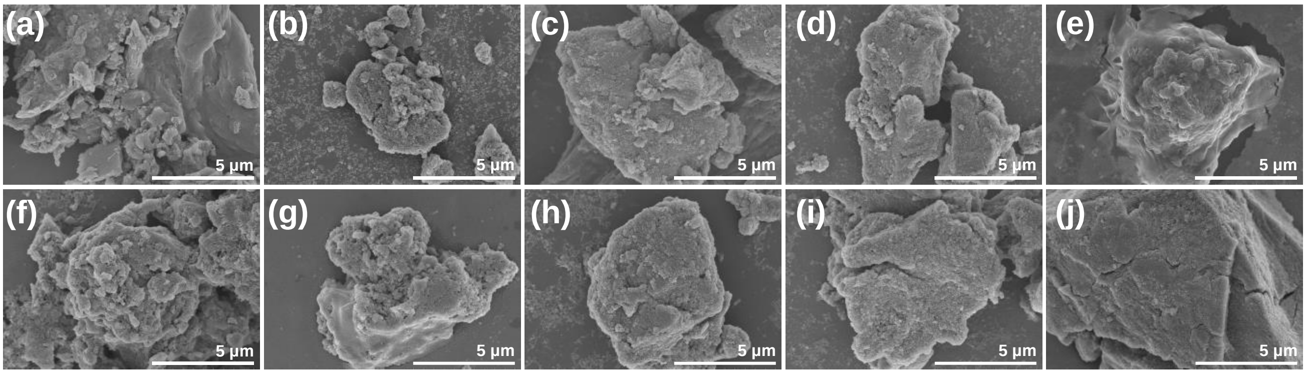

Supplementary Figure 7 | SEM images of the samples with different MA-KI ratios and different MA-KI ratios after photocatalytic oxidation. (a, f) 2: 1. (b, g) 2: 2. (c, h) 2: 4. (d, i) 2: 6. (e, j) 2: 8.

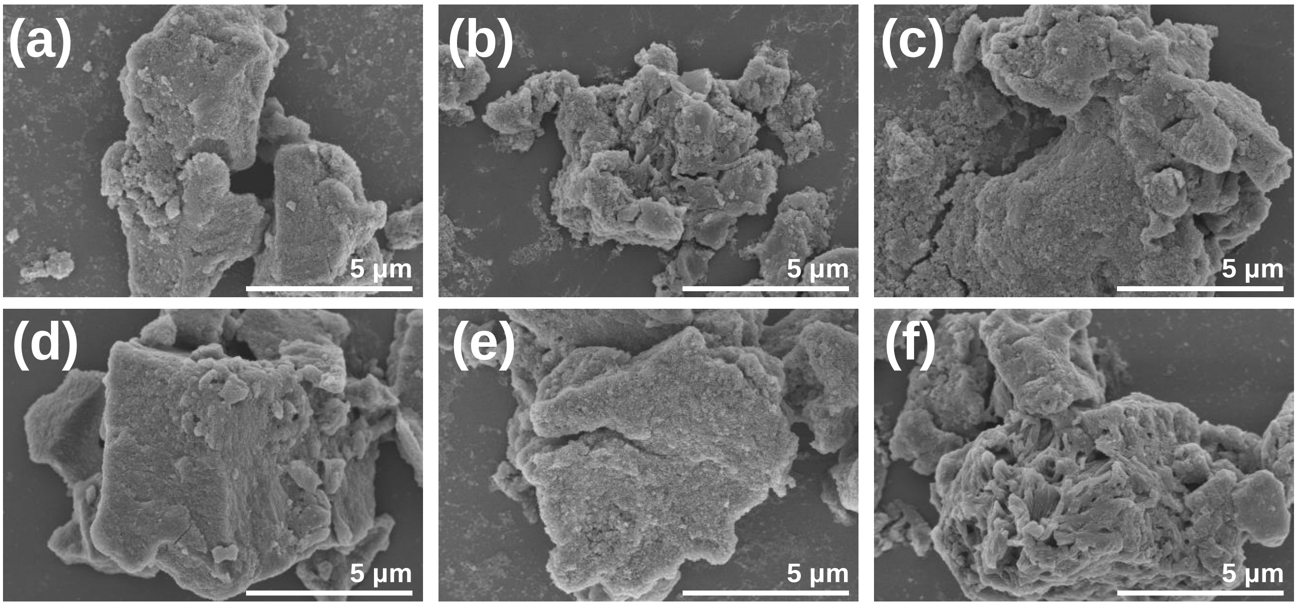

Supplementary Figure 8 | SEM images of the samples with different photocatalytic oxidation durations (2: 6): (a) 0 h. (b) 0.5 h. (c) 1 h. (d) 1.5 h. (e) 2 h. (f) 2.5 h.

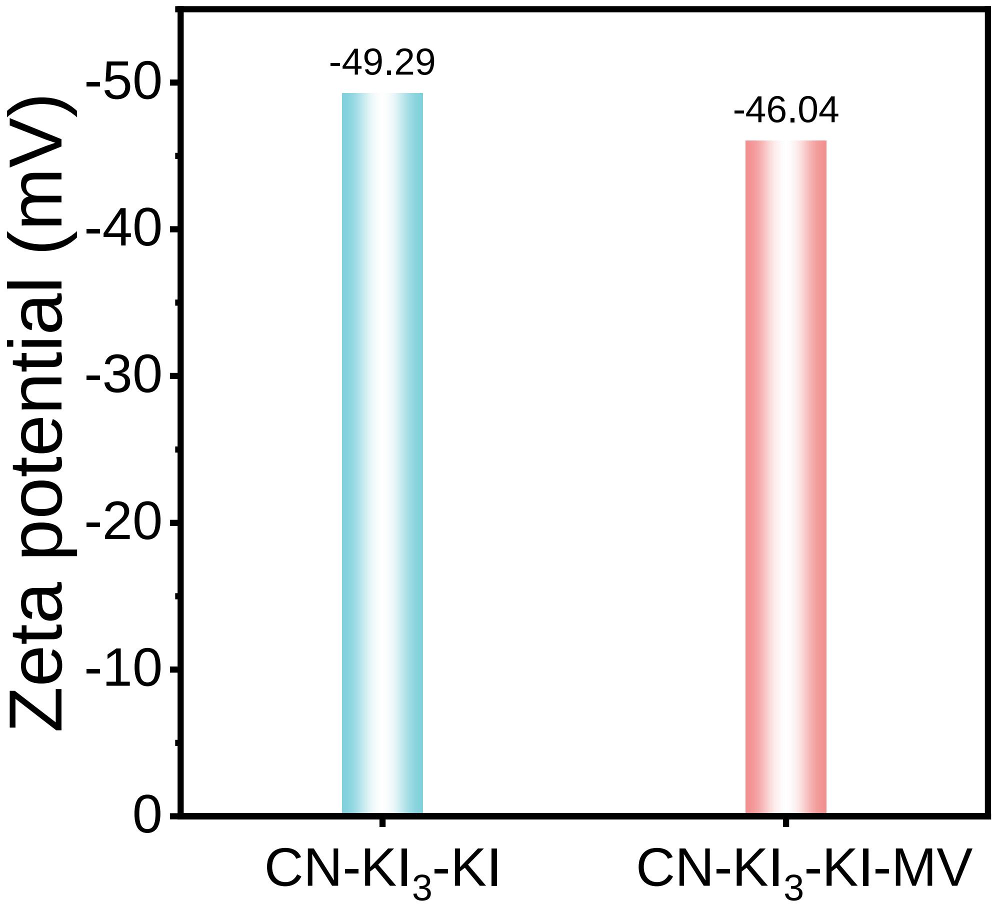

Supplementary Figure 9 | Zeta potentials of CN-KI_3-KI and CN-KI_3-KI-MV.

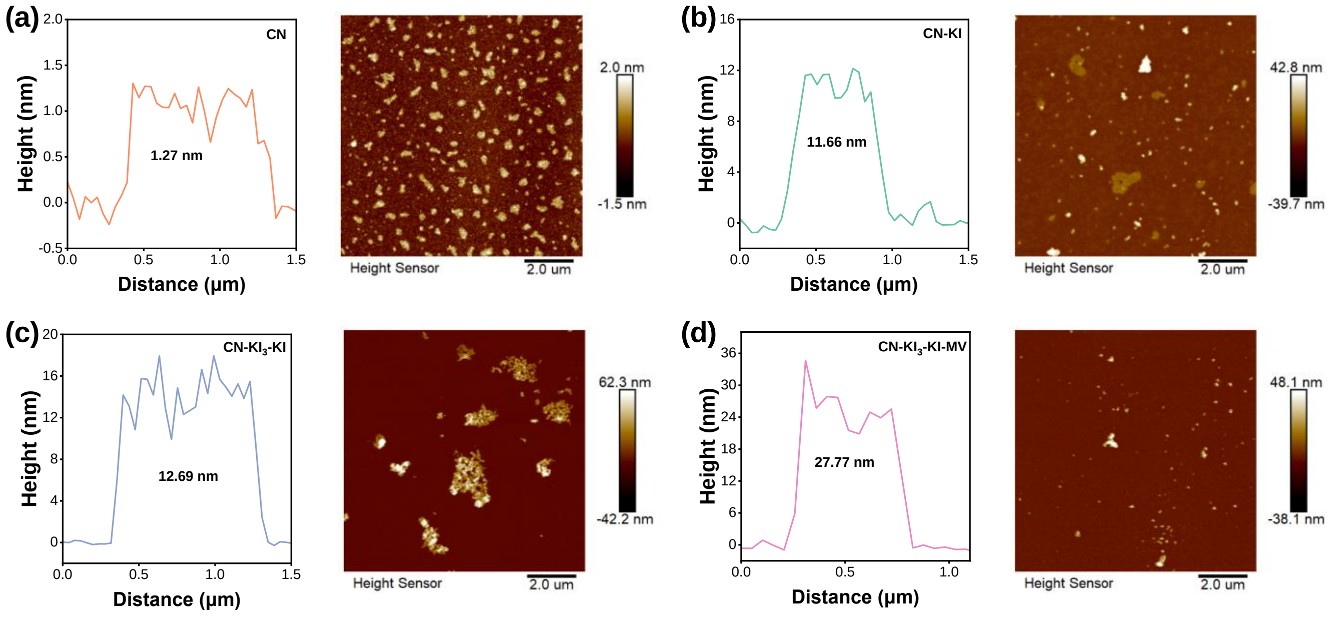

Supplementary Figure 10 | AFM images and the height curves of (a) CN, (b) CN-KI, (c) CN-KI_3-KI, and (d) CN-KI_3-KI-MV.

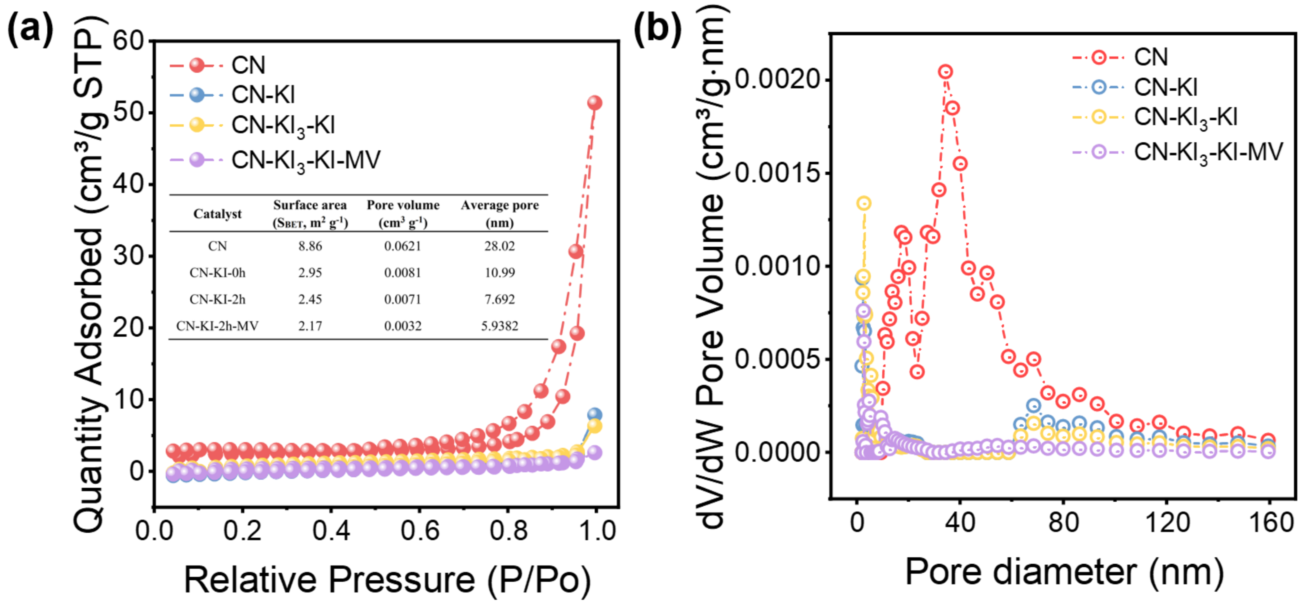

Catalyst	Surface area (S_{BET}, $m^2\ g^{-1}$)	Pore volume ($cm^3\ g^{-1}$)	Average pore (nm)
CN	8.86	0.0621	28.02
CN-KI-0h	2.95	0.0081	10.99
CN-KI-2h	2.45	0.0071	7.692
CN-KI-2h-MV	2.17	0.0032	5.9382

Supplementary Figure 11 | Surface area analysis. (a) N_2 adsorption-desorption isotherms, Inset: Detailed data, and (b) pore size distribution of CN, CN-KI, CN-KI_3-KI, and CN-KI_3-KI-MV.

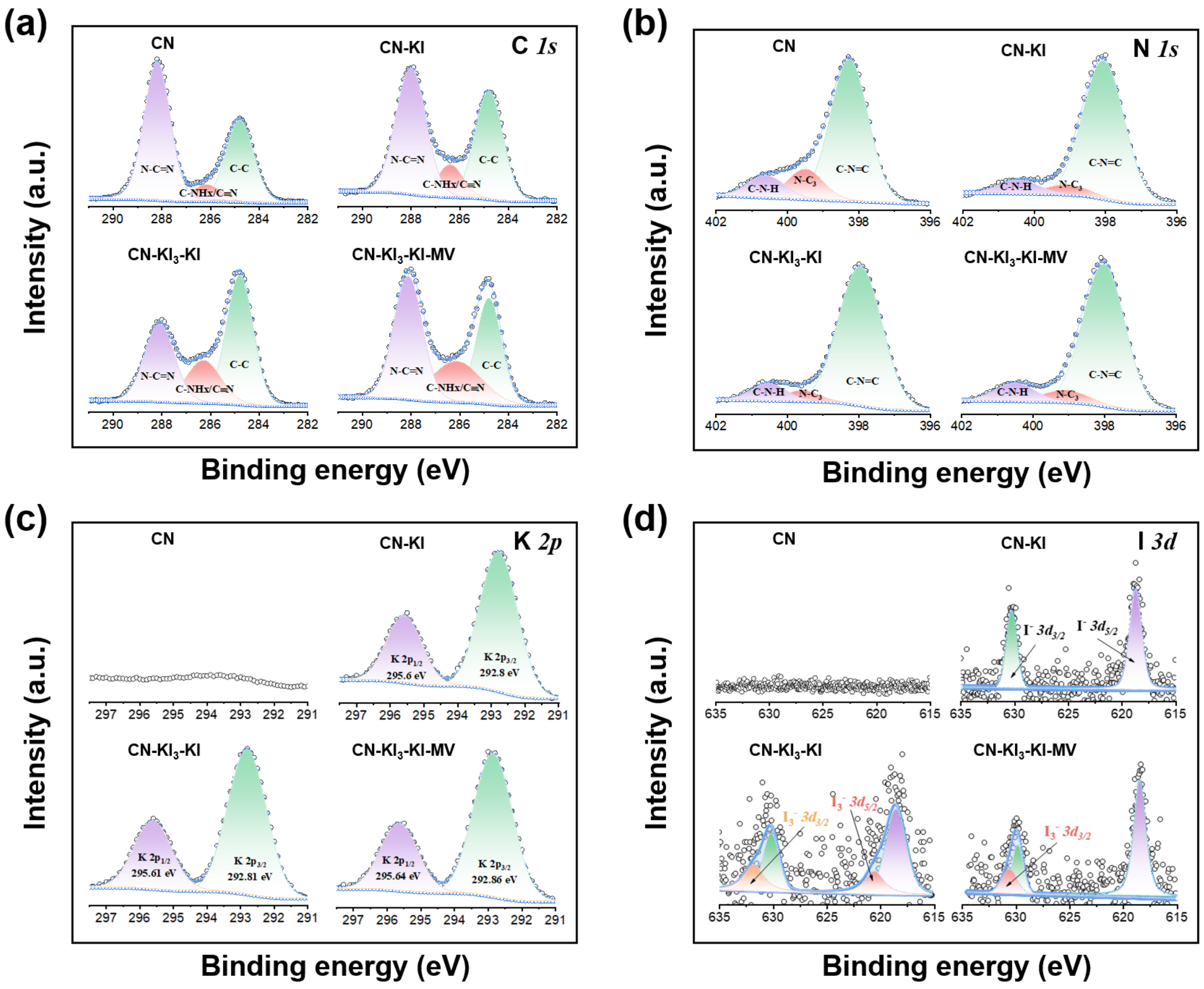

Supplementary Figure 12 | High-resolution XPS spectra of different catalysts. (a) C *1s*, (b) N *1s*, (c) K *2p*, and (d) I *3d* XPS spectra of CN, CN-KI, CN-KI_3-KI, and CN-KI_3-KI-MV.

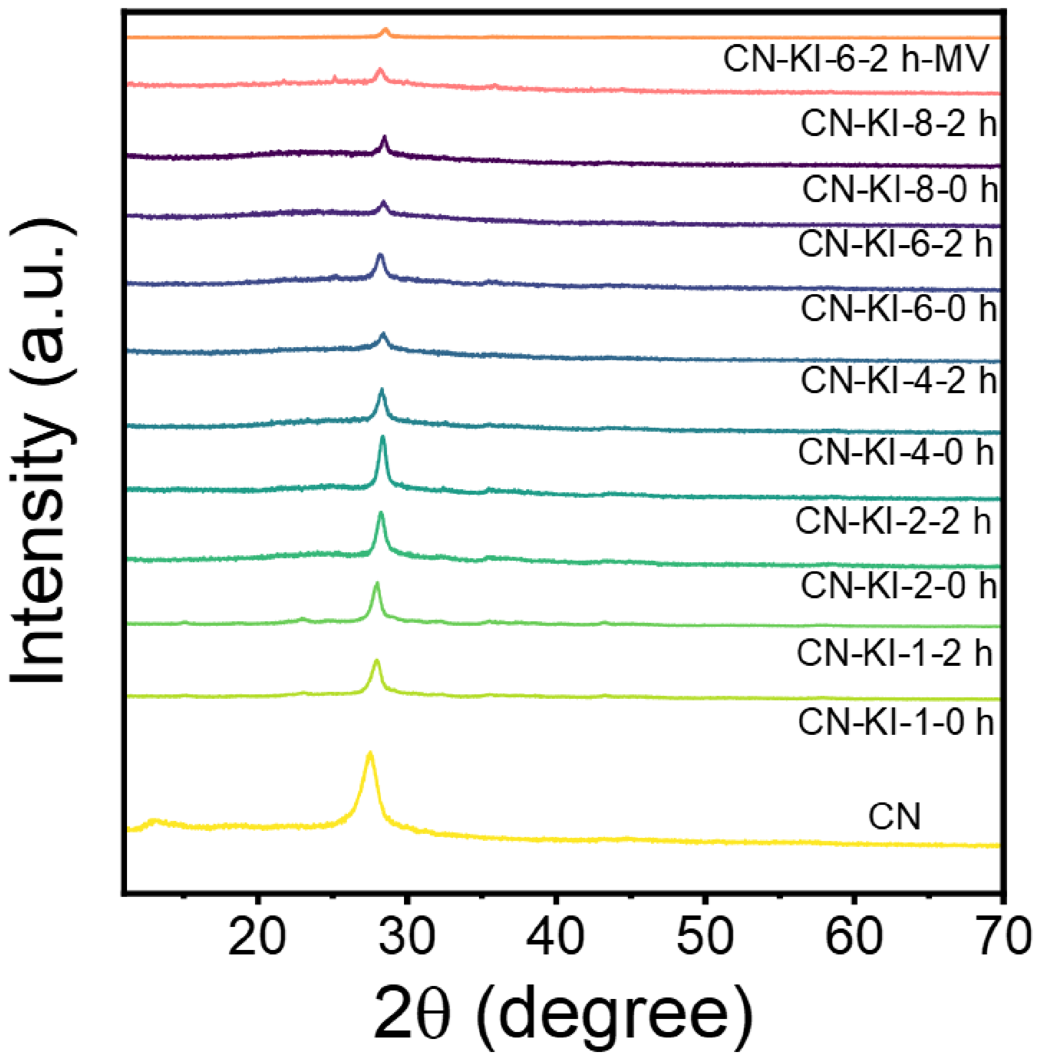

Supplementary Figure 13 | XRD spectra of the prepared samples.

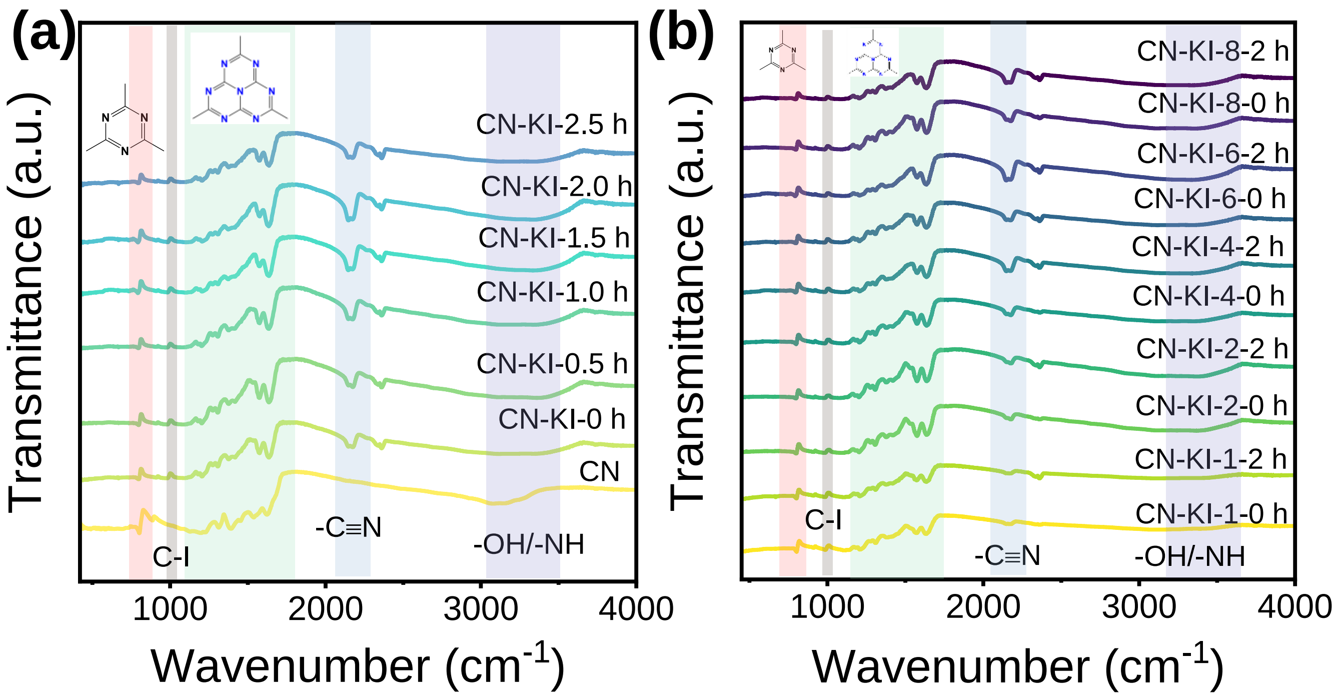

Supplementary Figure 14 | FTIR spectra of the as-prepared samples with (a) different photocatalytic oxidation durations and (b) different MA-KI ratios.

The structural changes of the different photocatalysts were characterized by the Fourier-transform infrared (FTIR) analysis (Fig. **1k** and Supplementary Figure **14**). All samples exhibited peaks at 820, 1100-1700, and 3000-3500 cm^{-1}, attributable to the stretching vibrations of the triazine ring unit, C=N heterocycles, and N-H groups, respectively. However, a new vibration peak at 2166 cm^{-1} was observed in all the samples except CN, due to the cyano group generated by the deprotonation of $-C-NH_2$. Moreover, a distinctive vibration peak around 1000 cm^{-1} was consistently observed in all samples except CN, which was attributed to the presence of the C-I bond. This observation serves as pivotal evidence for the integration of iodine into the CN structure.

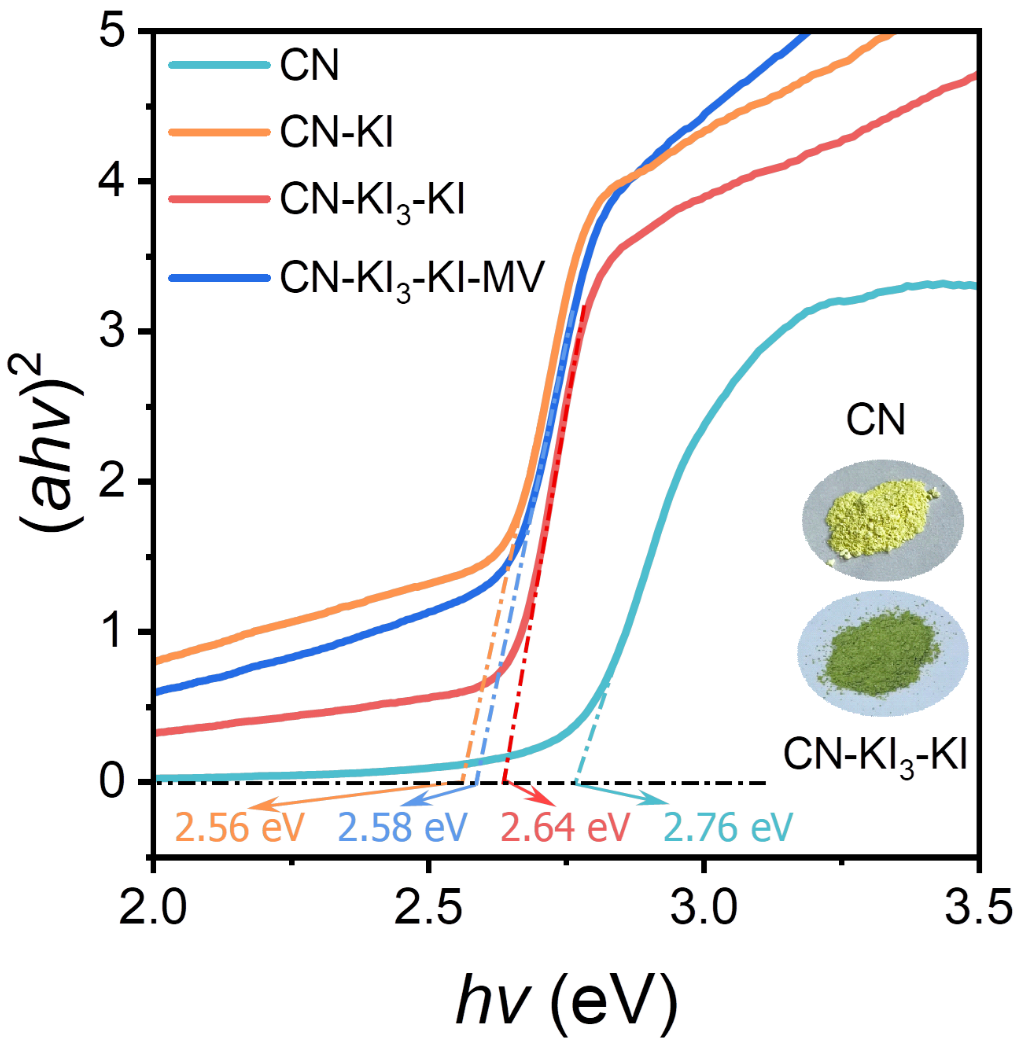

Supplementary Figure 15 | Energy band diagrams of CN, CN-KI, CN-KI_3-KI, and CN-KI_3-KI-MV.

Inset: Photographs of CN and CN-KI_3-KI.

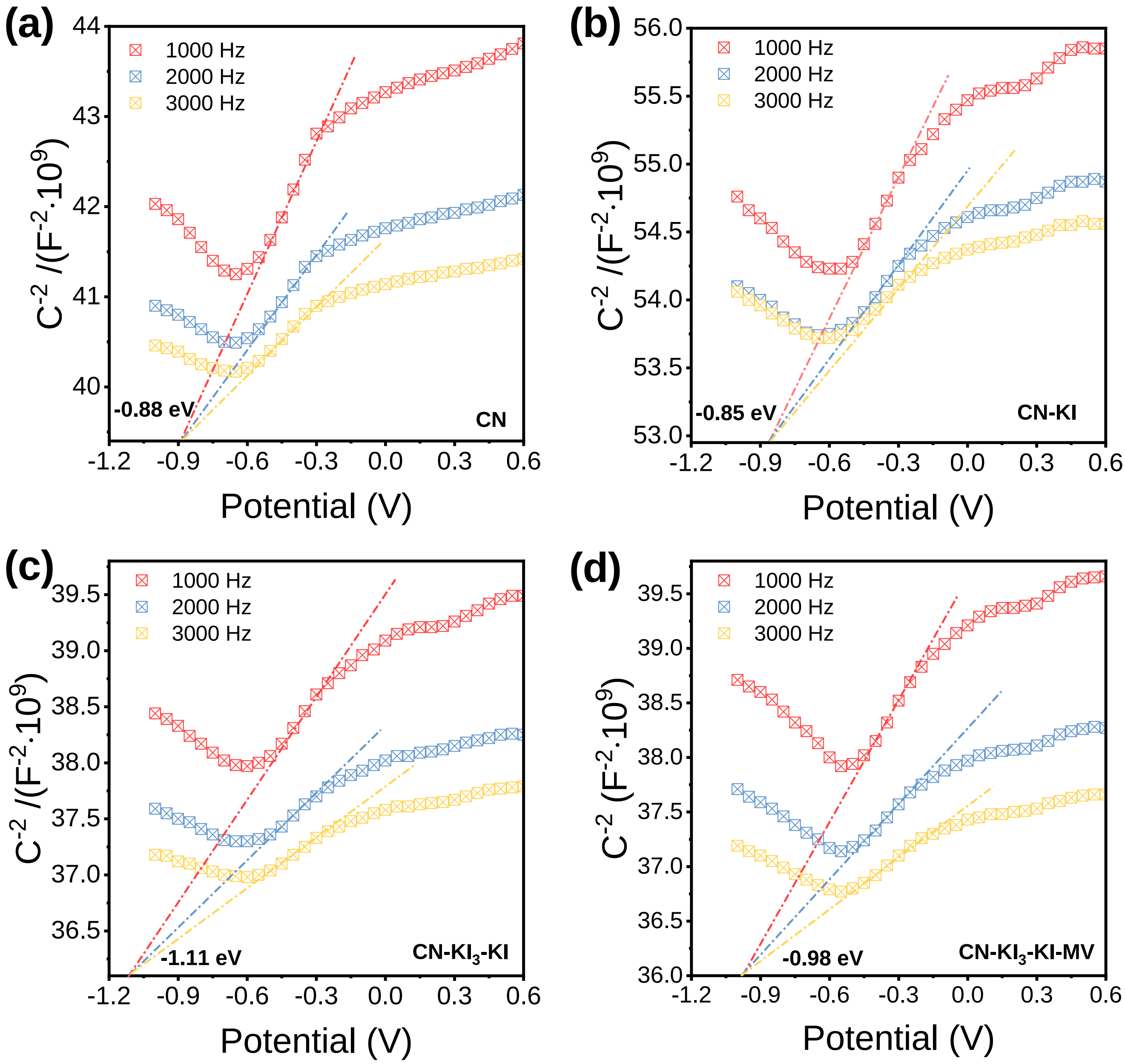

Supplementary Figure 16 | Mott-Schottky plots of the prepared samples. (a) CN. (b) CN-KI. (c) CN-KI_3-KI. (d) CN-KI_3-KI-MV.

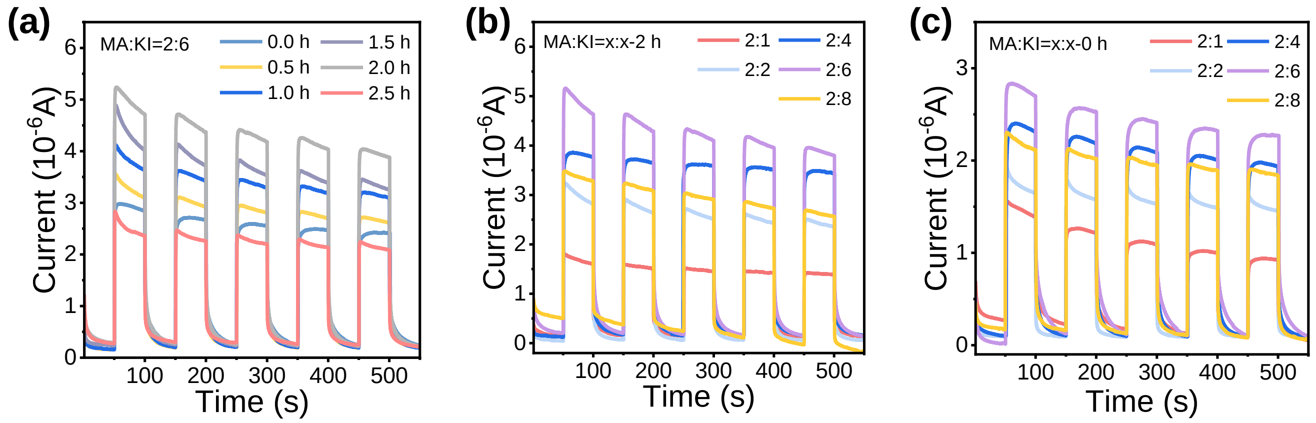

Supplementary Figure 17 | Photocurrent intensities of the prepared samples: (a) different photocatalytic oxidation durations and (b, c) with different MA-KI ratios.

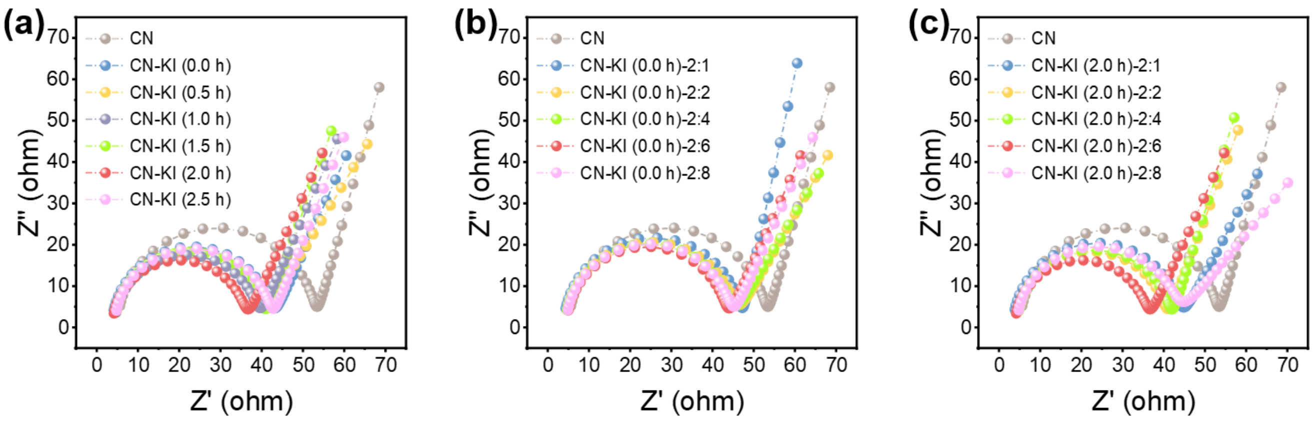

Supplementary Figure 18 | Electrochemical impedance spectra of the as-prepared samples: (a) different photocatalytic oxidation durations and (b, c) with different MA-KI ratios.

Electrochemical impedance spectra (EIS) were used to assess electron migration resistance. With increasing photocatalytic oxidation durations, the arc radius in EIS gradually decreased, indicating that the injection of I_3^- facilitated the transfer of charges in the catalyst. Among them, CN-KI_3-KI displayed the smallest arc radius (Fig. **3c** and Supplementary Figure **18)**, indicating that CN-KI_3-KI had the fastest electron transfer kinetics and surface reaction rates.

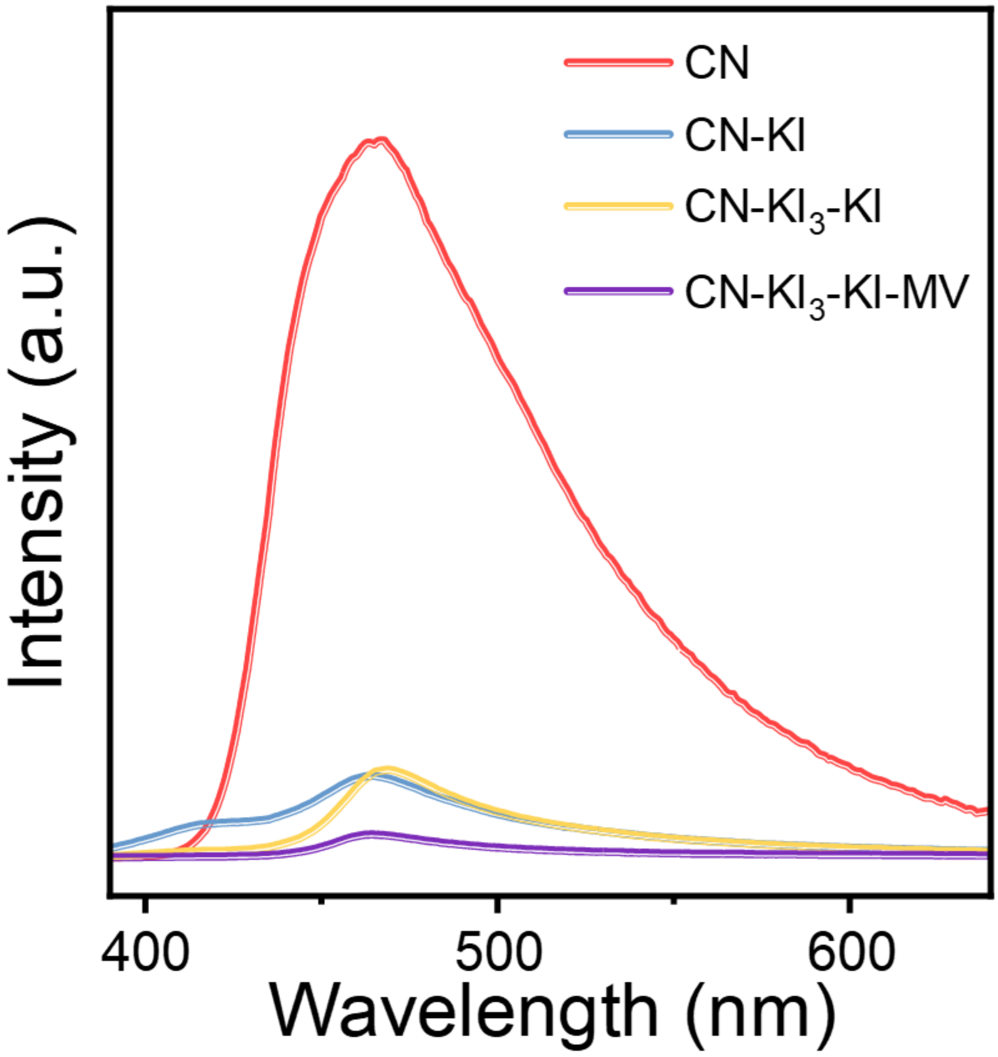

Supplementary Figure 19 | Steady-state photoluminescence (PL) spectra of CN, CN-KI, CN-KI_3-KI, and CN-KI_3-KI-MV.

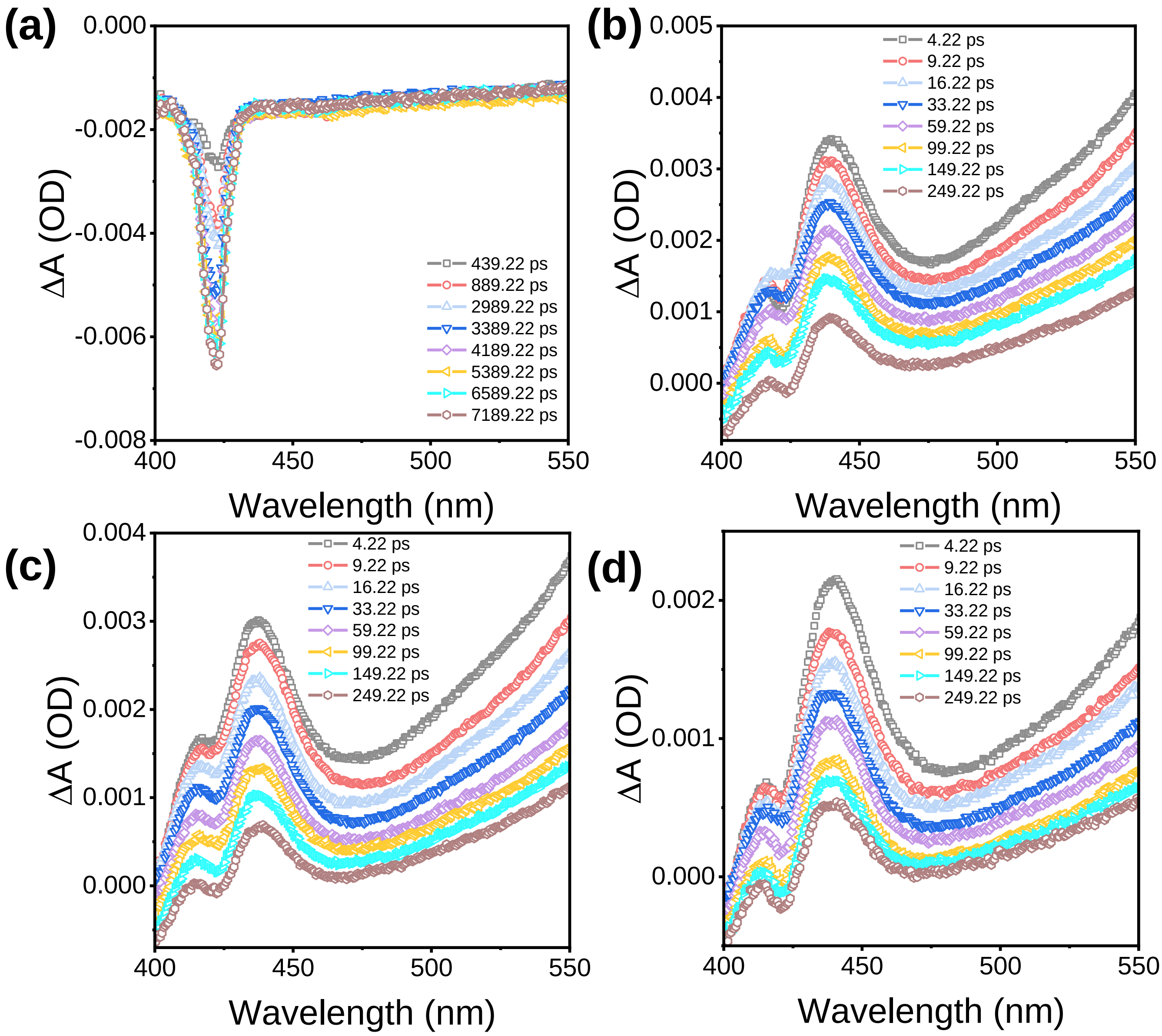

Supplementary Figure 20 | Transient absorption spectra of (a) CN, (b) CN-KI, (c) CN-KI_3-KI, and (d) CN-KI_3-KI-MV recorded by 400 nm femtosecond laser pulse excitation in air.

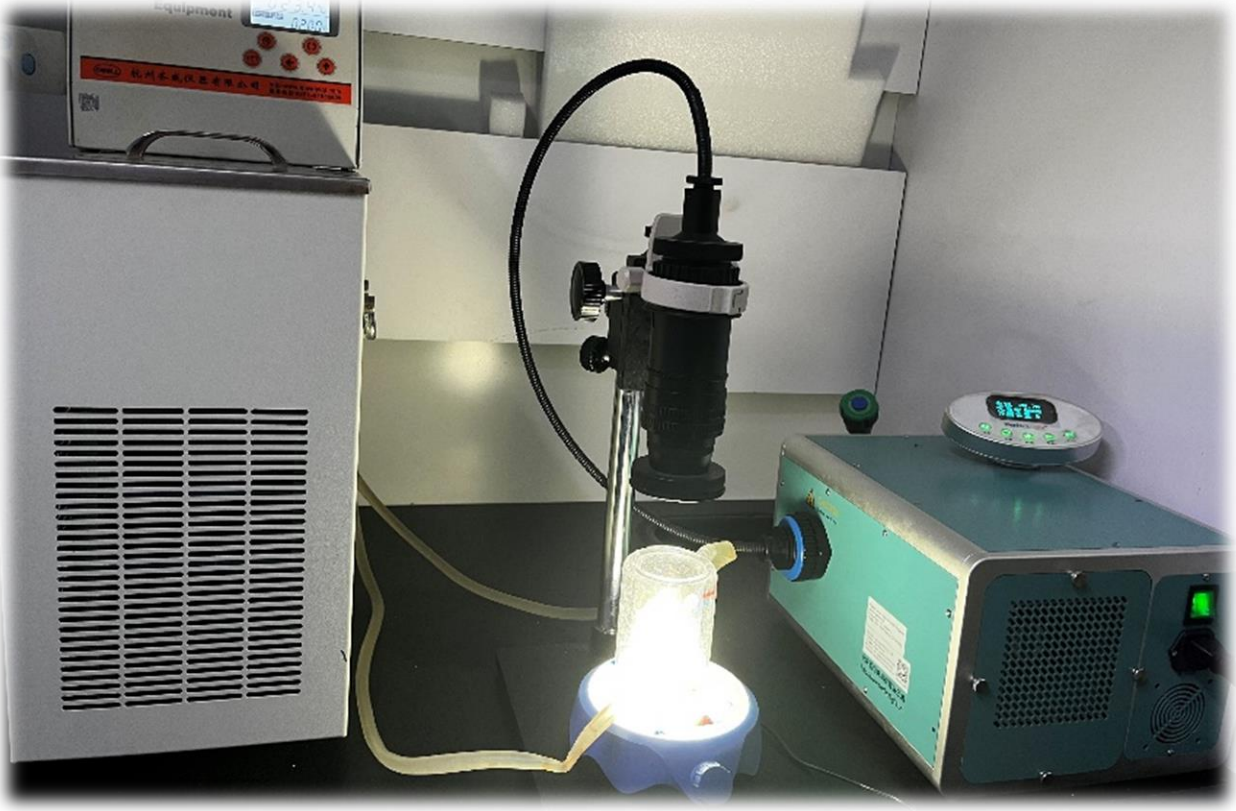

Supplementary Figure 21 | Diagram of the reaction apparatus for photocatalytic H_2O_2 production.

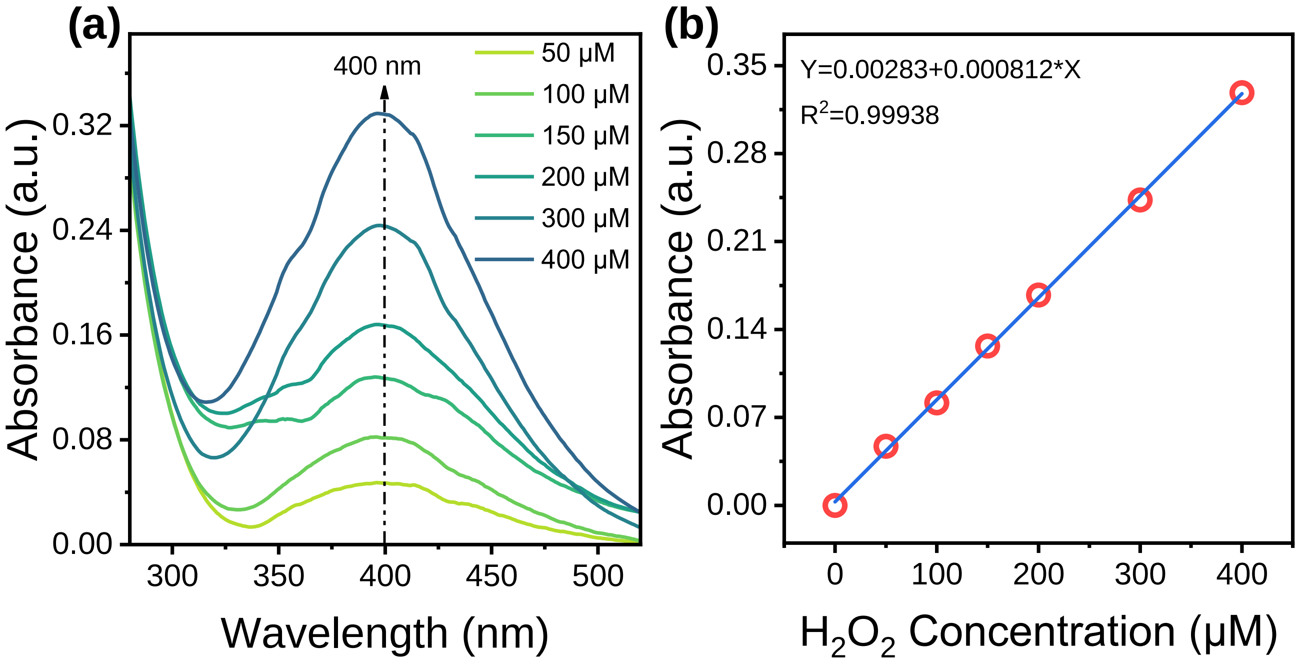

Supplementary Figure 22 | Standard curve for detecting H_2O_2. (a) Determination of UV-vis absorption intensity of different H_2O_2 concentrations by potassium titanium oxalate method. (b) The linear fitting formula of standard H_2O_2 concentration.

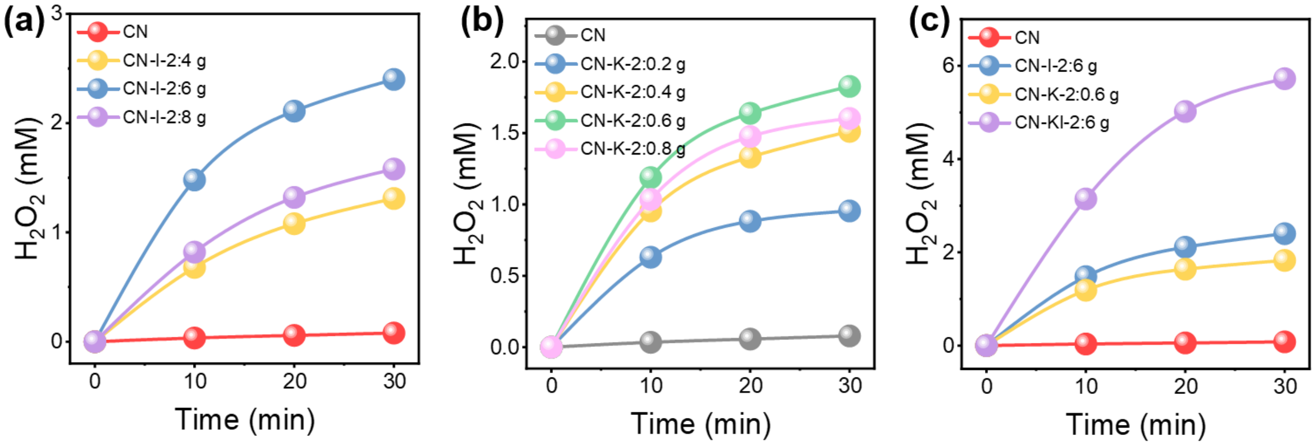

Supplementary Figure 23 | Photocatalytic H_2O_2 production of (a) singly iodine-doped samples, (b) singly potassium-doped samples, and (c) iodine-potassium co-doped samples.

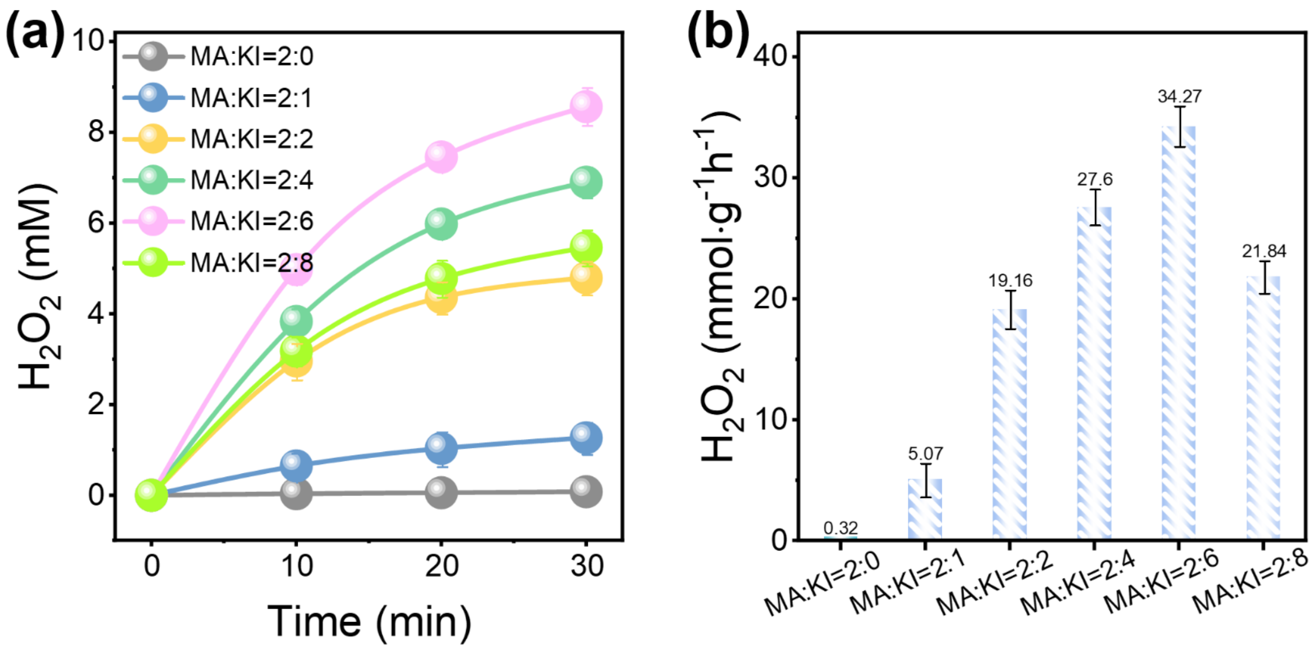

Supplementary Figure 24 | Photocatalytic H_2O_2 production (a) and corresponding evolution rates (b) for samples with different MA-KI ratios. The error bars represent the standard deviation of three replicate tests.

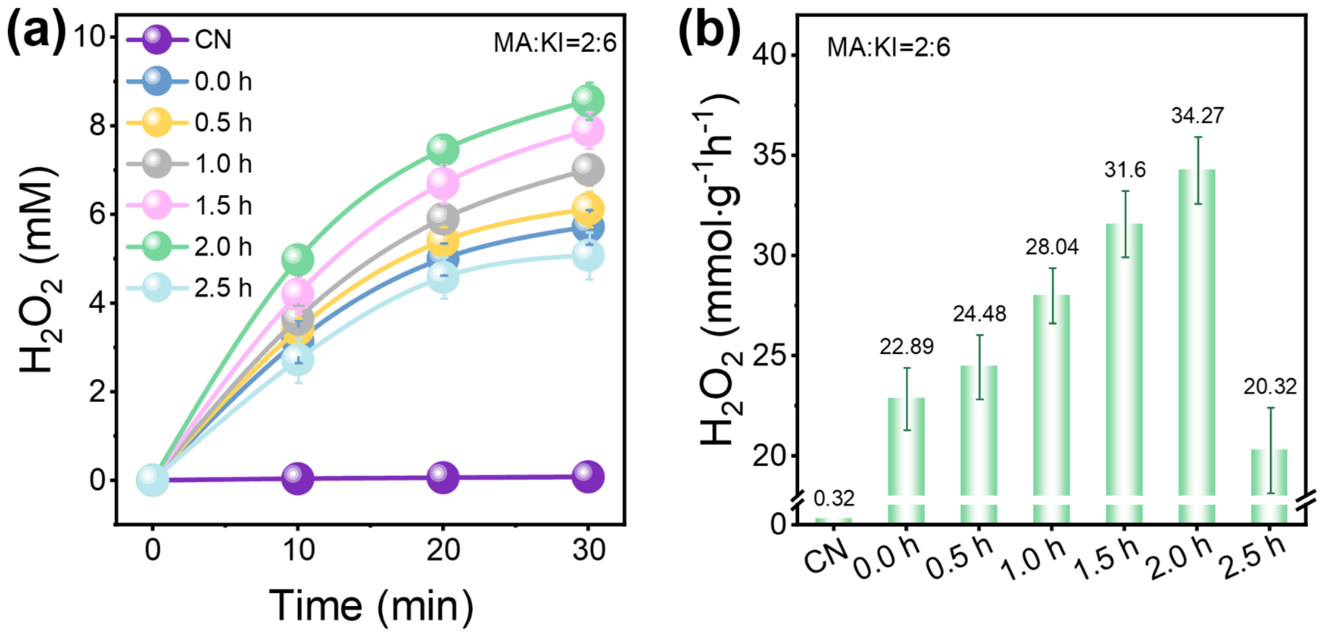

Supplementary Figure 25 | Photocatalytic H_2O_2 production activities (a) and corresponding evolution rates (b) for the samples with different photocatalytic oxidation durations. The error bars represent the standard deviation of three replicate tests.

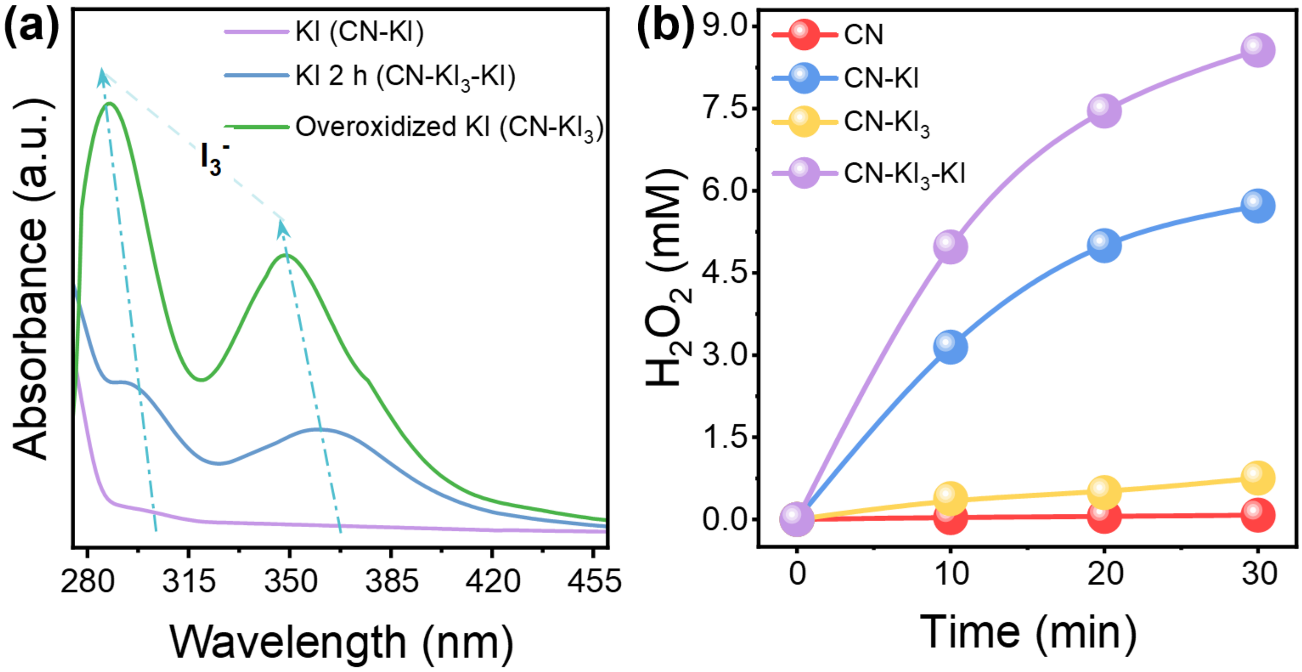

Supplementary Figure 26 | UV-visible absorption spectra of different potassium iodide solutions and their corresponding H_2O_2 production activity. (a) UV-vis absorption spectra of KI solutions at different irradiation times and (b) the H_2O_2 production activities of CN modified with these different KI.

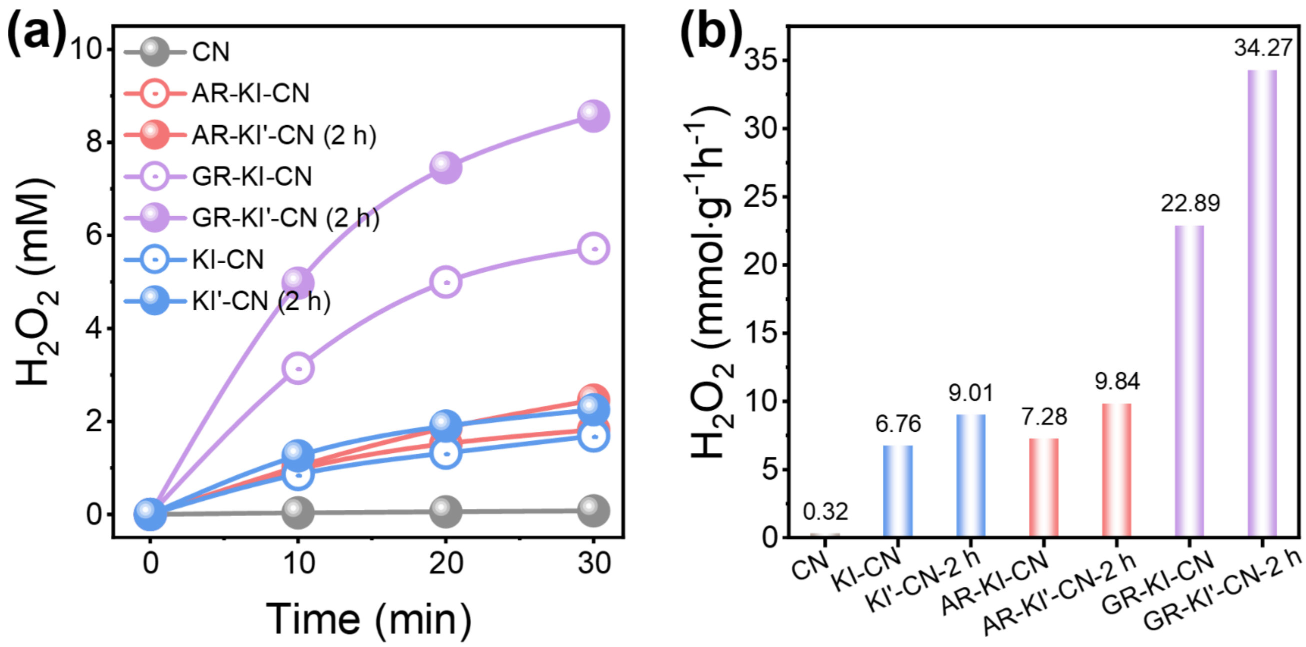

Supplementary Figure 27 | Photocatalytic H_2O_2 production activities (a) and corresponding evolution rates (b) of different CN precursors modified samples.

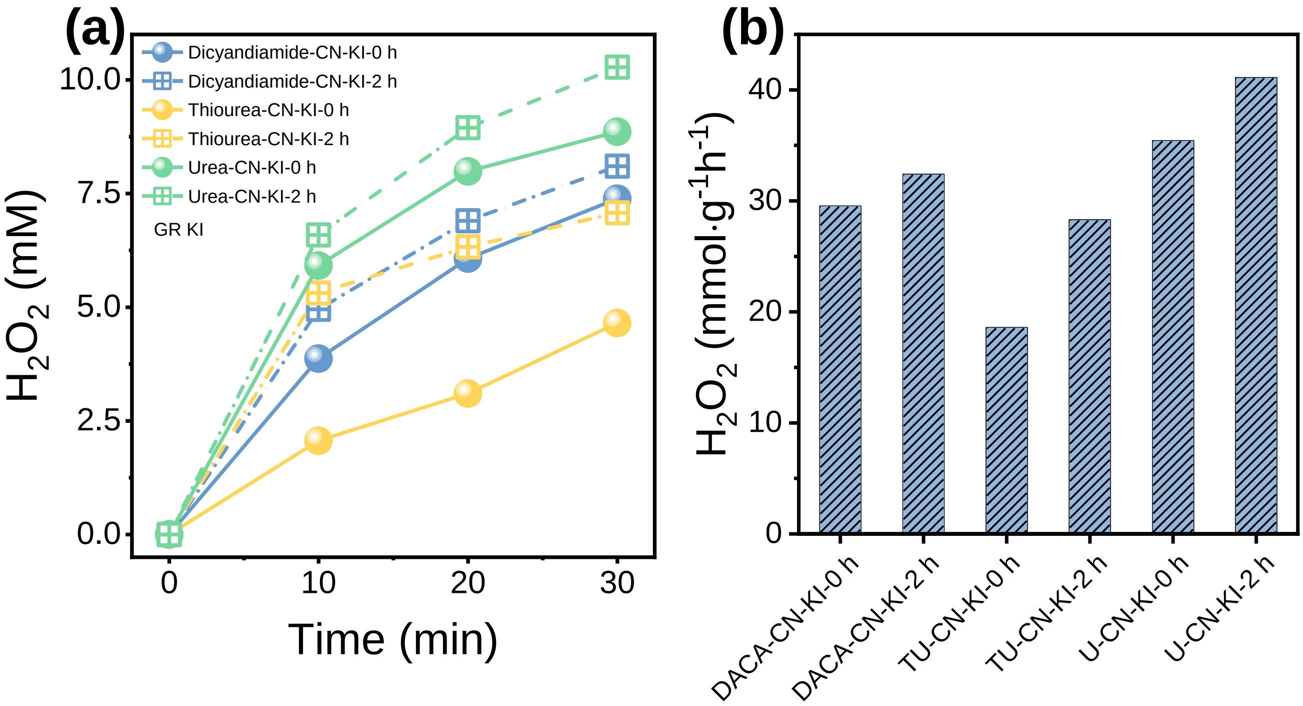

Supplementary Figure 28 | Photocatalytic H_2O_2 production activities (a) and corresponding evolution rates (b) for the samples obtained from different CN precursors.

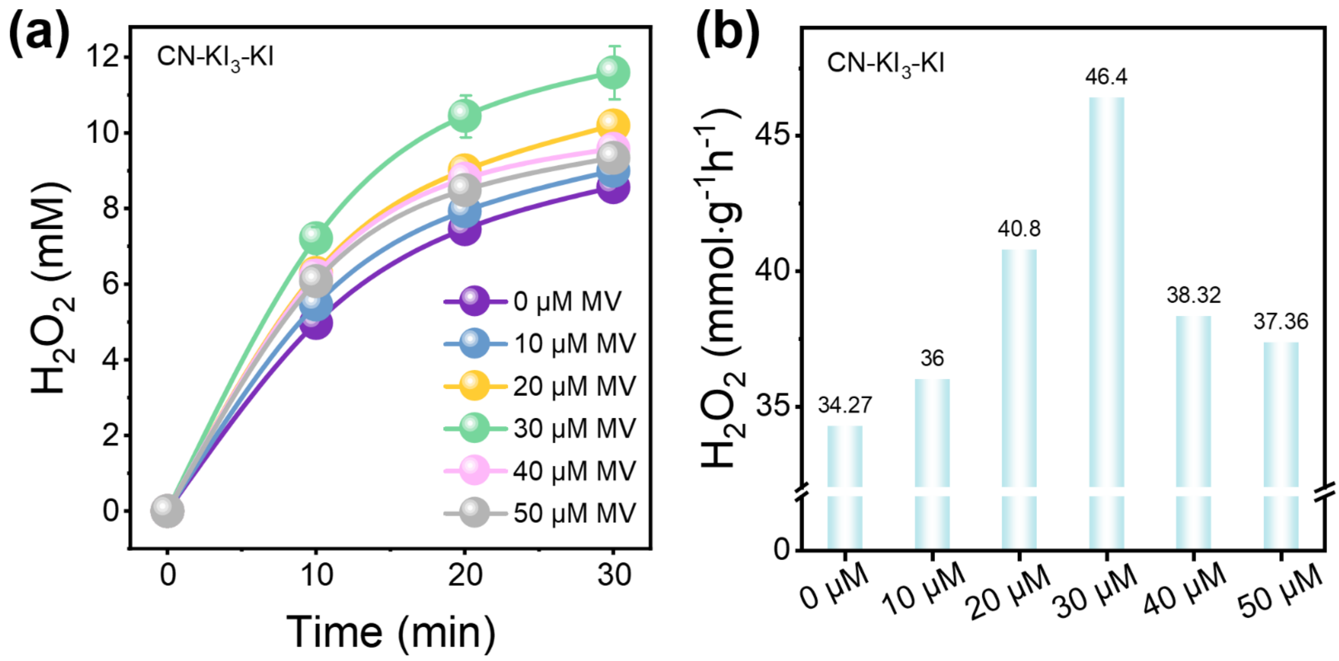

Supplementary Figure 29 | Photocatalytic H_2O_2 production activities (a) and corresponding evolution rates (b) for samples with different MV loading amounts. The error bars represent the standard deviation of three replicate tests.

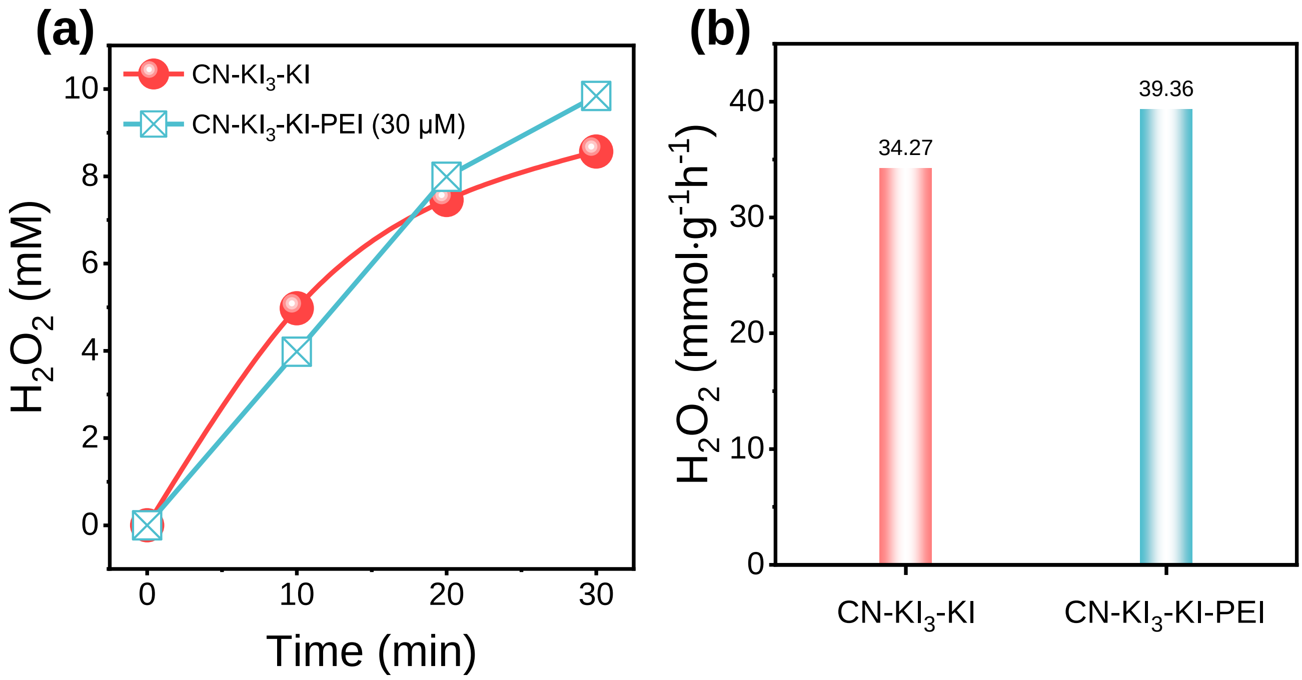

Supplementary Figure 30 | Photocatalytic H_2O_2 production activities (a) and corresponding evolution rates (b) of PEI-modified sample.

After incorporating polyethyleneimine (PEI) as a modifier into the CN-KI_3-KI system, our study demonstrated a notable enhancement in photocatalytic activity. This finding corroborates the scalable applicability of our designed catalyst, which integrated externally attached small organic molecules. The objective of this integration was to establish an external electric field, a strategic modification aimed at augmenting photocatalytic efficiency.

The successful application of PEI in this context not only validates the effectiveness of our catalyst design but also exemplifies the broader potential of employing small organic molecules to manipulate external electric fields in photocatalytic systems. This approach represents a significant advancement in the field, offering a versatile methodology for enhancing photocatalytic performance in various applications.

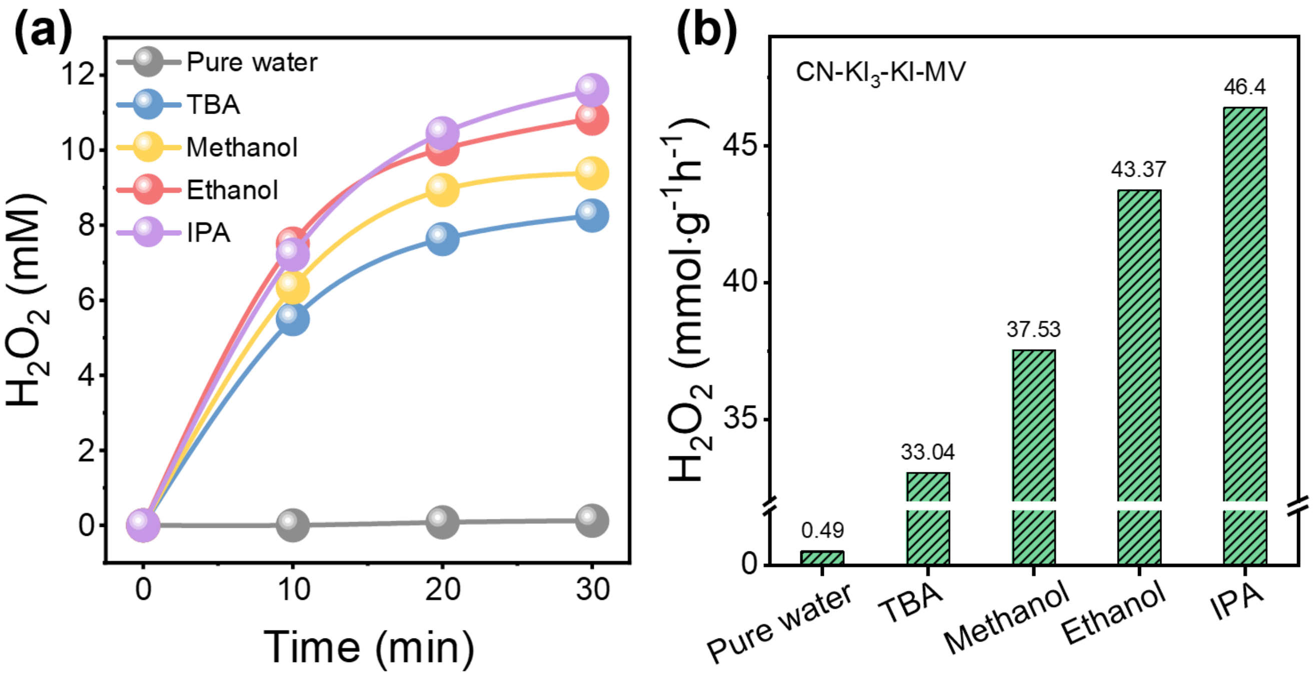

Supplementary Figure 31 | H_2O_2 production activity in different solvents. (a) Influence of small organic molecules on photocatalytic H_2O_2 production (b) and corresponding evolution rates.

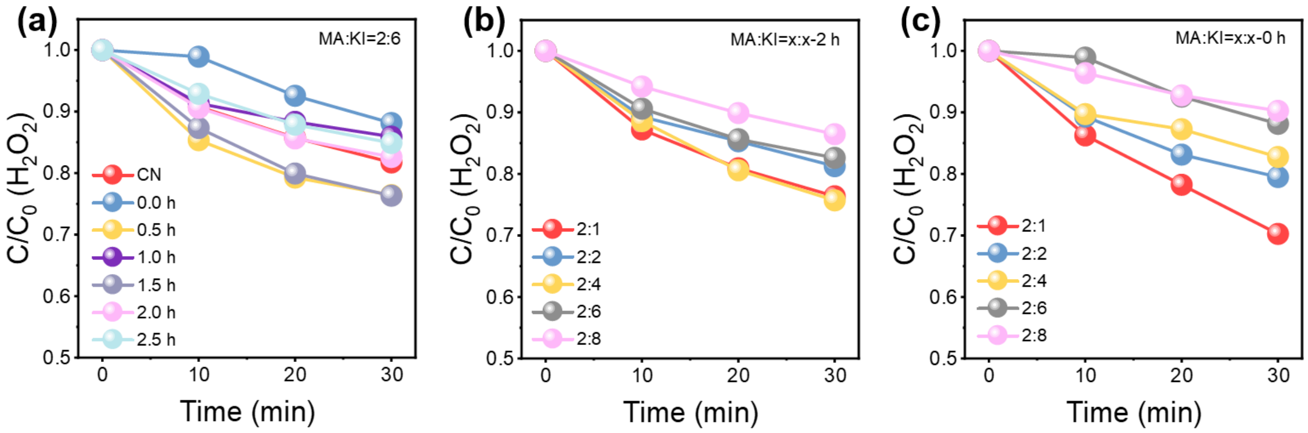

Supplementary Figure 32 | H_2O_2 decomposition activity of the as-prepared samples with (a) different photocatalytic oxidation durations and (b, c) different MA-KI ratios.

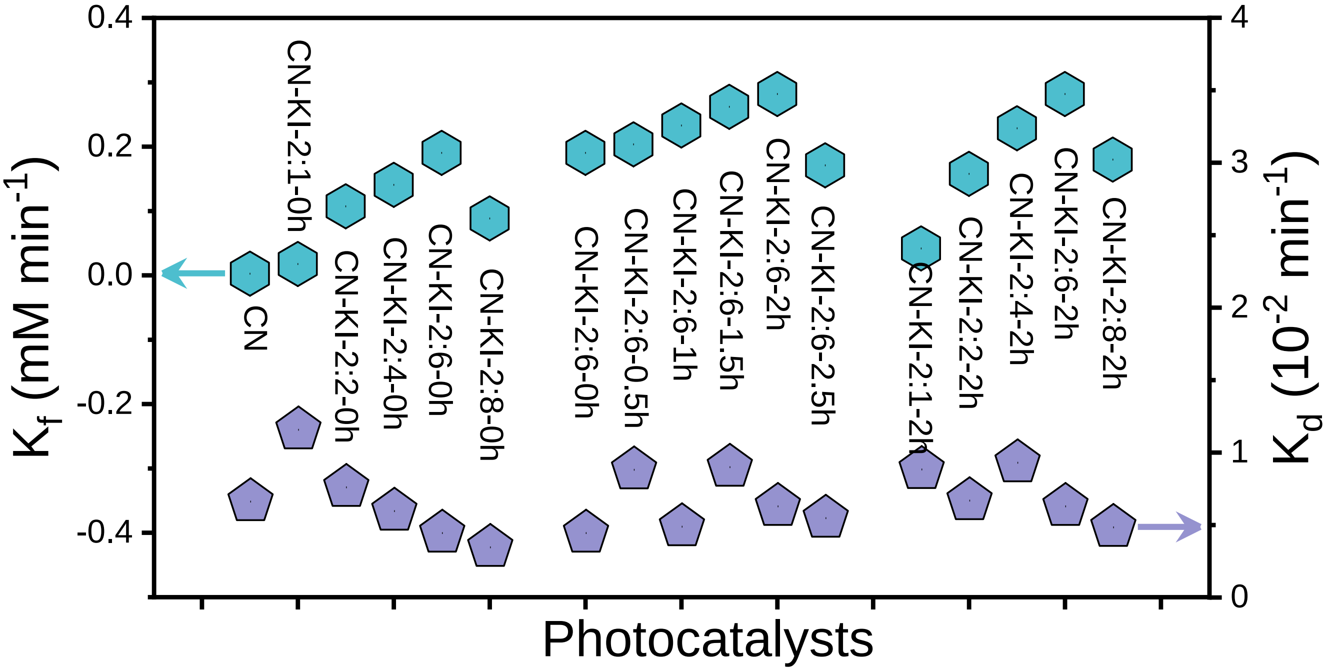

Supplementary Figure 33 | Formation (K_f) and decomposition (K_d) rate constants of the as-prepared samples.

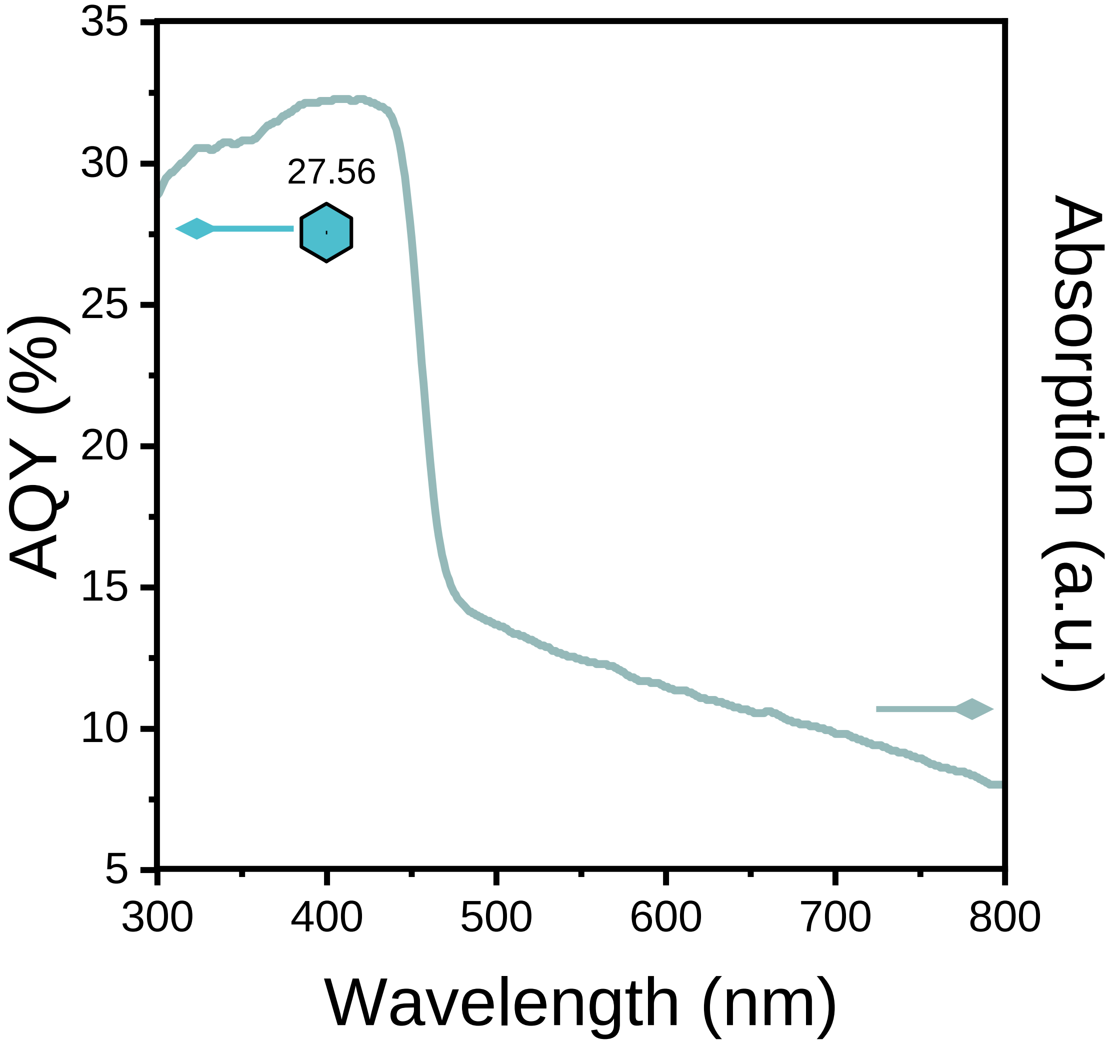

Supplementary Figure 34 | AQY of CN-KI_3-KI-MV and the corresponding UV-vis DRS spectra.

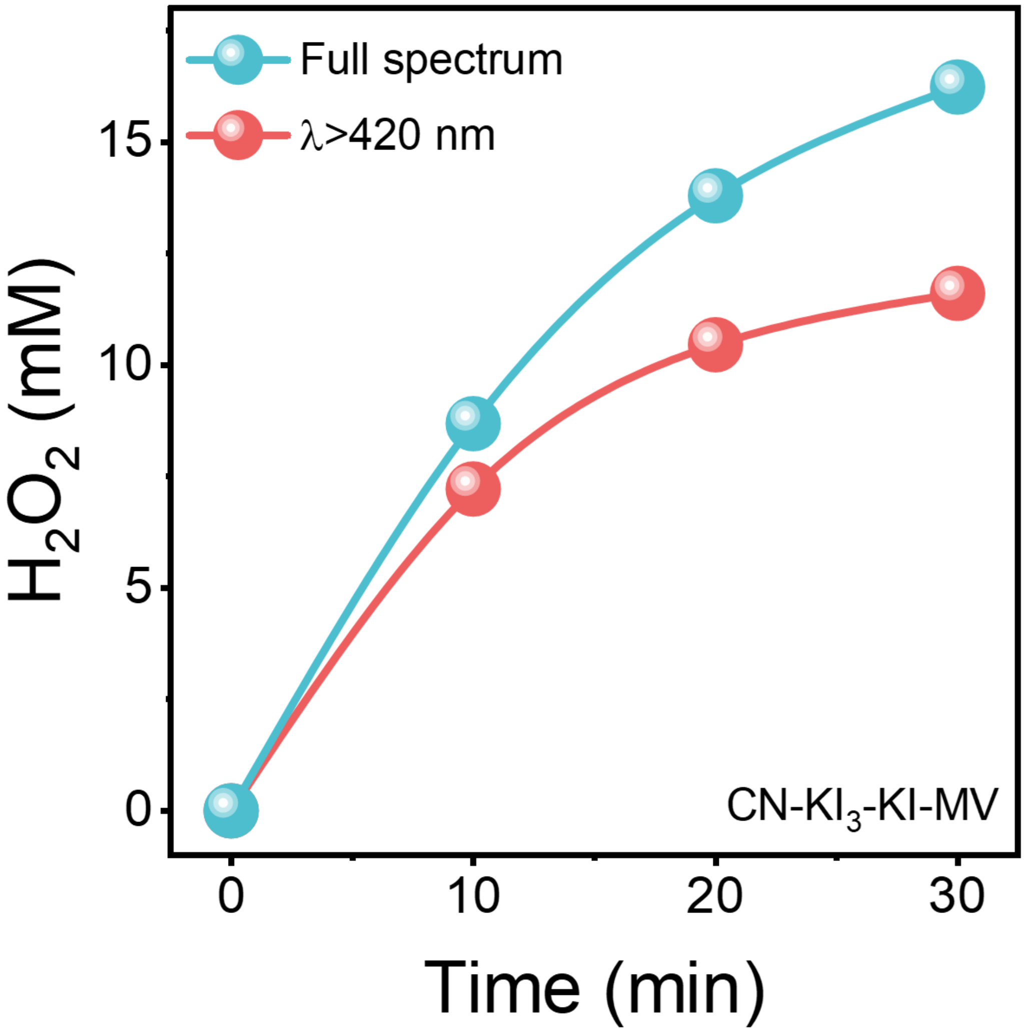

Supplementary Figure 35 | Photocatalytic activities of CN-KI_3-KI-MV for H_2O_2 production under full-spectrum light irradiation.

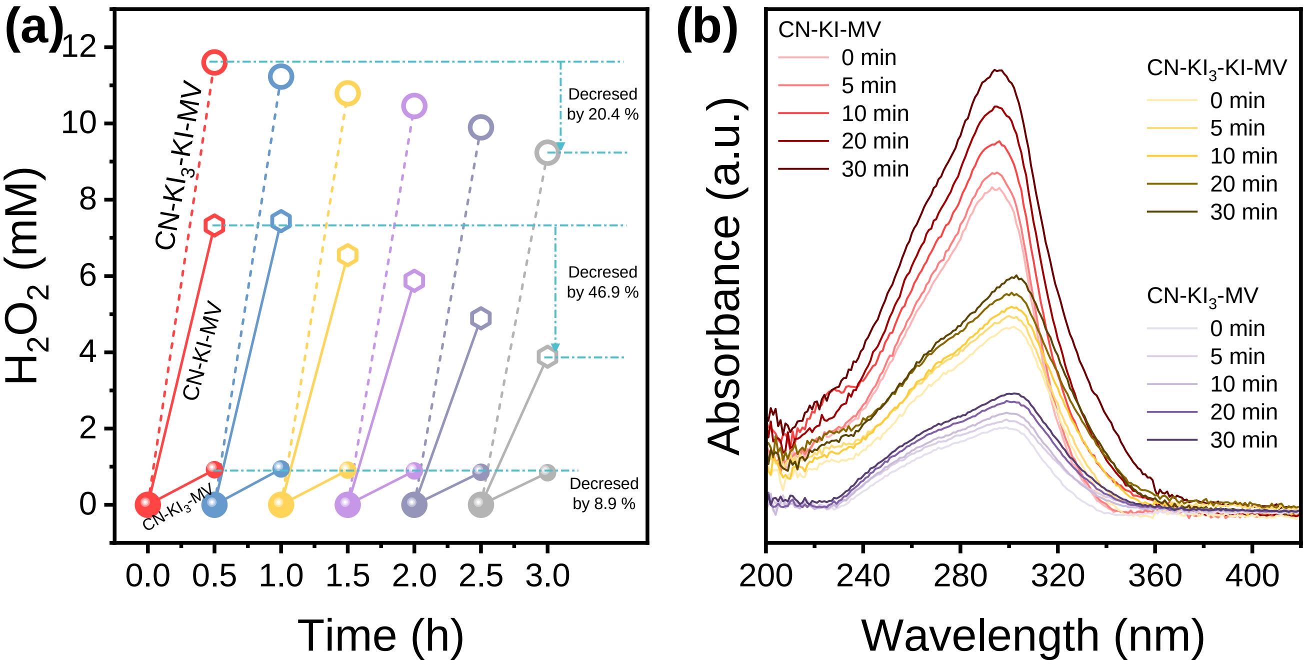

Supplementary Figure 36 | Cycling runs of photocatalytic H_2O_2 production by (a) CN-KI-MV, CN-KI_3-MV, and CN-KI_3-KI-MV and (b) the UV-vis absorption spectra during the reaction process.

Despite the high initial H_2O_2 yield of the CN-KI_3-KI-MV system, which remained above 9 mM even after six cycles of use, a notable decrease in production efficiency over time was observed.

To investigate the cyclic stability and understand the underlying causes of the reduced activity, we conducted semi-in-situ UV-visible absorption spectroscopy. This analysis focused on monitoring the dissolution of iodine species during the reaction process in the CN-KI_3-MV, CN-KI-MV, and CN-KI_3-KI-MV systems (Supplementary Figure **36**). Post-six reaction cycles, the H_2O_2 yields for these systems decreased by 8.9%, 46.95%, and 20.4%, respectively. These results indicated that the incorporation of I_3^- played a dual role: enhancing photocatalytic H_2O_2 production by forming a redox mediator with I^- and contributing to the stabilization of the catalyst.

The mechanism behind this stability enhancement was further elucidated through *semi-in-situ* UV-vis absorption spectroscopy. The absorption observed in the 280-320 nm range was attributed to the presence of I_3^- ions. The emergence of I_3^- signals in the CN-KI-MV system was linked to the mutual transformation between the I^-/I_3^- redox mediators during the photocatalytic reaction. The addition of I_3^- ions resulted in significantly reduced dissolution of iodine species in the CN-KI_3-KI system compared to CN-KI, thereby enhancing the catalyst's stability. However, the issue of iodine species dissolution was not entirely resolved in the CN-KI_3-KI-MV system, which likely contributed to the observed decline in H_2O_2 production in the later stages of cyclic experiments. This finding underscores the importance of optimizing iodine species concentrations to balance photocatalytic efficiency and catalyst stability.

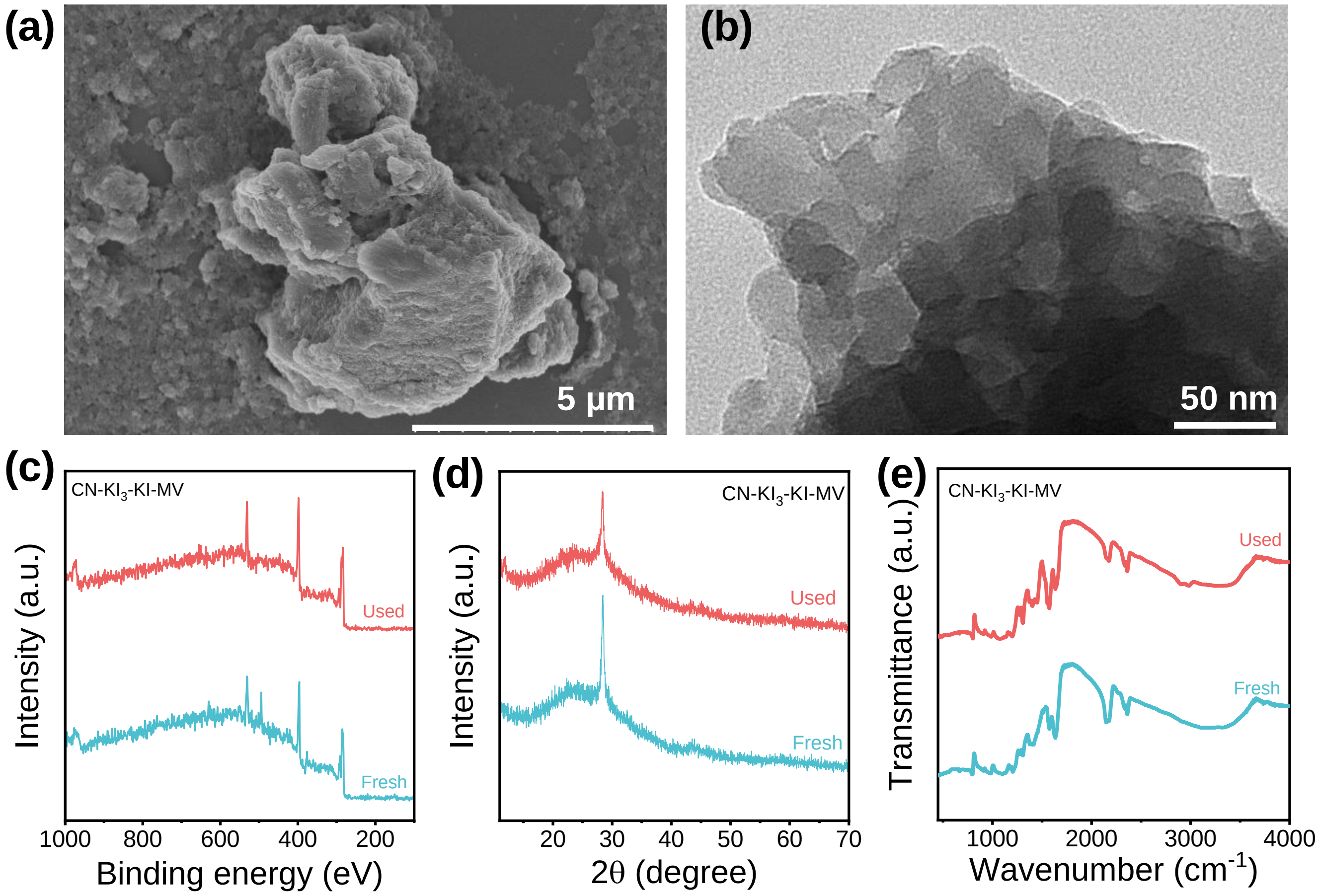

Supplementary Figure 37 | Stability assessment of CN-KI_3-KI-MV. (a) SEM images, (b) TEM images, (c) XPS spectra, (d) XRD patterns, and (e) FTIR analysis of CN-KI_3-KI-MV after the photocatalytic reaction.

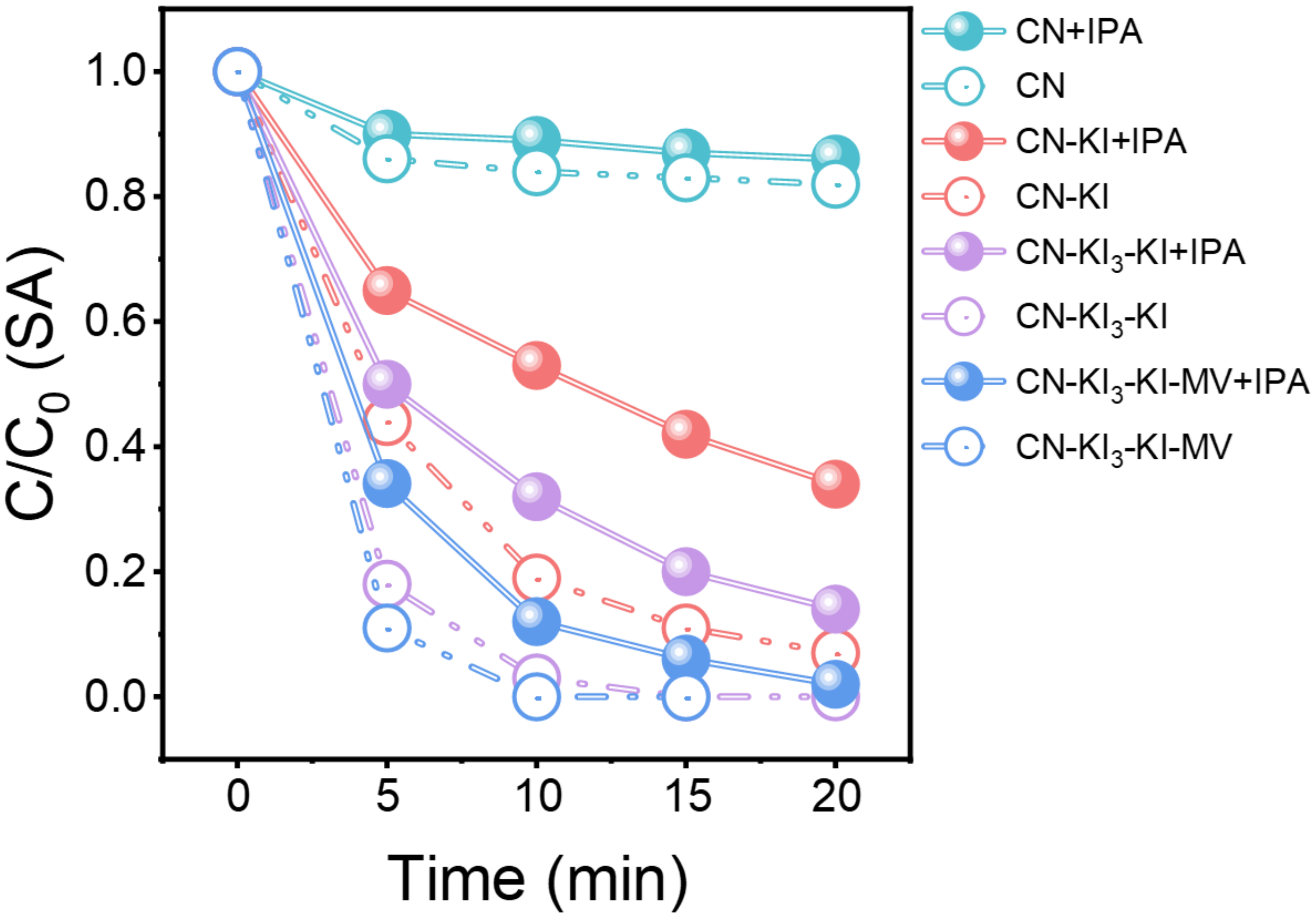

Supplementary Figure 38 | Photocatalytic degradation performance using CN, CN-KI, CN-KI_3-KI, and CN-KI_3-KI-MV as the photocatalysts.

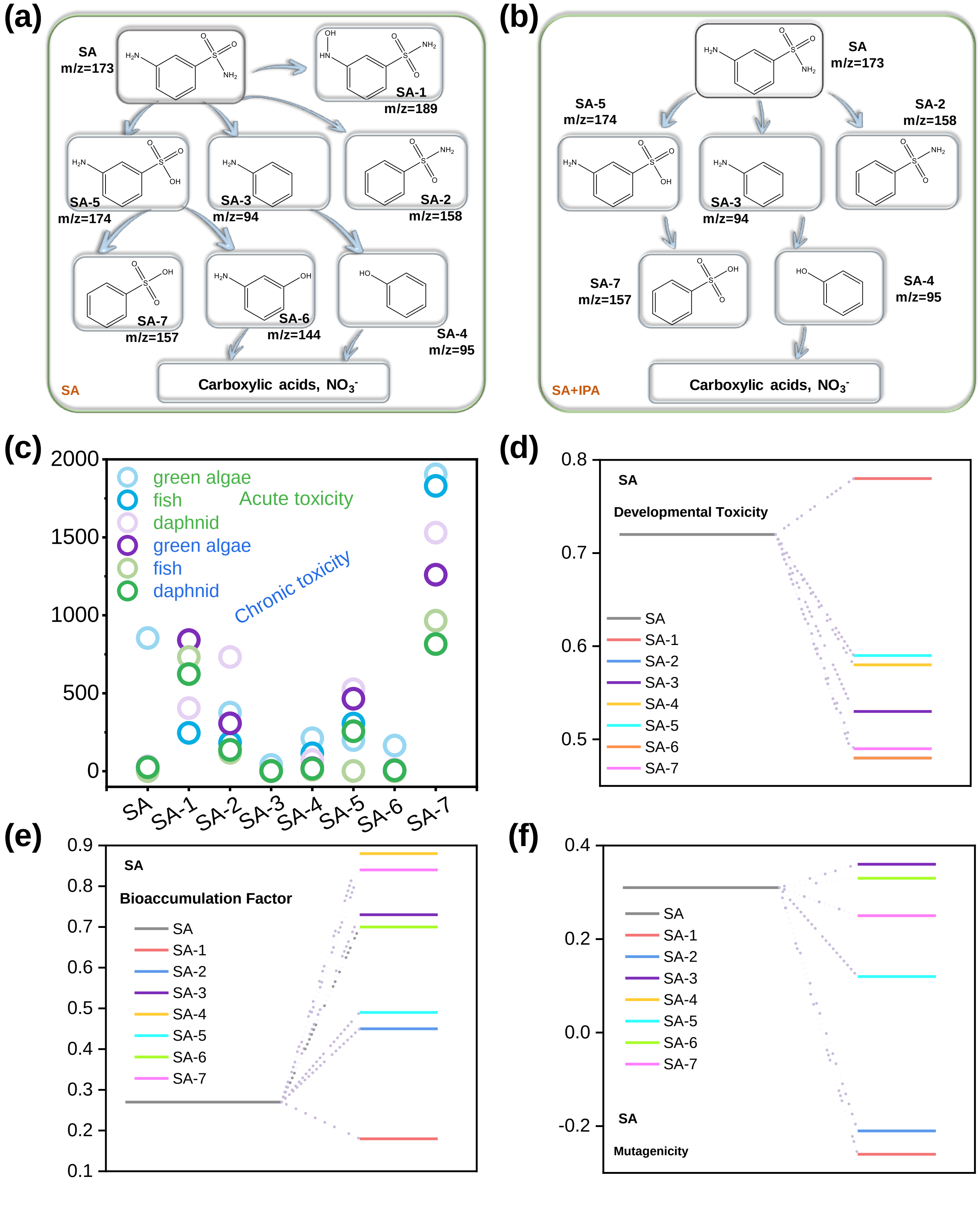

Supplementary Figure 39 | Intermediates of SA degradation and their toxicity evaluation. (a-b) Degradation pathways of SA in the CN-KI$_3$-KI-MV system. (c) Toxicity evaluation of the intermediates. (d-f) Toxicity analysis of SA and its intermediates using T.E.S.T software.

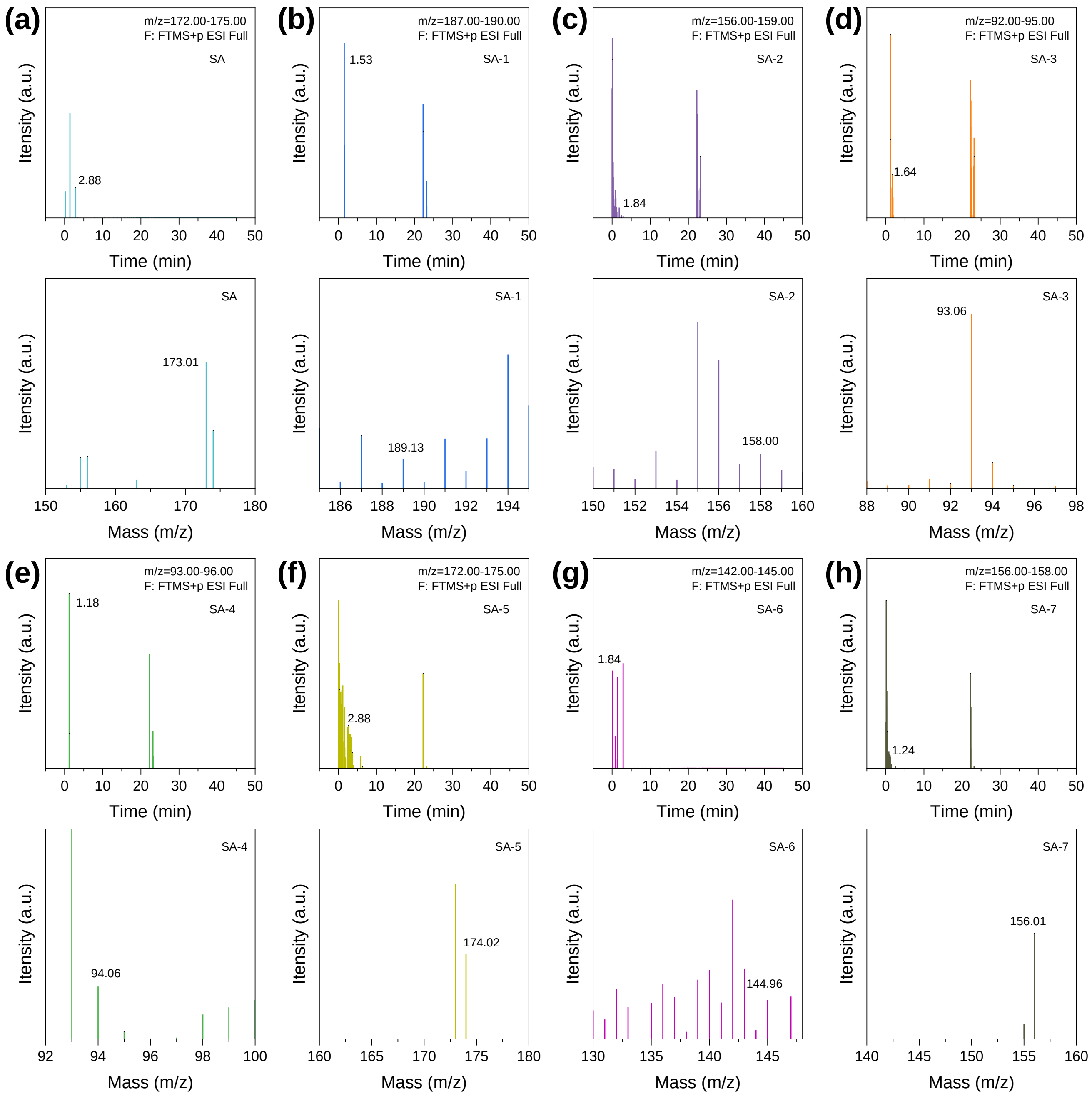

Supplementary Figure 40 | (a-h) LC-MS analysis of SA and its intermediates.

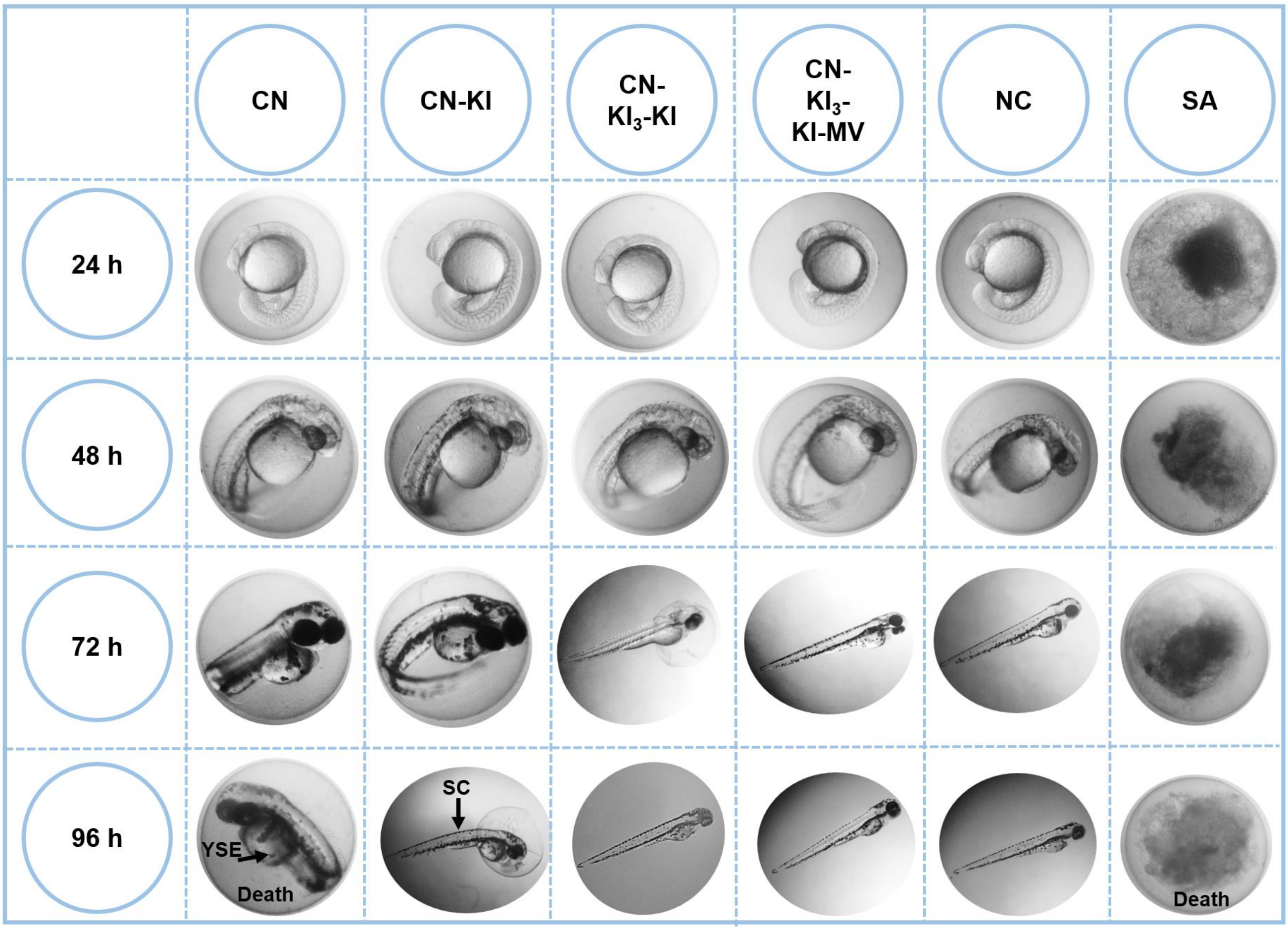

Supplementary Figure 41 | Cultivation experiments of zebrafish using samples from different systems.

Zebrafish cultivation experiments were conducted using SA solutions detoxified by different catalysts to evaluate the detoxification efficacy of the designed catalytic system in water. Among the tested catalytic systems, only the CN-KI_3-KI-MV system exhibited a zebrafish embryonic development process consistent with the NC group. The CN system exhibited yolk sac edema (YSE) teratogenic symptoms and lethal symptoms, CN-KI showed developmental delays and spinal curvature (SC) teratogenic symptoms, and the CN-KI_3-KI system displayed developmental delays. The

embryonic development in different catalytic systems and their respective mineralization rates of SA showed a positive correlation, confirming the robust detoxification performance of the CN-KI_3-KI-MV system in polluted water. This high detoxification efficiency was attributed to the enhanced photocatalytic activity of CN-KI_3-KI-MV, resulting from the embedded I^-/I_3^- redox mediator and the external electric field established by externally attached MV.

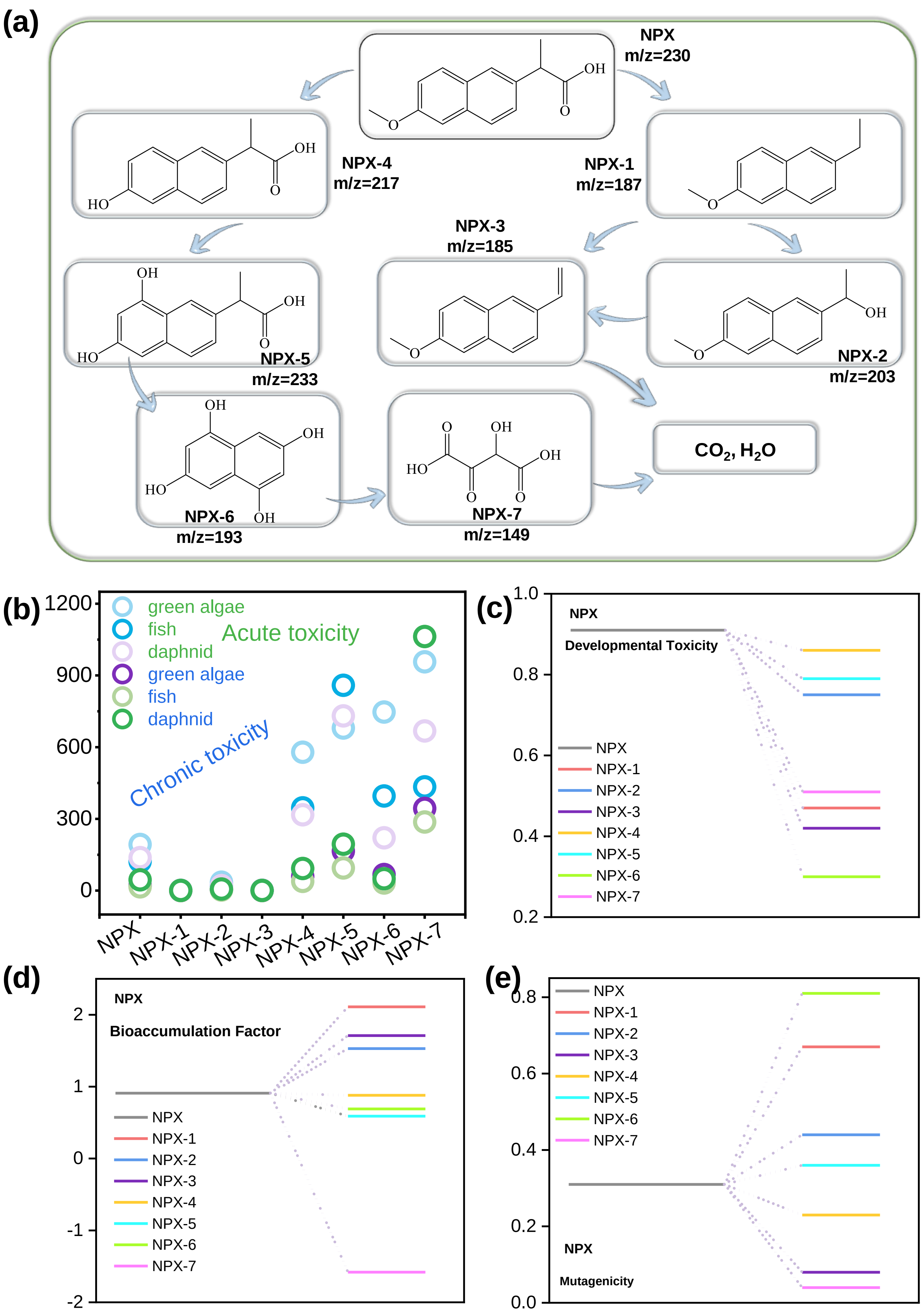

(a)
NPX
m/z=230
NPX-4
m/z=217
NPX-1
m/z=187
NPX-3
m/z=185
NPX-5
m/z=233
NPX-2
m/z=203
NPX-6
m/z=193
NPX-7
m/z=149
CO_2, H_2O
(b)
green algae
fish
daphnid
Acute toxicity
green algae
fish
daphnid
Chronic toxicity
NPX NPX-1 NPX-2 NPX-3 NPX-4 NPX-5 NPX-6 NPX-7
(c)
NPX
Developmental Toxicity
(d)
NPX
Bioaccumulation Factor
(e)
NPX
Mutagenicity
NPX
NPX-1
NPX-2
NPX-3
NPX-4
NPX-5
NPX-6
NPX-7

Supplementary Figure 42 | Intermediates of NPX degradation and their toxicity evaluation. (a) Degradation pathways of NPX in the CN-KI_3-KI-MV system. (b) Toxicity evaluation of different intermediates. (c-e) Toxicity analysis of NPX and its intermediates using T.E.S.T software.

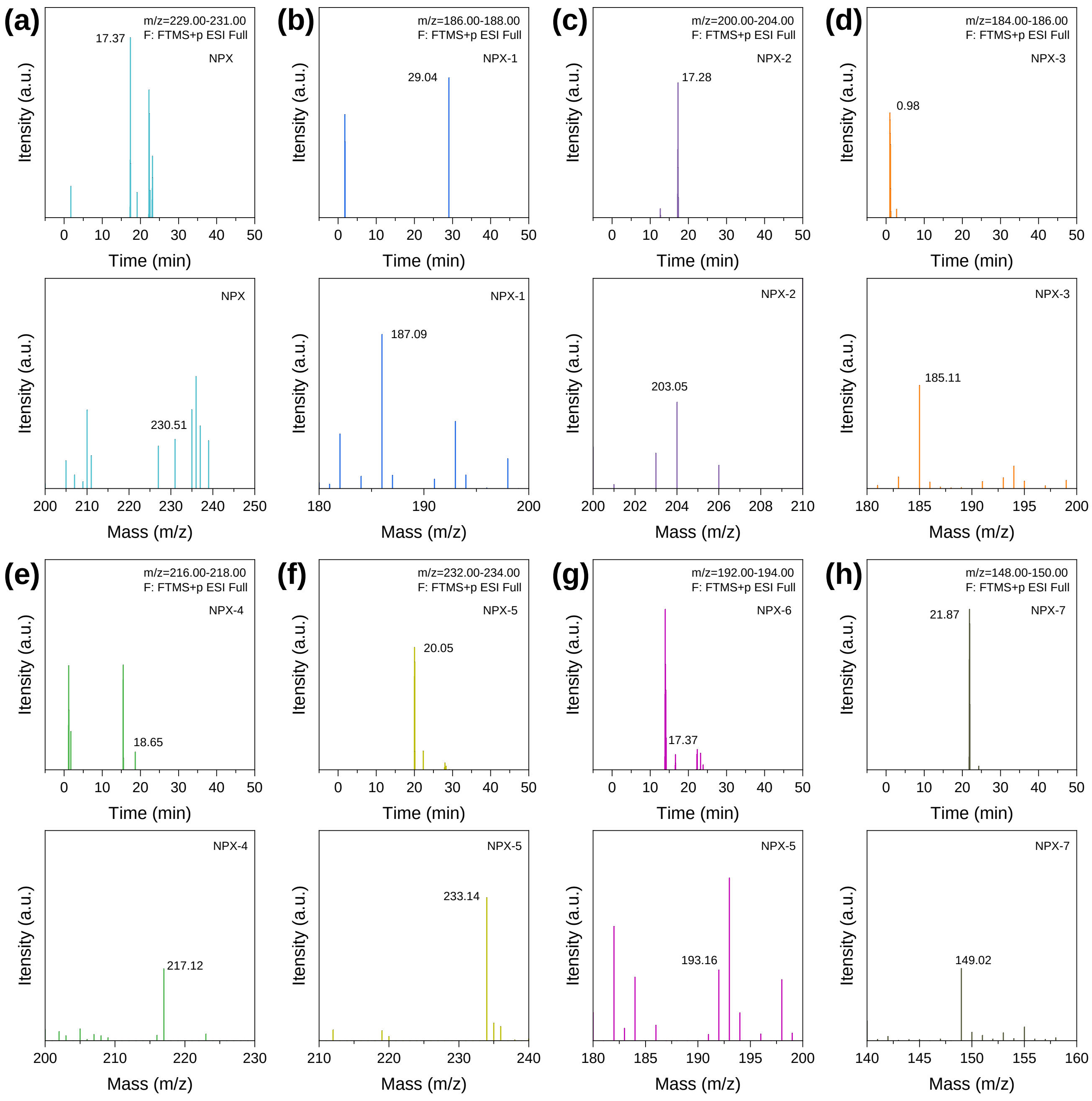

Supplementary Figure 43 | (a-h) LC-MS analysis of NPX and its intermediates.

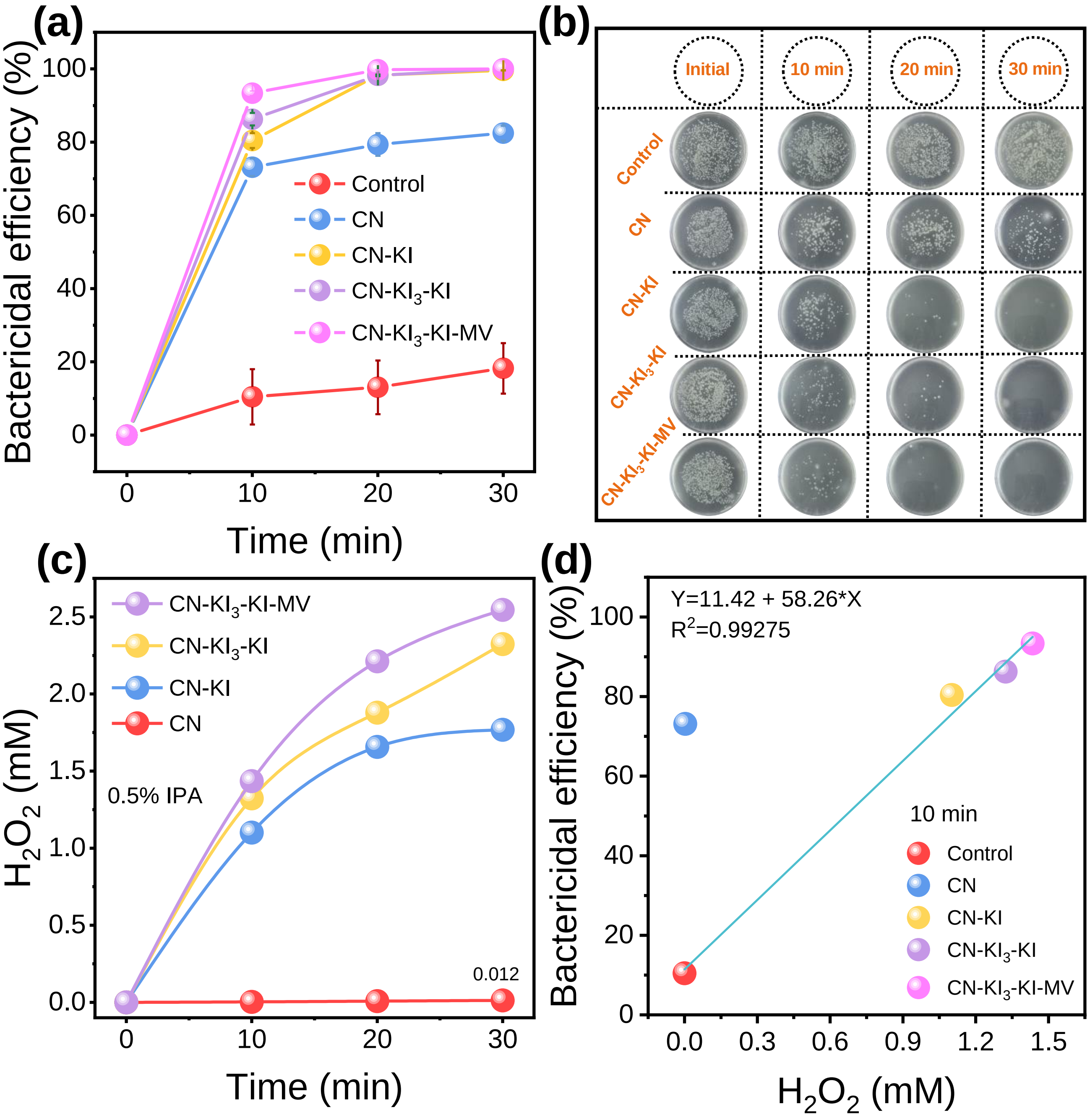

Supplementary Figure 44 | In-situ antimicrobial performance evaluation. (a) Comparison of the disinfection effects of the prepared samples on *E. coli/* cells. (b) Corresponding bacterial counts of *E. coli.* at 0, 10, 20, and 30 min. (c-d) The H_2O_2 production of the prepared samples and their fitting functions with antibacterial efficiency.

The *in-situ* disinfection efficacy of various photocatalytic systems was thoroughly evaluated and is depicted in Supplementary Figure **44**. When compared to the control group, which consisted solely of a 0.5% isopropanol (IPA) solution, the meticulously engineered photocatalytic systems exhibited significantly enhanced disinfection capabilities. Among these systems, the CN-KI_3-KI-MV configuration was notably effective, achieving near-complete eradication (approximately 100%) of the bacterial population within just 10 minutes.

A statistical analysis was conducted to establish a relationship between the antibacterial efficiency and the concentration of H_2O_2 produced within each system. This analysis revealed a substantial positive correlation between these two parameters. Such a correlation is indicative of the fact that the superior disinfection performance observed in the CN-KI_3-KI-MV system, particularly against Escherichia coli, can be attributed to a synergistic mechanism. This mechanism encompasses both the inherent photocatalytic disinfection capability of the catalyst and the H_2O_2 produced during the photocatalytic process.

The ability of the CN-KI_3-KI-MV system to generate H_2O_2 effectively, coupled with its photocatalytic properties, contributes to its high antibacterial efficiency. This finding highlights the potential of such photocatalytic systems in applications requiring rapid and efficient microbial disinfection, particularly in water treatment and environmental remediation scenarios.

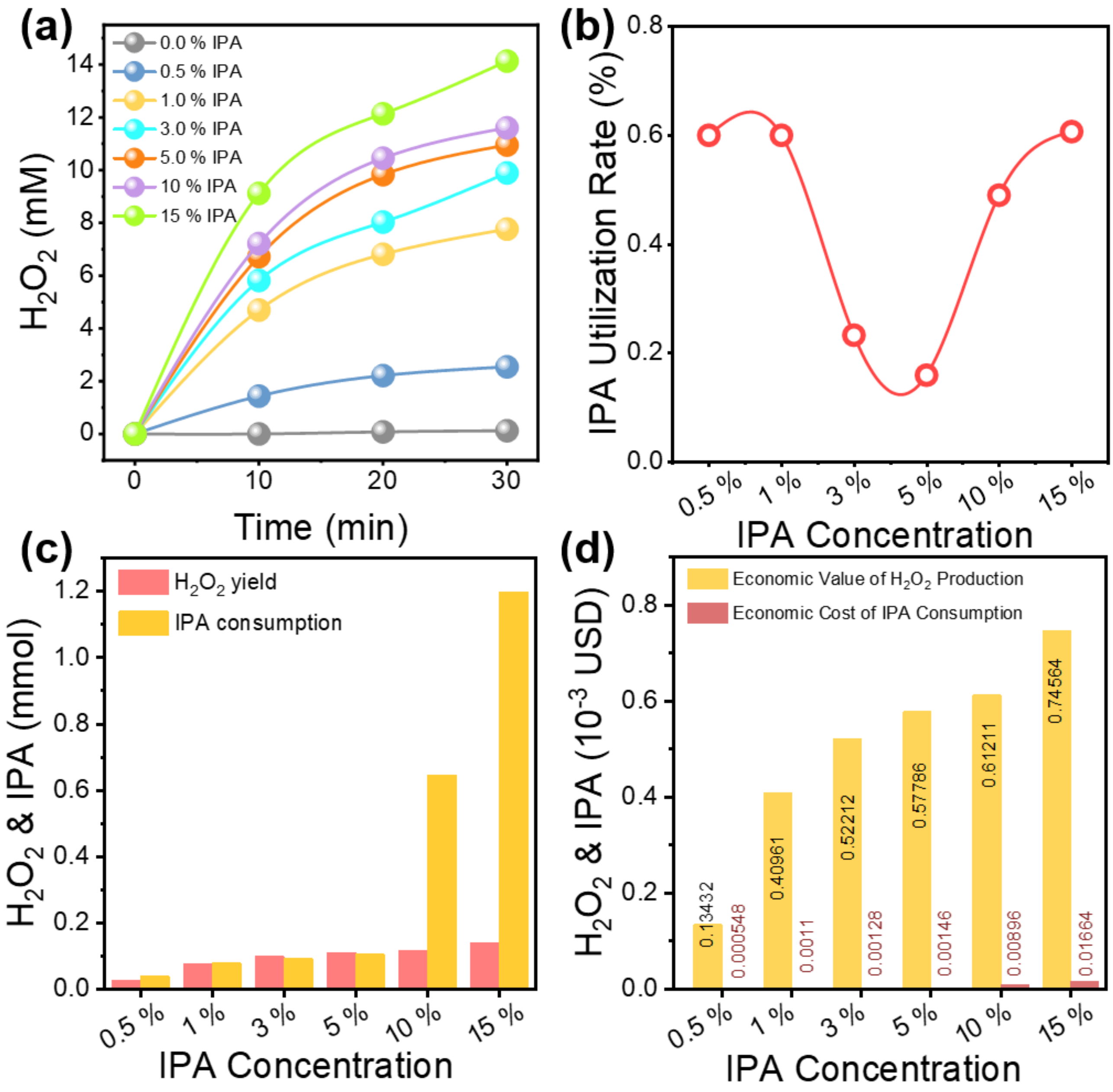

Supplementary Figure 45 | Economic evaluation of the CN-KI$_3$-KI-MV system. (a) the activity of H_2O_2 production across systems varying in IPA concentrations; (b) the utilization rate of IPA; and (c-d) the consumption of IPA and subsequent production of H_2O_2, accompanied by an economic assessment based on the cost of Aladdin reagents.

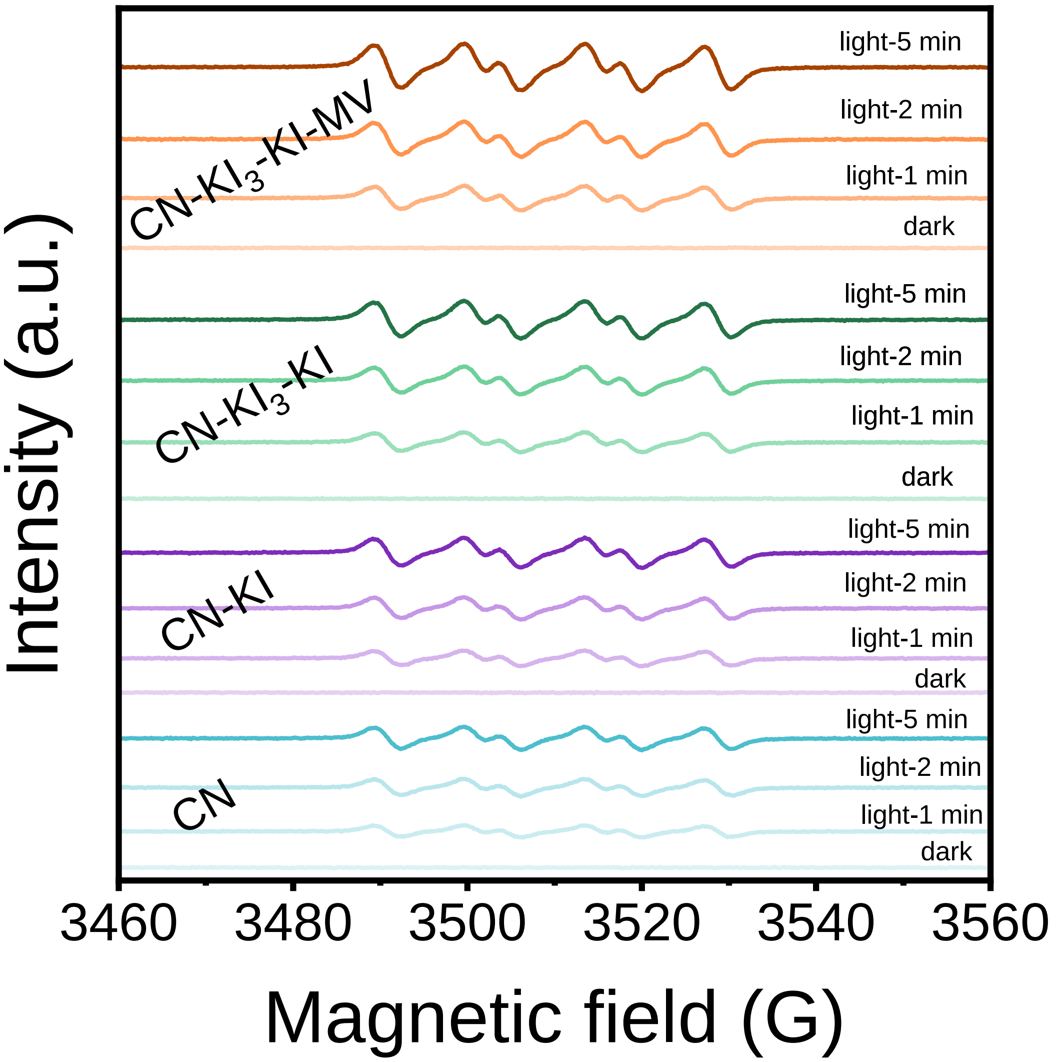

Supplementary Figure 46 | EPR spectra of CN, CN-KI, CN-KI_3-KI, and CN-KI_3-KI-MV.

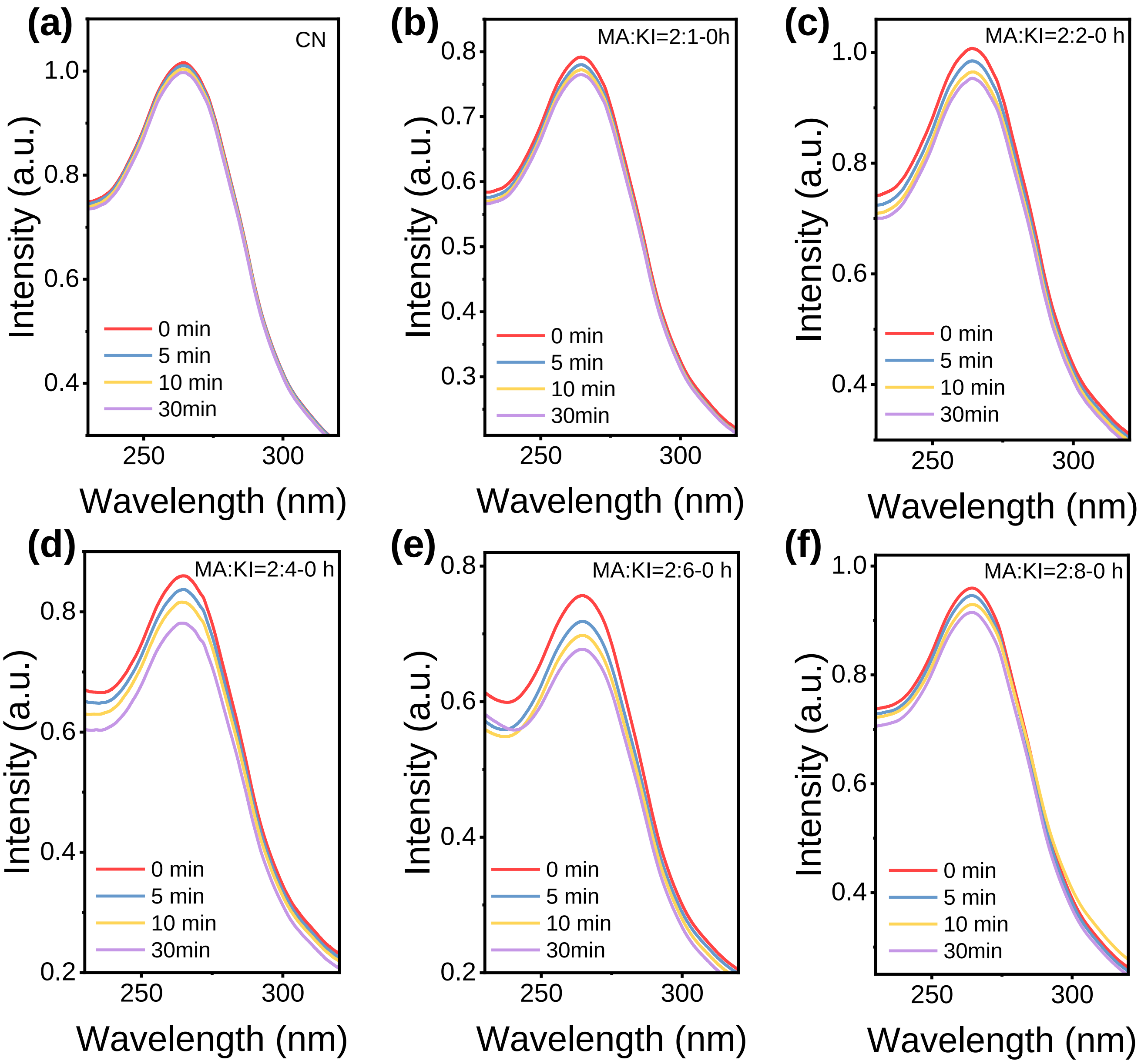

Supplementary Figure 47 | NBT absorption intensities of (a) CN and (b-f) the samples with different MA-KI ratios.

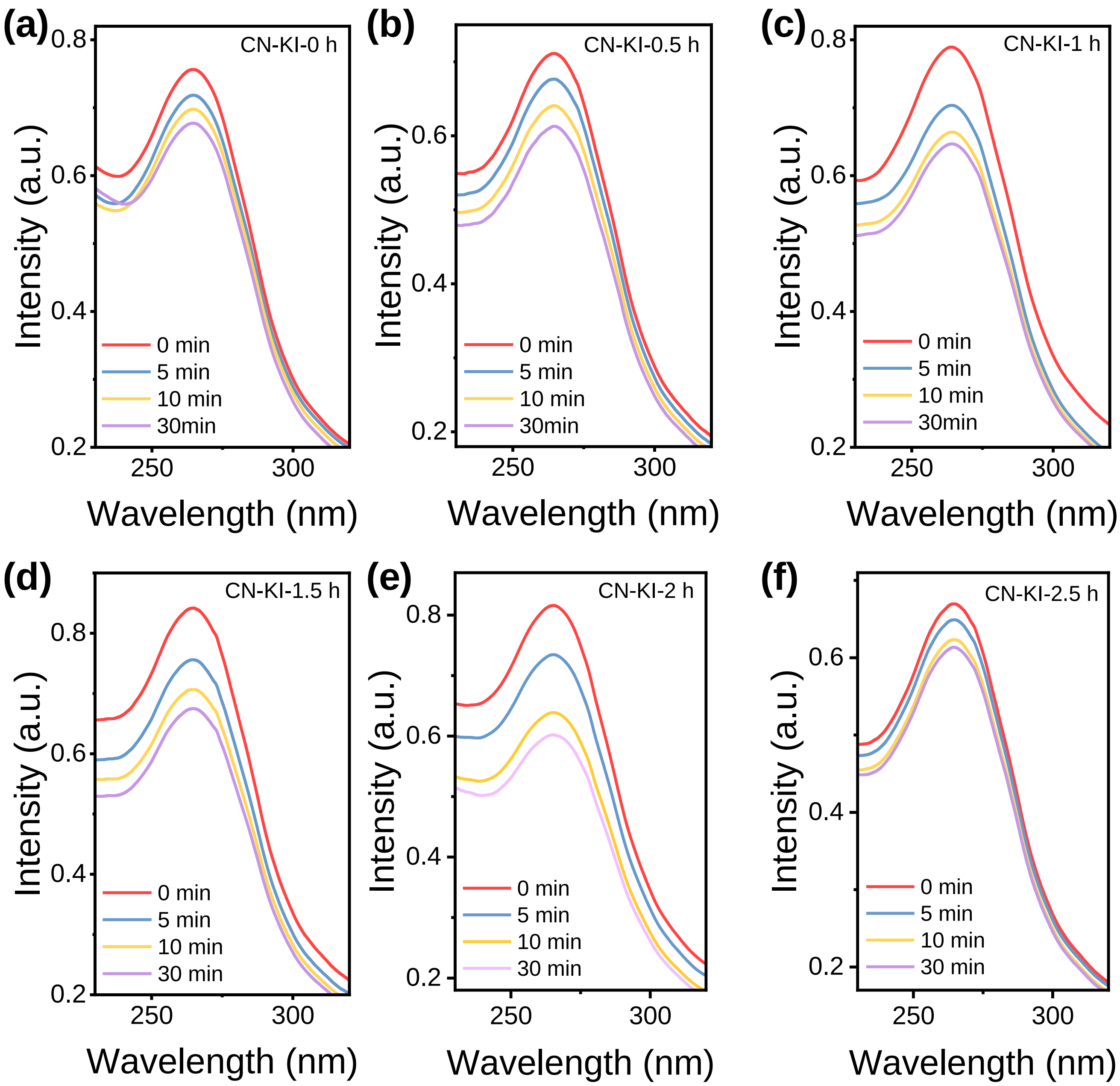

Supplementary Figure 48 | (a-f) NBT absorption intensities of the samples with different photocatalytic oxidation durations (2: 6).

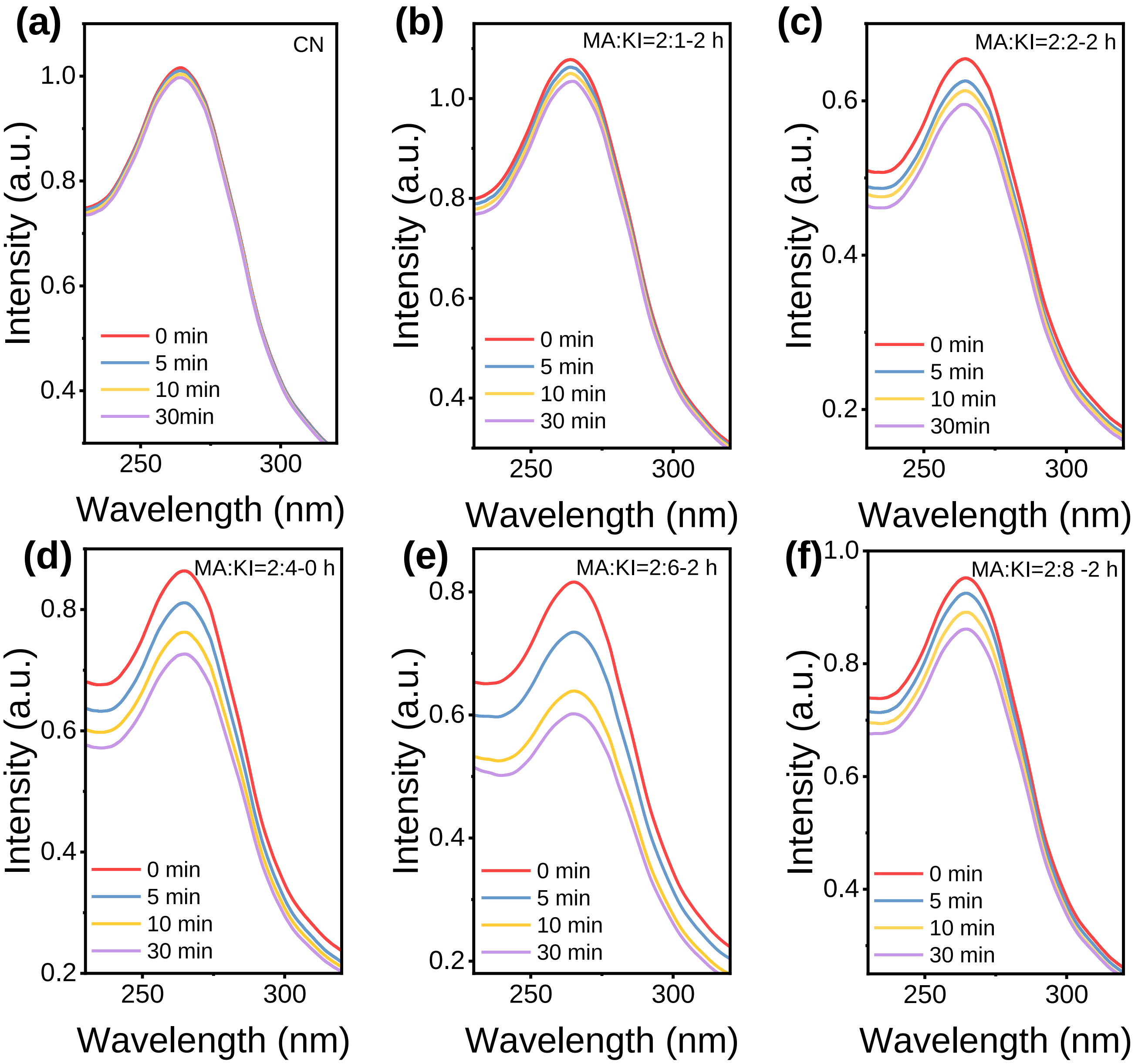

Supplementary Figure 49 | The NBT absorption intensities of (a) CN and (b-f) the samples with different MA-KI ratios (2 h).

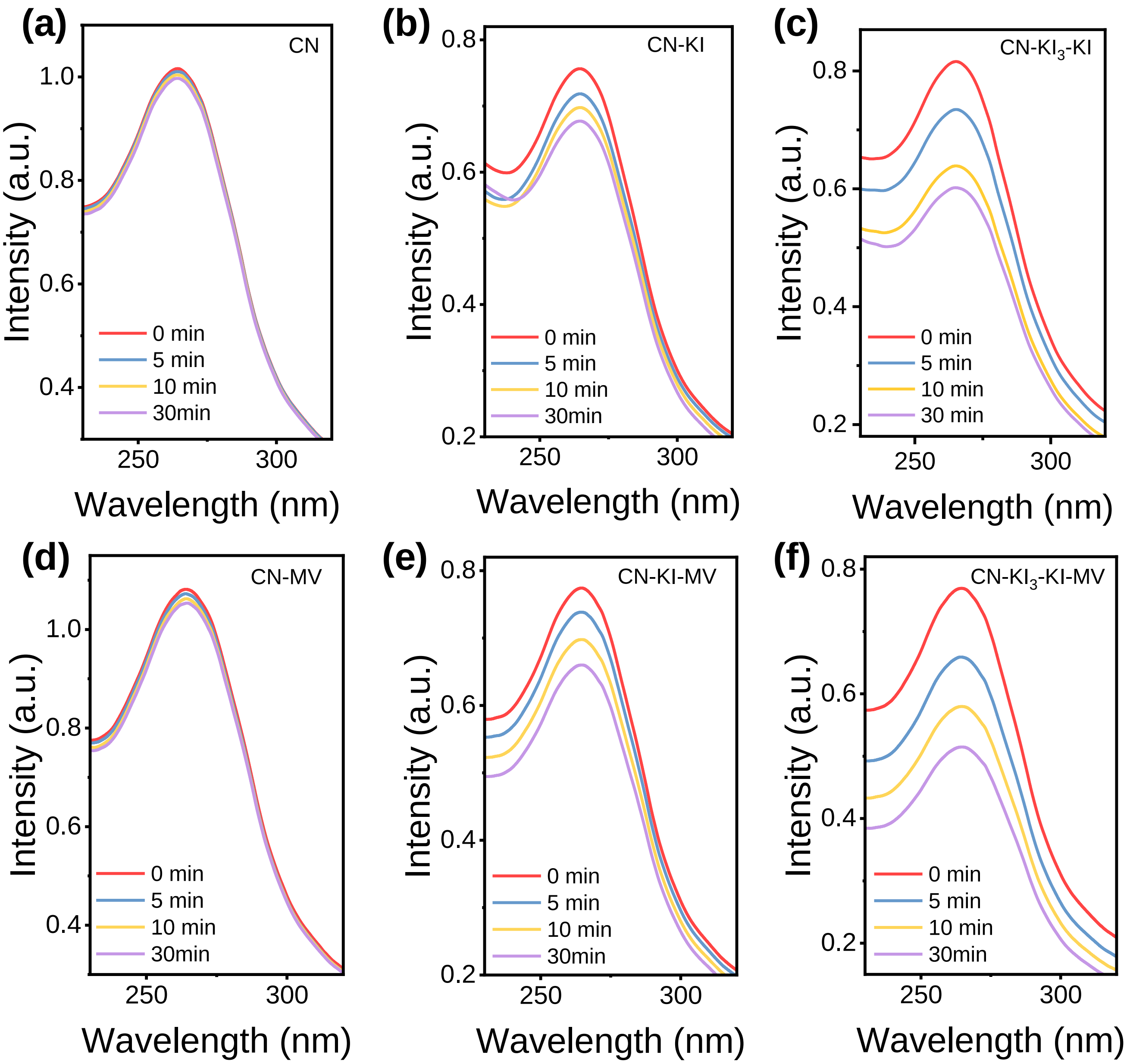

Supplementary Figure 50 | NBT absorption intensities of (a, d) CN, (b, e) CN-KI, and (c, f) CN-KI_3-KI before and after MV loading.

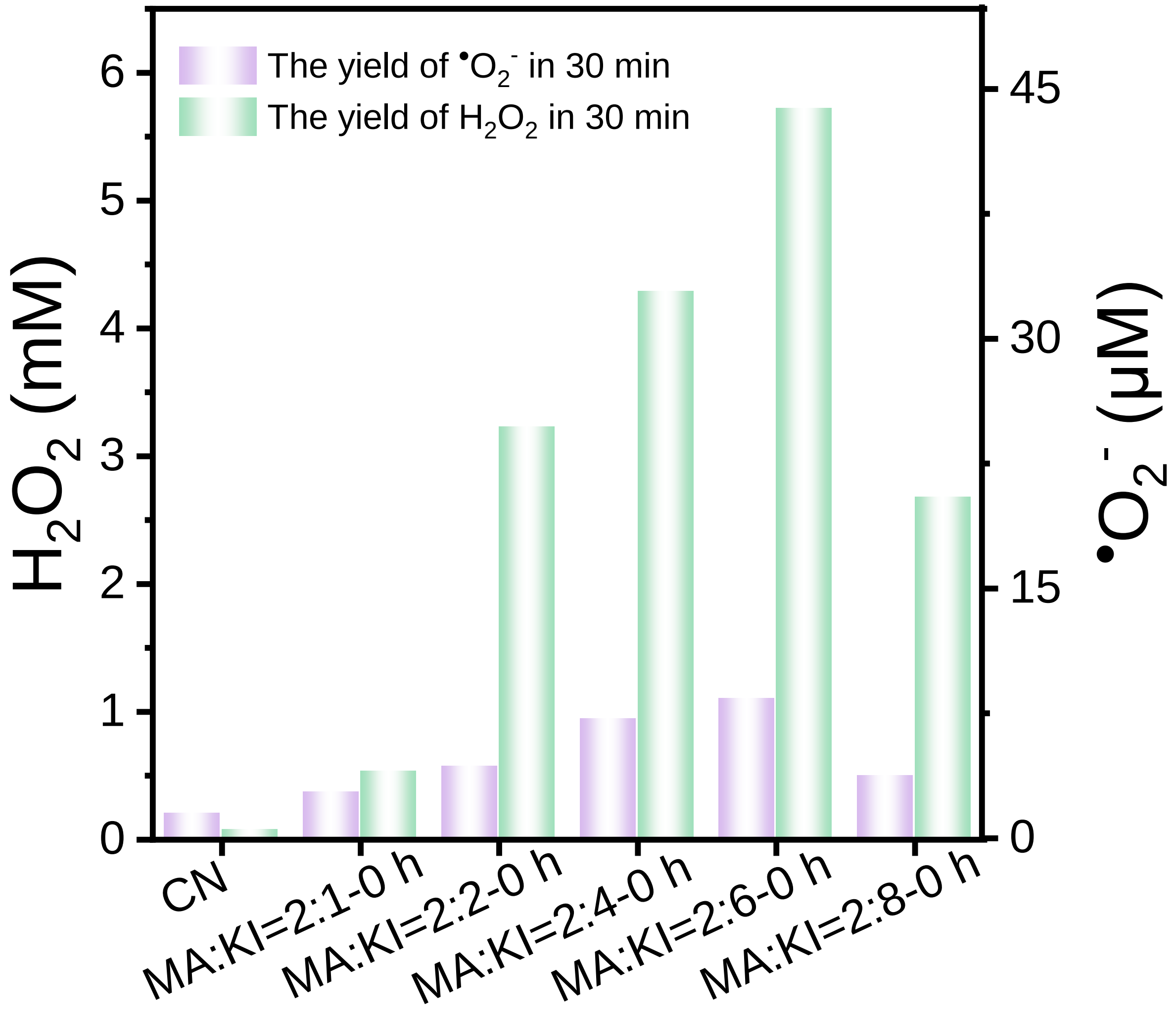

Supplementary Figure 51 | H_2O_2 and $^{\bullet}O_2^-$ yields of CN and the samples with different MA-KI ratios.

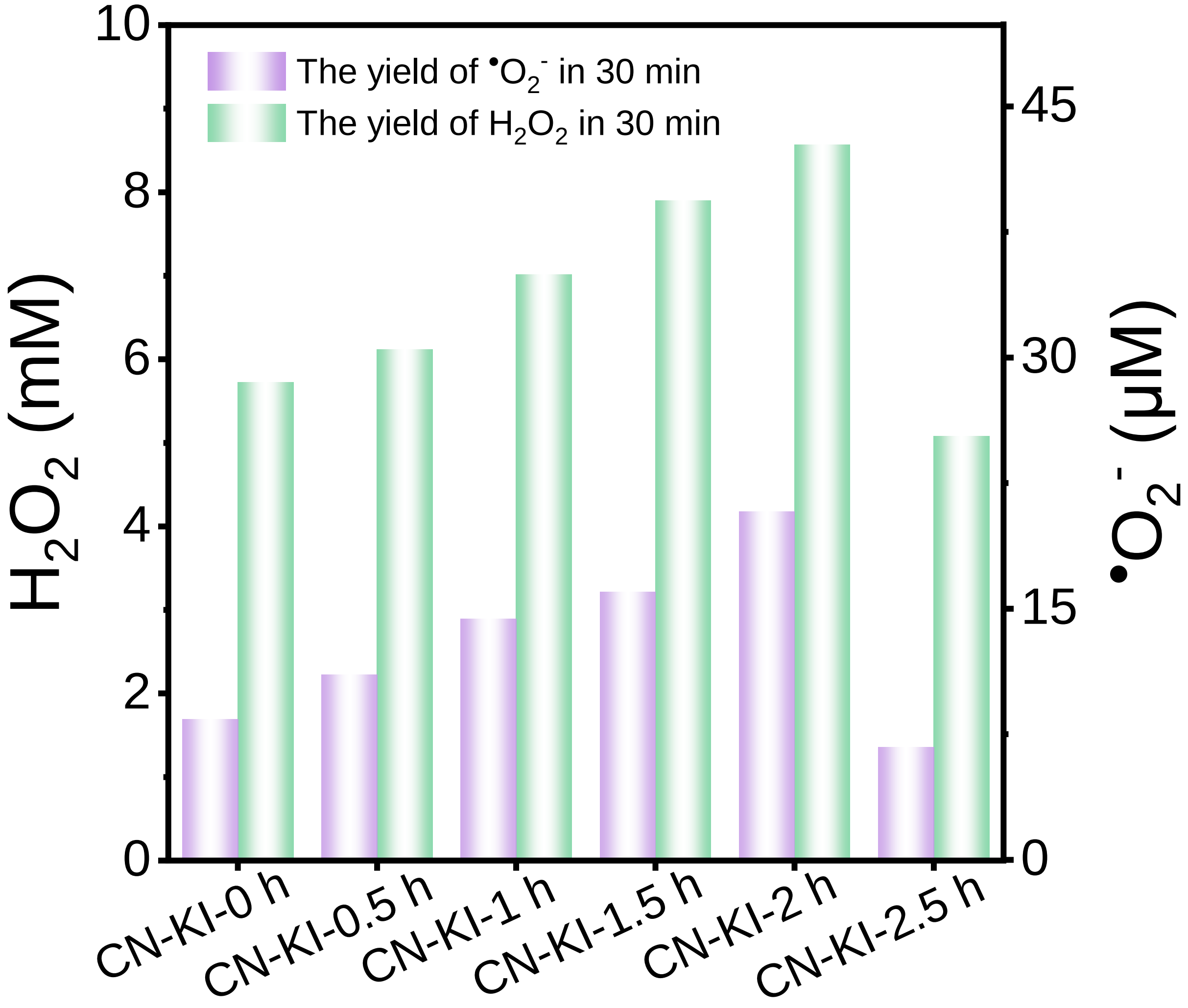

Supplementary Figure 52 | H_2O_2 and $^{\bullet}O_2$ yields with different photocatalytic oxidation durations (2: 6).

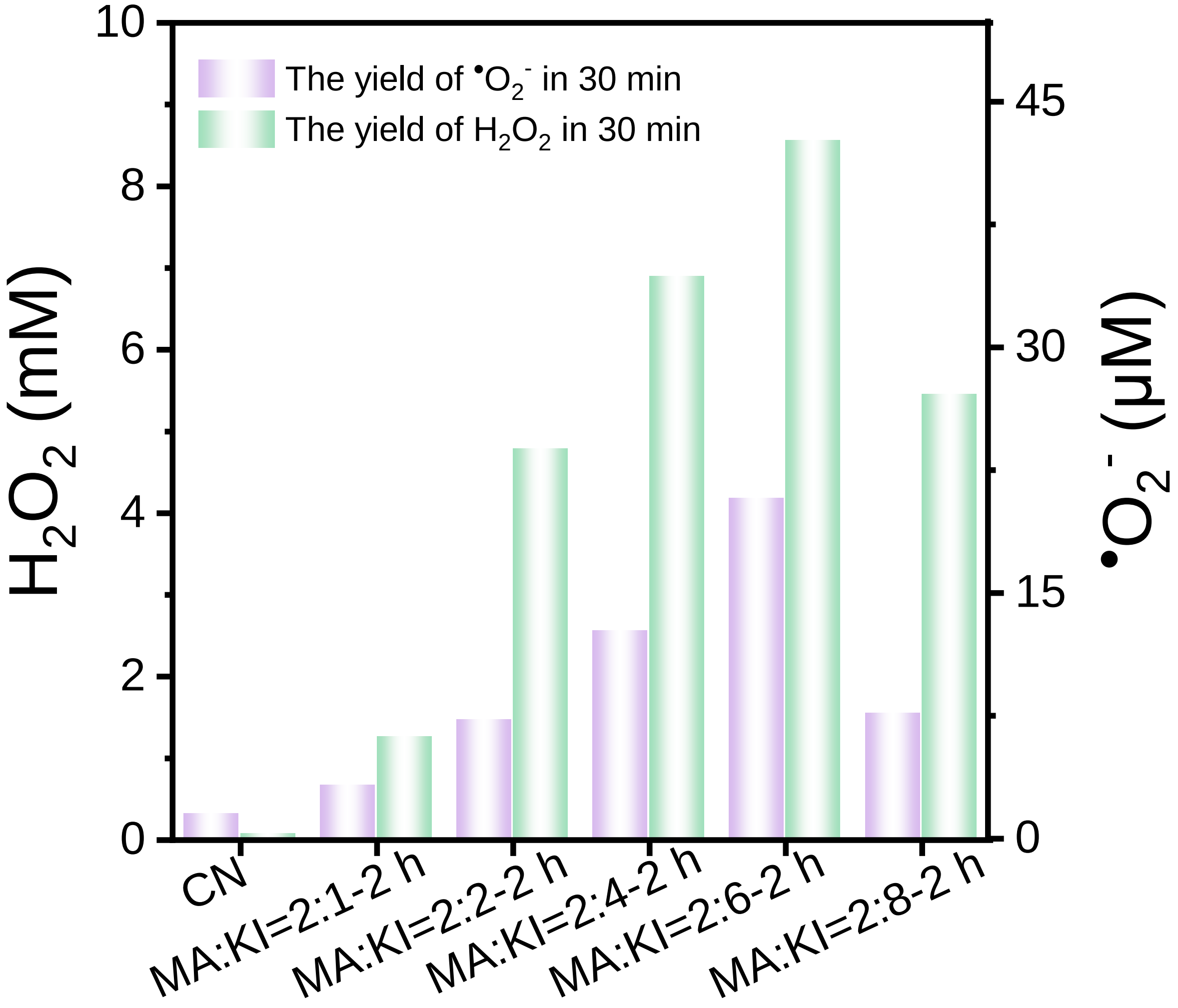

Supplementary Figure 53 | H_2O_2 and $^{\bullet}O_2^-$ yields of CN and the samples with different MA-KI ratios (2 h).

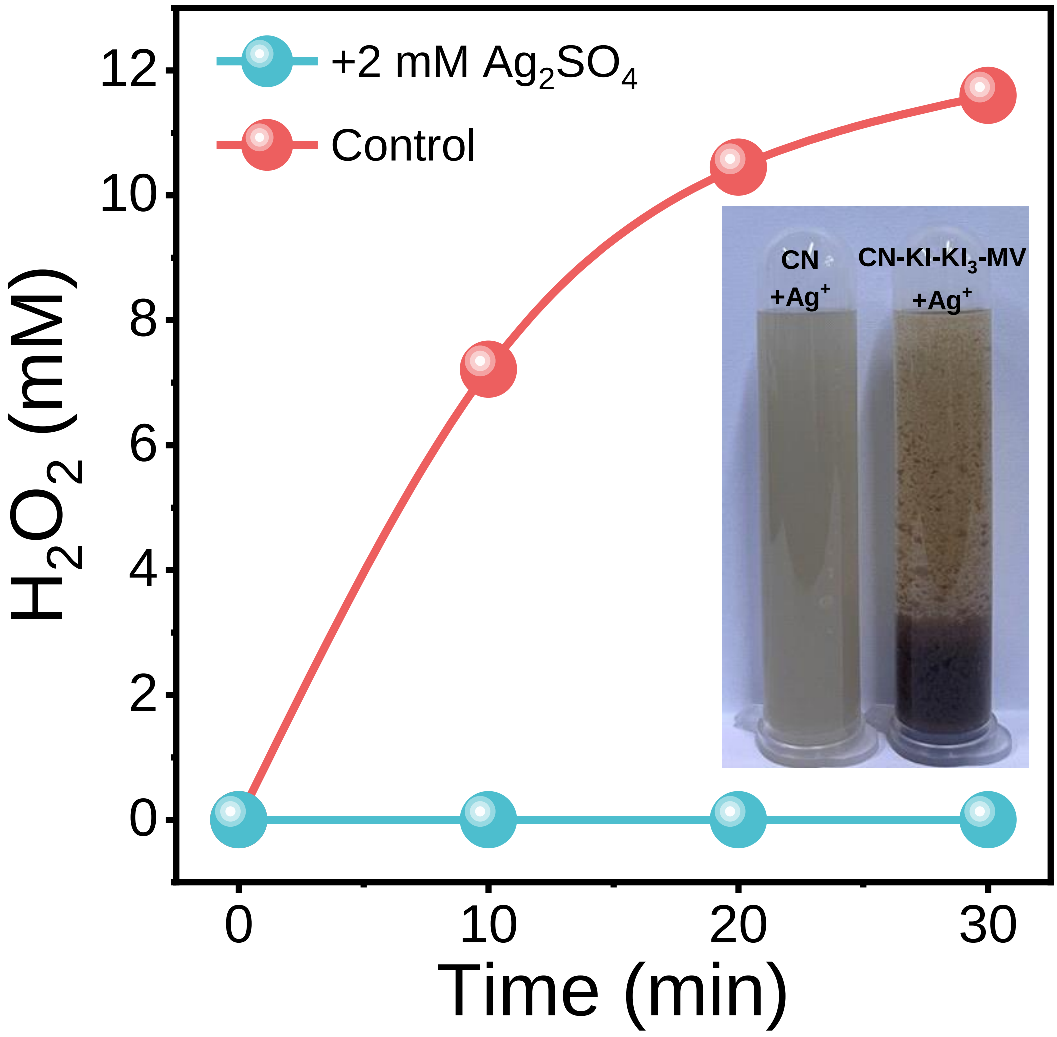

Supplementary Figure 54 | Effect of Ag^{+} on the photocatalytic production of H_2O_2 in CN-KI_3-KI-MV system. Inset: Photographs of Ag^{+} bound with different reaction substrates.

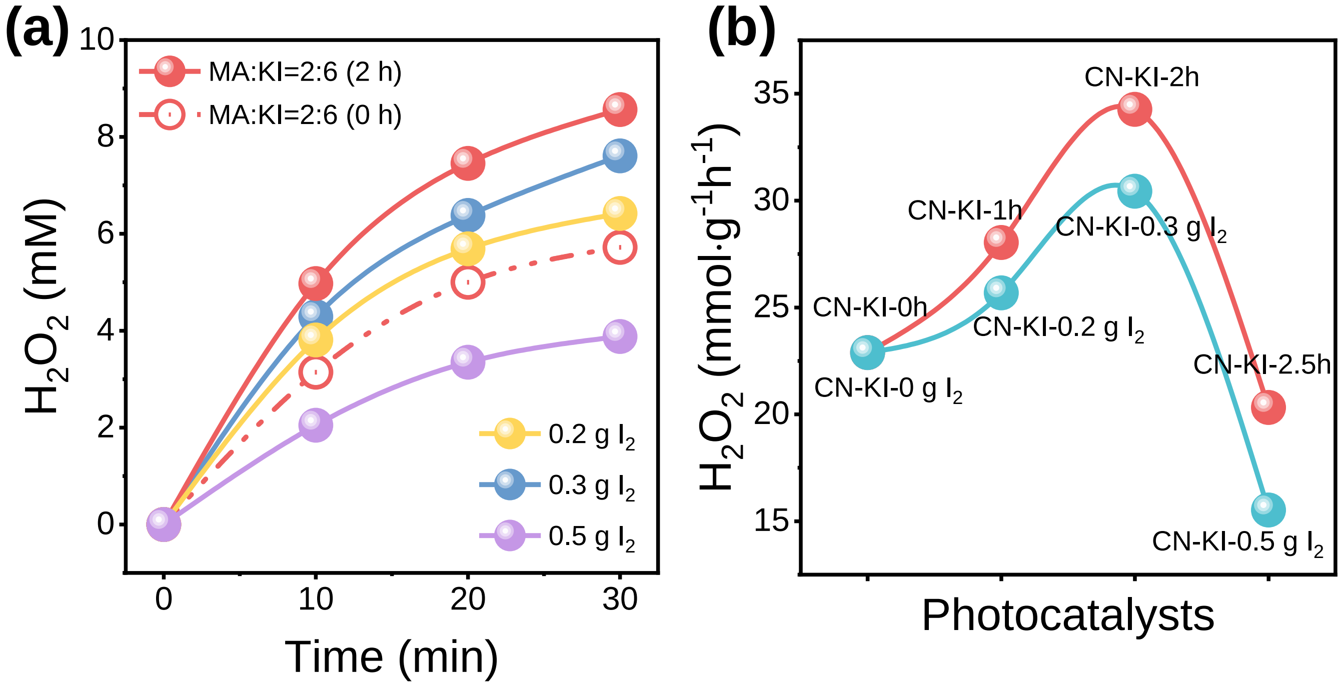

Supplementary Figure 55 | Photocatalytic H_2O_2 production activities (a) and corresponding evolution rate (b) of CN-KI-I_2.

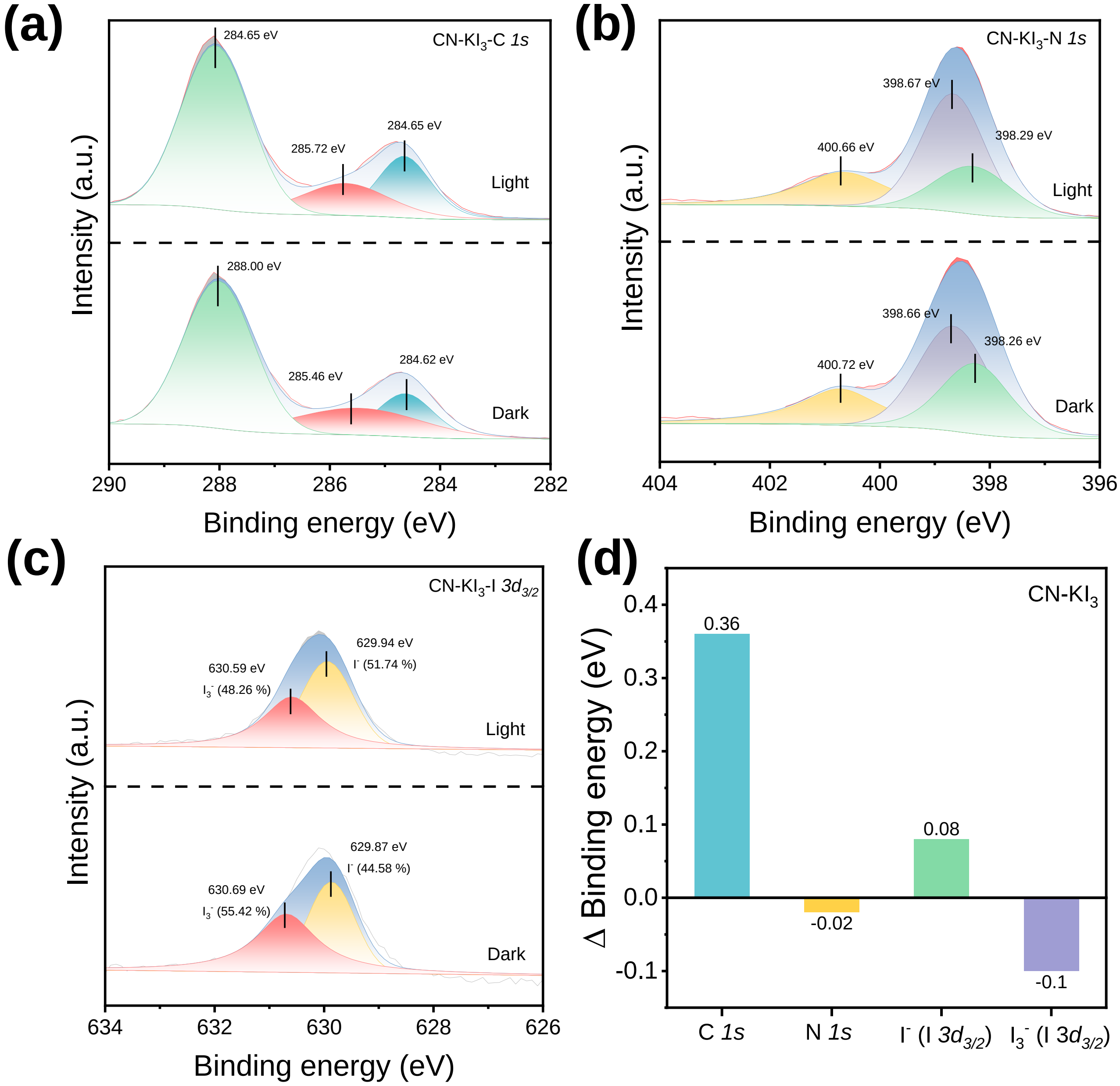

Supplementary Figure 56 | *Semi-in-situ* XPS spectra of CN-KI_3 under visible light and dark conditions. (a) C *1s*, (b) N *1s*, (c) I *3d*$_{3/2}$, (d) Δ Binding energy under visible light and dark conditions.

Using *in-situ* XPS technology, we investigated the electron transfer mechanism of the CN-KI_3 sample before and after illumination, as illustrated in Supplementary Figure **56**. Comparative analysis with CN revealed a significant increase in the binding energy of C *1s*, shifting from +0.14 eV to +0.36

eV. This shift suggests that the introduction of I_3^- could effectively improve the separation efficiency of photo-generated carriers in CN. More importantly, after illumination, the binding energy of I^- (I *$3d_{3/2}$*) in the CN-KI_3 sample increased by 0.08 eV, accompanied by a 7.16% increase in the proportion of I^- species. Conversely, the binding energy of I_3^- (I *$3d_{3/2}$*) decreased by 0.10 eV, accompanied by a corresponding 7.16% decrease in the proportion of I_3^- species. These intriguing findings provide compelling evidence for the mutual conversion between I^- and I_3^-, affirming their roles as redox mediators.

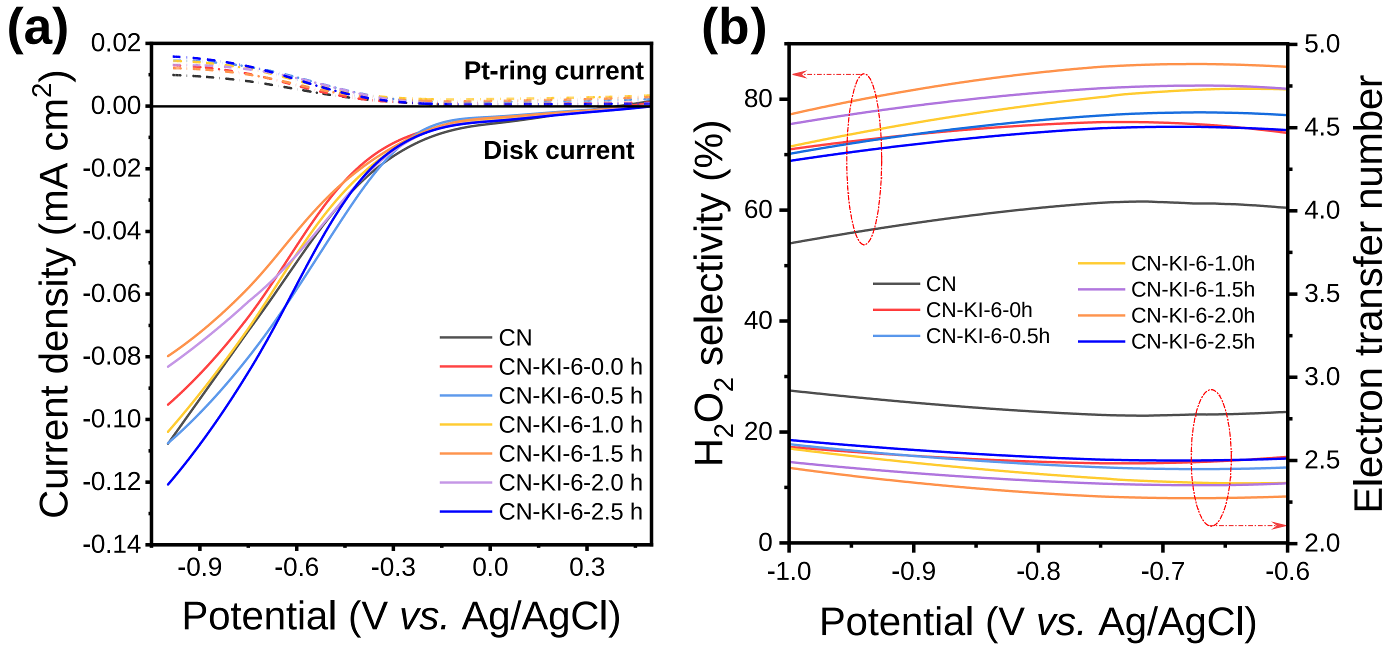

Supplementary Figure 57 | RRDE tests of samples with different irradiation times. (a) Polarization curves of the samples with different photocatalytic oxidation durations on a rotating disk electrode. (b) H_2O_2 selectivity and the average number of electron transfers in the corresponding ORR.

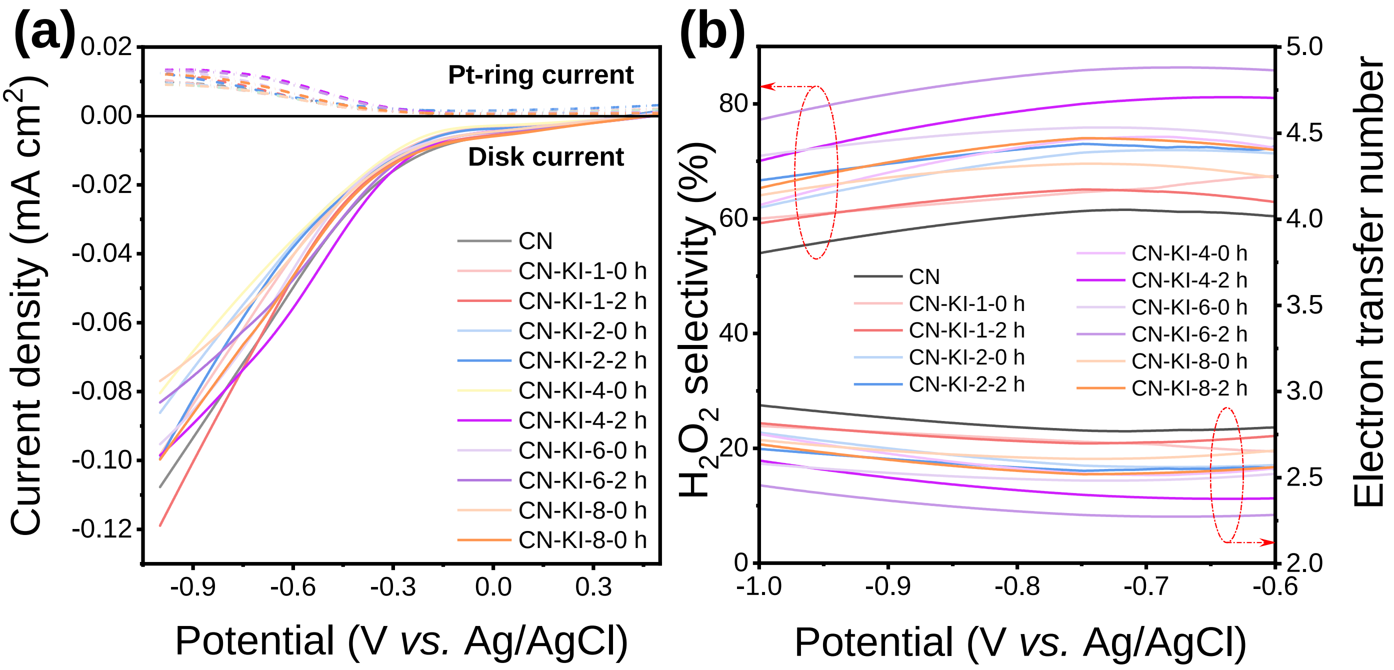

Supplementary Figure 58 | RRDE tests of samples with different KI ratios. (a) Polarization curves of the samples with different MA-KI ratios on a rotating disk electrode. (b) Selectivity of H_2O_2 and an average number of transferred electrons in the ORR.

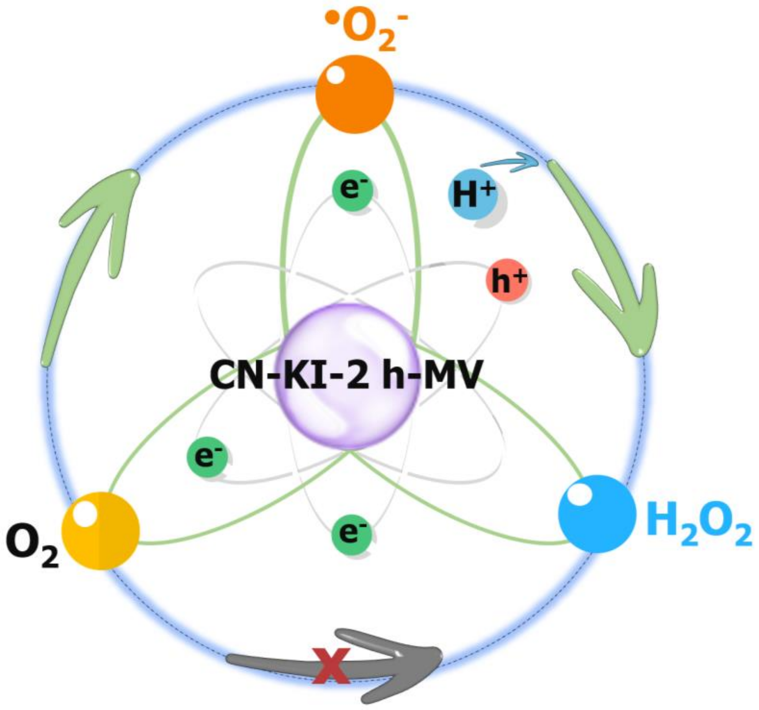

Supplementary Figure 59 | Schematic diagram of the electron transfer mechanism for H_2O_2 formation.

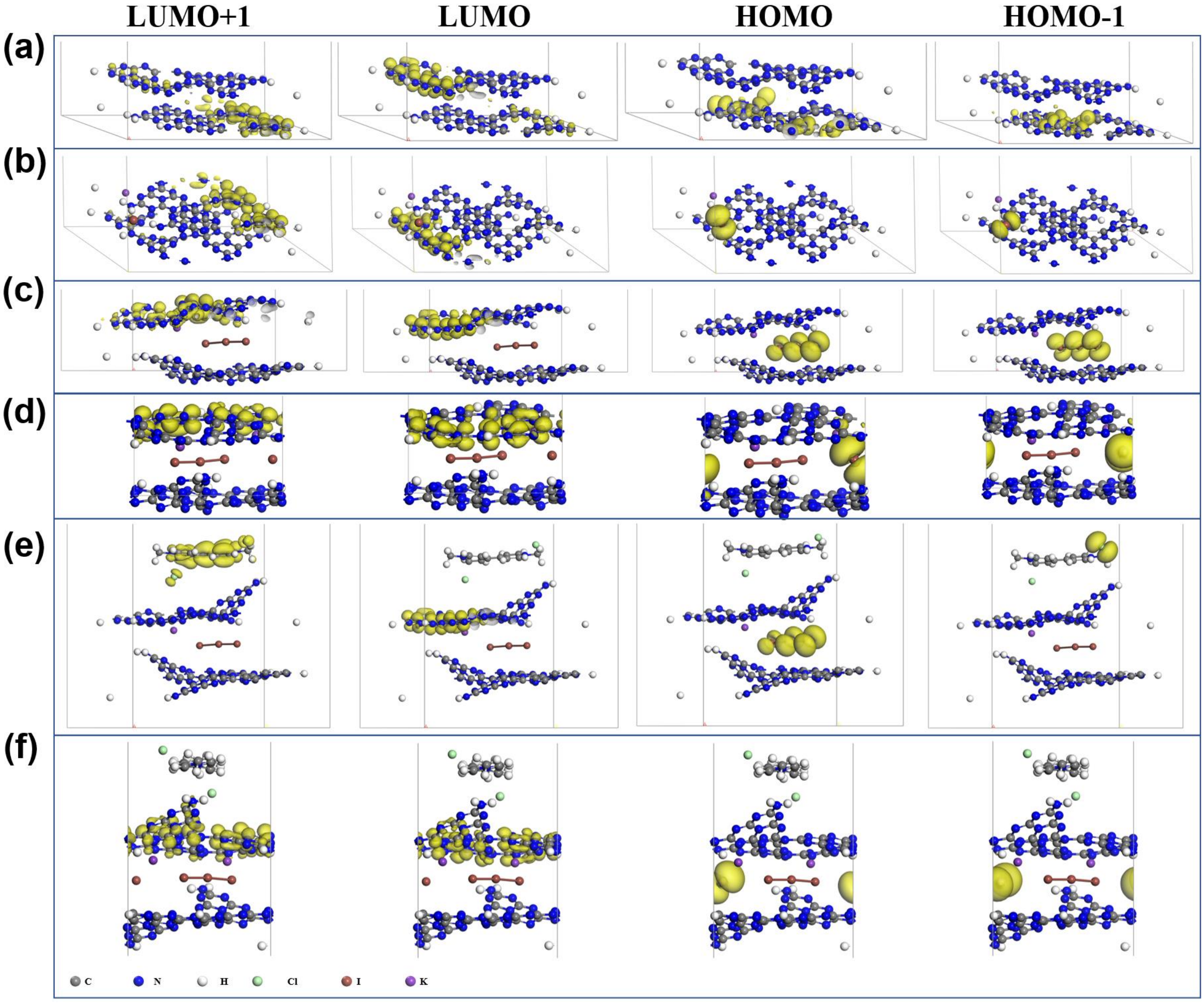

Supplementary Figure 60 | Charge density distribution of LUMO+1, LUMO, HOMO, and HOMO+1 in (a) CN, (b) CN-KI, (c) CN-KI_3, (d) CN-KI_3-KI, (e) CN-KI_3-MV, and (f) CN-KI_3-KI-MV.

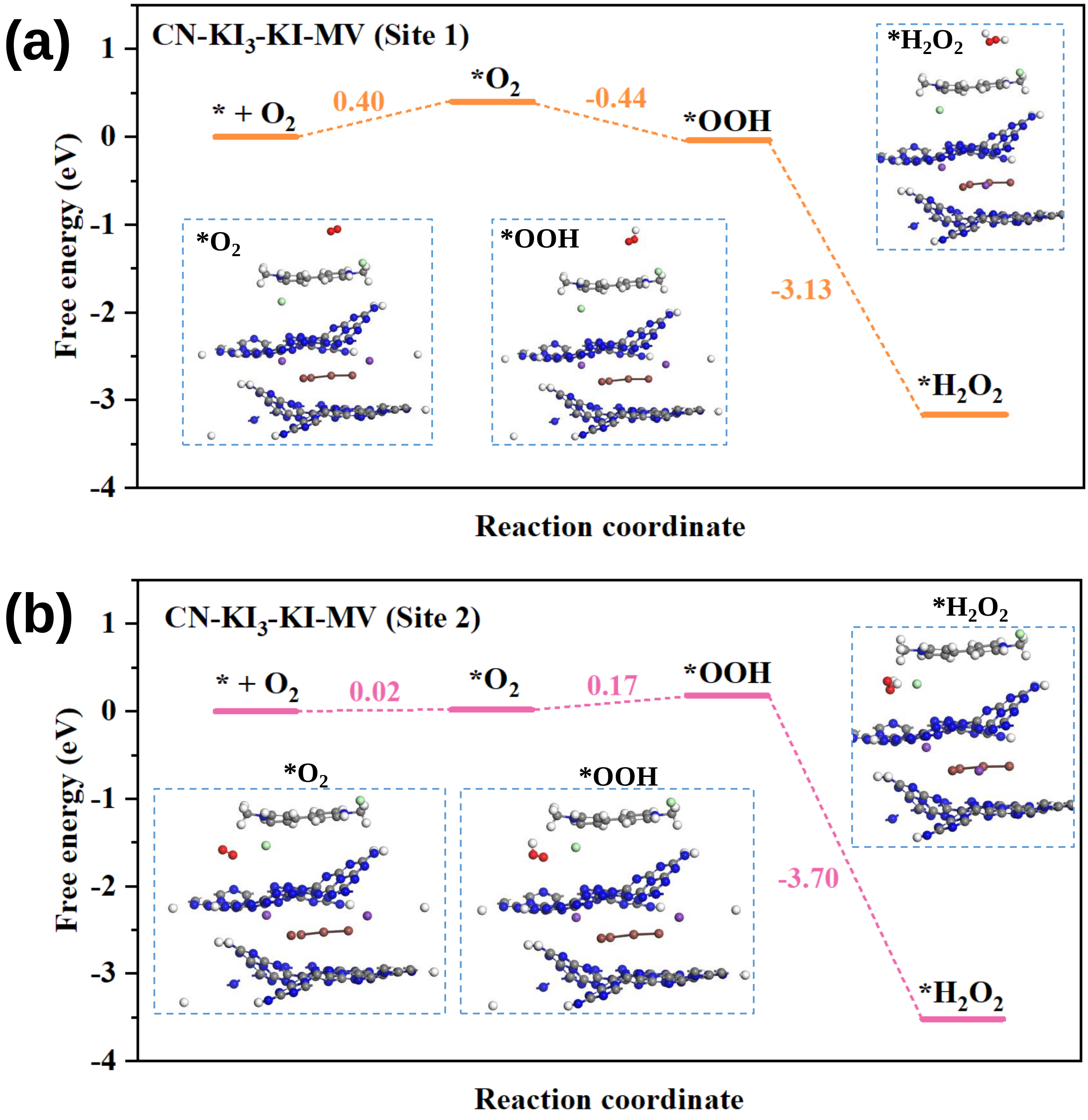

Supplementary Figure 61 | Energy diagram of the H_2O_2 evolution reaction at (a) Site 1 and (b) Site 2 on the surface of CN-KI_3-KI-MV. Gray represents carbon; blue represents nitrogen; white represents hydrogen; green represents chlorine; brown represents iodine; purple represents potassium; red represents oxygen.

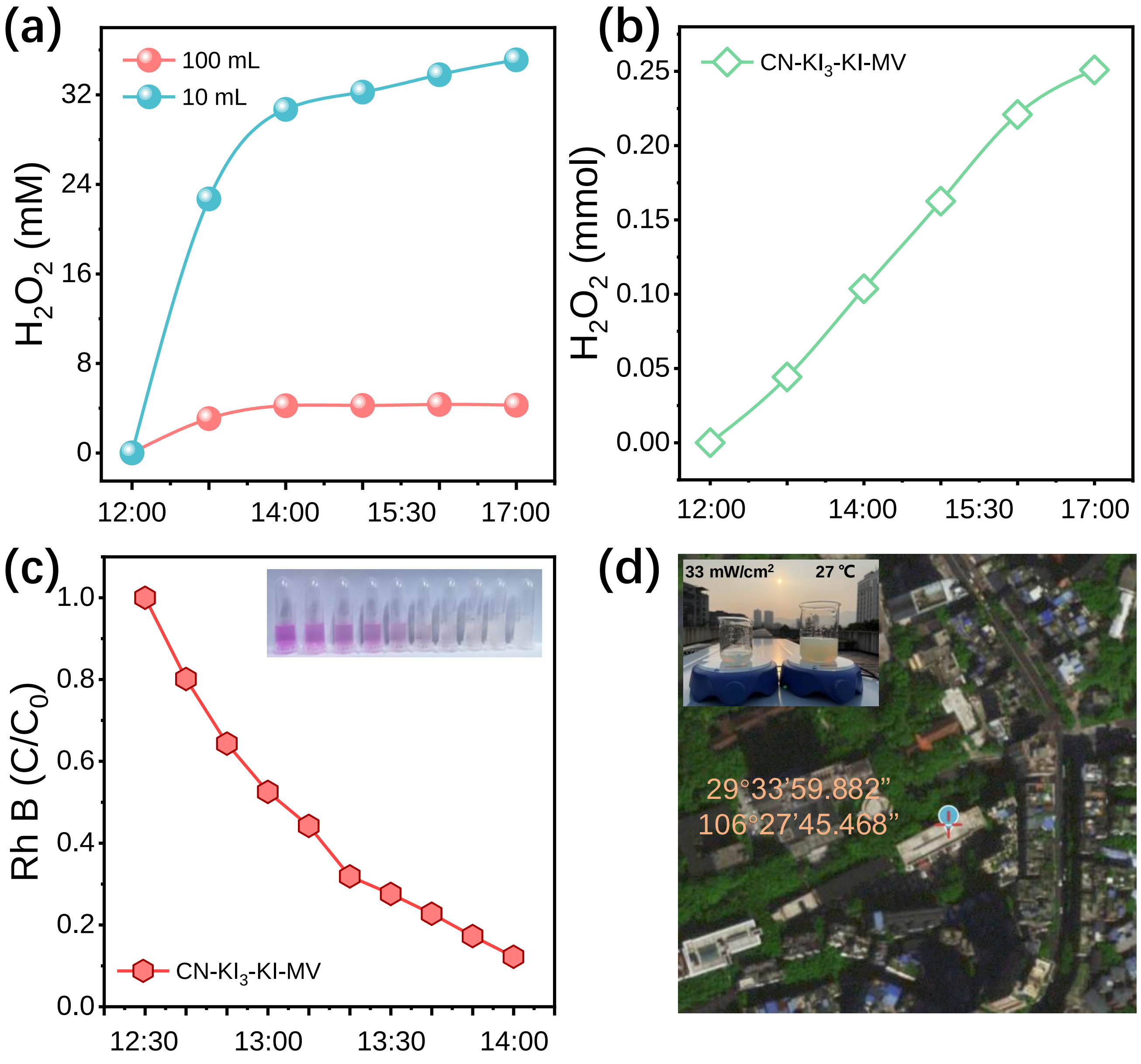

Supplementary Figure 62 | Outdoor photocatalytic activity assessment of the CN-KI$_3$-KI-MV system. (a) H_2O_2 production across varied working volumes (Samples were obtained outdoors in Chongqing City, China), (b) immobilized H_2O_2 production, and (c) RhB decolorization efficiency (Samples were collected outdoors in Chongqing City, China). Inset: Decolorization changes of RhB. (d) Detailed outdoor experimental location, including latitude and longitude. Inset: Physical diagrams of H_2O_2 production in different working volumes.

Owing to its dual-enhancement strategy, the constructed CN-KI_3-KI-MV also demonstrated remarkable catalytic prowess under direct exposure to natural sunlight, as illustrated in Supplementary Figure **62**. On the afternoon of October 18, 2023, in Chongqing City, China, we deliberately conducted our experiment under direct sunlight irradiation. The ambient temperature hovered around 27±3 °C, with an estimated light intensity of 33.0±4.5 mW cm^{-2}. The production of hydrogen peroxide spanned from 12:00 p.m. to 17:00 p.m. BST. Following this, the direct degradation of RhB was carried out between 12:00 p.m. and 14:00 p.m. BST. After a 5-hour reaction period, the H_2O_2 accumulation reached a significant 35.12 mM, with 4.27 mM recorded in an expanded working volume of 200 mL. In an immobilized production simulation, the accumulated H_2O_2 touched 0.25 mmol. Moreover, RhB (at a concentration of 20 mg L^{-1}) underwent nearly complete decolorization (96.71%) within 90 minutes in an oxidant-free setup. The efficacy of multifunctional applications under natural sunlight irradiation lays a solid groundwork for potential industrial applications.

From these preliminary investigations, we envision scaling up the system or constructing a large outdoor continuous flow unit for direct peroxygen production and concentration. Additionally, the system holds promise for purifying continuous outdoor organic wastewater. Impressively, this entire process demands minimal energy, aligning seamlessly with contemporary advancements and the broader trajectory of economic development.

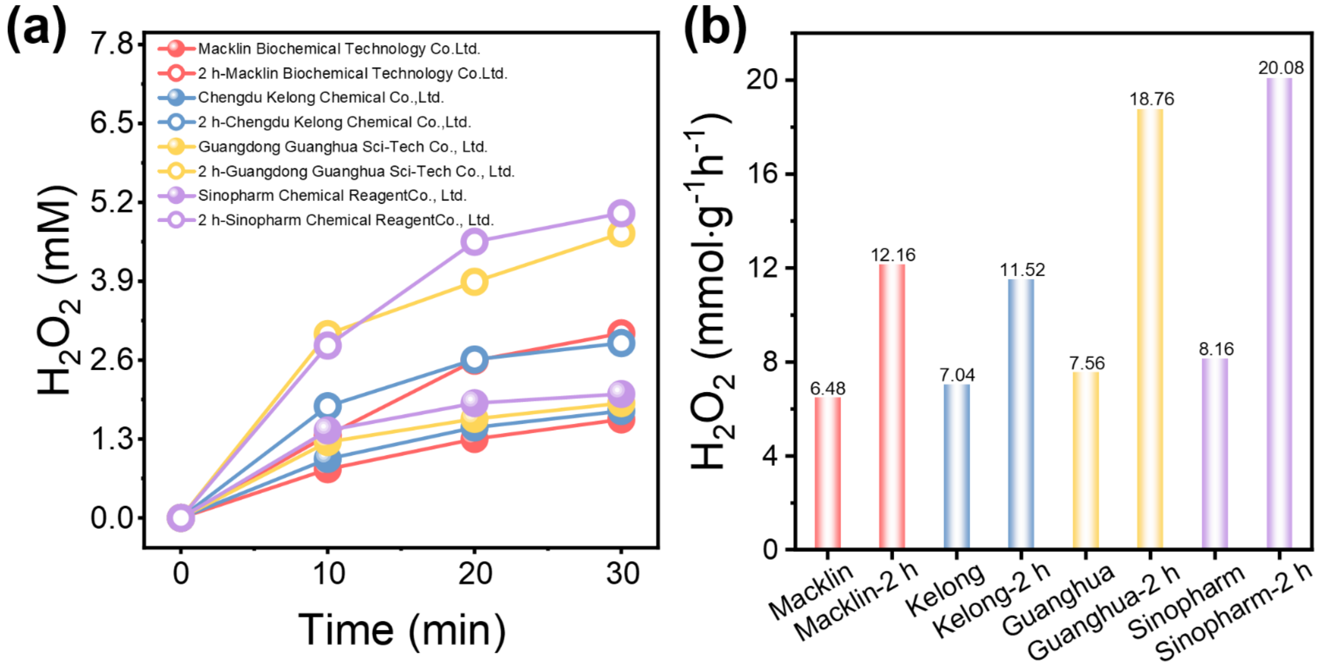

Supplementary Figure 63 | Photocatalytic H_2O_2 production (a) and corresponding evolution rates (b) for the KI samples obtained from different manufacturers.

Supplementary Table 1 | Optical properties of the prepared photocatalysts.

Sample	σ^* (m^2 kg^{-1})	κ^* (m^2 kg^{-1})	β^* (m^2 kg^{-1})
CN	700	643	1343
CN-KI	703	645	1348
CN-KI_3-KI	704	647	1351
CN-KI_3-KI-MV	711	651	1362

Supplementary Table 2 | The relationship between catalyst concentration and optical thickness.

Concentration (g L^{-1})	CN	CN-KI	CN-KI_3-KI	CN-KI_3-KI-MV
0.3	2.4174	2.4264	2.4318	2.4516
0.4	3.2232	3.2352	3.2424	3.2688
0.5	4.0290	4.0440	4.0530	4.0860
0.6	4.8348	4.8528	4.8636	4.9032
0.7	5.6406	5.6616	5.6742	5.7204

Supplementary Table 3 | The position attribution and proportion of characteristic peaks in the C*1s* spectrum.

Sample	N-C=N	C-NH_x/C≡N	C-C
CN	288.19 eV/58.03%	286.16 eV/6.78%	284.8 eV/35.19%
CN-KI	288.01eV/49.50%	286.39 eV/10.68%	284.8 eV/39.82%
CN-KI_3-KI	288.09 eV/31.71%	286.29 eV/19.96%	284.8 eV/48.33%
CN-KI_3-KI-MV	288.12 eV/42.93%	286.13 eV/24.0%	284.8 eV/33.07%

Supplementary Table 4 | The position attribution and proportion of characteristic peaks in the N *1s* spectrum.

Sample	C-N-H	N-C_3	C-N=C
CN	400.60 eV/10.93%	399.49 eV/16.42%	398.27 eV/74.45%
CN-KI	400.44 eV/7.66%	399.06 eV/6.13%	398.06 eV/86.21%
CN-KI_3-KI	400.52 eV/8.52%	399.59 eV/5.26%	397.96 eV/86.22%
CN-KI_3-KI-MV	400.50 eV/9.61%	399.09 eV/7.17%	398.02 eV/83.22%

Supplementary Table 5 | The position attribution and proportion of characteristic peaks in the I *3d* spectrum.

Sample	I^- $3d_{3/2}$	I_3^- $3d_{3/2}$
CN-KI	630.25 eV/100%	/
CN-KI_3-KI	630.21 eV/59.29%	631.86 eV/40.71%
CN-KI_3-KI-MV	629.85 eV/60.03%	630.60 eV/39.97%

Supplementary Table 6 | Comparison of fitted *fs*-TAS curve by biexponential function and triple exponential function.

Sample	Exp Dec 2				Exp Dec 3				
	τ_1 (ps)	τ_2 (ps)	R^2	Ave. τ (ps)	τ_1 (ps)	τ_2 (ps)	τ_3 (ps)	R^2	Ave. τ (ps)
**CN**	0.85 (51.06%)	375.81 (48.94%)	0.8341	374.93	0.85 (52.17%)	374.56 (23.91%)	376.28 (23.92%)	0.83361	374.50
**CN-KI**	0.99 (50%)	304.71 (50%)	0.86433	303.17	7.78 (41.79%)	108.33 (34.33%)	427.65 (23.88%)	0.86314	341.14
**CN-KI_3-KI**	7.87 (60.94%)	274.53 (39.06%)	0.85062	263.12	6.62 (53.13%)	89.59 (28.13%)	386.24 (18.74%)	0.84959	299.12
**CN-KI_3-KI-MV**	6.13 (61.91%)	241.76 (38.09%)	0.83345	232.44	6.46 (67.55%)	159.25 (17.88%)	323.64 (14.57%)	0.79906	247.82

To gain further insights into the decay process of photo-generated carriers within the catalyst, we conducted a triexponential function fitting analysis on the *fs*-TAS results, as detailed in Supplementary Table **6**. The constants τ_1, τ_2, and τ_3 correspond to the capture of photo-generated electrons in shallow traps, hole capture, and subsequent charge recombination processes, respectively. Comparative analysis revealed notable differences between the samples and CN. Specifically, the slower decay of shallow electrons, prolonged charge recombination time, and accelerated hole capture process in the modified catalysts suggest that the employed modification strategy effectively mitigated the direct recombination of photo-generated carriers. This, in turn, resulted in a reduction in the average lifetime of photo-generated carriers, indicative of enhanced carrier separation efficiency (CN: 374.50 ps, CN-KI: 341.14, CN-KI_3-KI: 299.12, CN-KI_3-KI-MV: 247.82 ps). Consistency with the results obtained from

biexponential function fitting underscores the efficacy of our proposed design strategy in augmenting the photocatalytic activity of CN-based photocatalysts.

Supplementary Table 7 | Collected data of photocatalytic production H_2O_2 based on $g\text{-}C_3N_4$ materials for comparison.

Photocatalyst	Reaction conditions	H_2O_2 (mmol g^{-1} h^{-1})	Ref.
$g\text{-}C_3N_4$-CNTs	Catalyst 1 g/L, Formic acid 10%, O_2, λ>420 nm	0.13	1
CM-$g\text{-}C_3N_4$	Catalyst 0.1 g/L, TEOA 20%, O_2, λ>420 nm	0.14	2
Cv-$g\text{-}C_3N_4$	Catalyst 1 g/L, EtOH 5%, O_2, λ>420 nm	0.16	3
Au-$g\text{-}C_3N_4$	Catalyst 4 g/L, EtOH 10%, O_2, λ>420 nm	0.17	4
GCN	Catalyst 4 g/L, EtOH 10%, O_2, λ>420 nm	0.18	5
K/P/O-CN	Catalyst 0.5 g/L, EtOH 10%, O_2, λ>420 nm	0.49	6
K_2HPO_4/CN	Catalyst 1 g/L, EtOH 10%, O_2, λ>420 nm	0.50	7
S-pCN/WO_2	Catalyst 0.5 g/L, TEOA 10%, O_2, λ>420 nm	0.75	8
KOH-CN	Catalyst 1 g/L, Methanol 10%, O_2, λ>420 nm	1.00	9
Ni_2P/CDs	Catalyst 0.5 g/L, / O_2, λ>420 nm	1.1	10
O-CN	Catalyst 0.5 g/L, isopropanol 10%, O_2, λ>420 nm	1.20	11
K^+/Na^+-CN	Catalyst 1 g/L, EtOH 0.789 g/L O_2, λ>400 nm	1.28	12
Cu-C_3N_4	Catalyst 1 g/L, EtOH 0.789 g/L	1.30	13

	O_2, λ>400 nm		
Pt-Na^+-g-C_3N_4	Catalyst 1 g/L, / O_2, λ>420 nm	1.5	14
O-C_3N_4-Ag^{2+}	Catalyst 0.4 g/L, isopropanol 10%, O_2, λ>350 nm	1.99	15
Ti_3C_2-pCN	Catalyst 1 g/L, isopropanol 10%, O_2, λ>420 nm	2.63	16
S/K-CN	Catalyst 0.5 g/L, ethanol 10%, O_2, λ>420 nm	2.74	17
Nv-C_3N_4	Catalyst 1 g/L, 1.0 EtOH 0.789 g/L, O_2, λ>400 nm	4.4	18
K-CN	Catalyst 1 g/L, isopropanol 0.5%, O_2, λ>420 nm	5.50	19
Na-C_3N_4	Catalyst 0.5 g/L, isopropanol 10%, O_2, λ>420 nm	6.53	20
C≡N-Na-CN	Catalyst 1 g/L, EtOH 10%, O_2, λ>420 nm	7.01	21
K/S/O-CN	Catalyst 0.5 g/L, ethanol 10%, O_2, λ>420 nm	8.92	22
K/Na-CN	Catalyst 0.5 g/L, isopropanol 10%, O_2, λ>420 nm	10.20	23
C-C_3N_4	Catalyst 1 g/L, isopropanol 5%, O_2, λ>420 nm	10.59	24
N/O-CN	Catalyst 0.5 g/L, isopropanol 10%, O_2, λ>420 nm	11.14	25
KCl/KI-CN	Catalyst 0.2 g/L, isopropanol 10%, air, λ>400 nm	13.10	26
O/K-CN	Catalyst 0.5 g/L, isopropanol 10%,	15.47	27

	O_2, λ>420 nm		
Al-C_3N_4	Catalyst 0.125 g/L, isopropanol 20%, O_2, λ>420 nm	27.50	28
CN-KI_3-KI-MV	Catalyst 0.5 g/L, isopropanol 10%, air, λ>420 nm	46.40	**This work**

Supplementary Table 8 | Collected the AQY indexes of photocatalytic production H_2O_2 based on g-C_3N_4 materials for comparison.

Catalyst	Reaction solution and catalytic	Light Source	AQY	Ref.
Ni_{SAPs}-PuCN	Pure water (1 g/L)	λ≥420 nm	14.31% (400 nm)	29
Sb-SACS	Pure water (2 g/L)	λ≥420 nm	18.3% (400 nm)	30
Al-C_3N_4	20% IPA (0.125 g/L)	λ>420 nm	6.2% (400 nm)	28
OCN	10% IPA (0.5 g/L)	λ≥420 nm	17.2% (400 nm)	11
CN-KI_3-KI-MV	10% IPA (0.5 g/L)	λ>420 nm	27.56% (400 nm)	**This work**

Supplementary Table 9 | Ecological Structure-Activity Relationship system predicts the acute and chronic toxicity[a] of Sulfonamide (SA) and its degradation products.

	Possible products	Acute toxicity (mg L^{-1})			Chronic toxicity (mg·L^{-1}): ChV		
		fish (LC_{50})	daphnid (LC_{50})	green algae (EC_{50})	fish	daphnid	green algae
SA	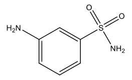	854	8.04	32.6	24.5	0.07	25.5
1	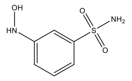	627	246	405	841	733	623
2	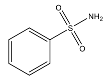	376	183	733	307	118	137
3	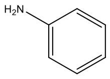	40.3	1.67	5.1	0.59	0.019	2.04
4	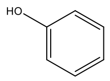	212	115	71.1	19.6	9.89	16.8
5	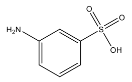	200	306	526	465	1.67	257
6	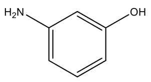	165	3.19	11.3	3.41	0.03	6.36
7	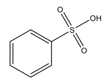	1904	1830	1530	1260	966	816

[a] Toxicity was classified based on the Globally Harmonized System of Classification and Labeling of Chemicals. Very toxic: $LC_{50}/EC_{50}/ChV \leq 1$ mg L^{-1}, Toxic: 1 mg $L^{-1} < LC_{50}/EC_{50}/ChV \leq 10$ mg L^{-1}, Harmful: 10 mg L^{-1} < $LC_{50}/EC_{50}/ChV \leq 100$ mg L^{-1}, Not harmful: $LC_{50}/EC_{50}/ChV > 100$ mg L^{-1}.

Supplementary Table 10 | Ecological Structure-Activity Relationship system predicts the acute and chronic toxicity[a] of Naproxen (NPX) and its degradation products.

	Possible products	Acute toxicity (mg L^{-1})			Chronic toxicity (mg·L^{-1}): ChV		
		fish (LC_{50})	daphnid (LC_{50})	green algae (EC_{50})	fish	daphnid	green algae
NPX	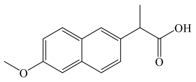	193	122	138	21.3	15.7	45.3
1	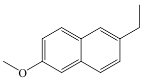	1.35	0.945	1.69	0.169	0.166	0.707
2	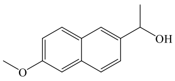	35.2	21.4	21.3	3.74	2.54	6.5
3	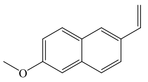	1.77	1.22	2.07	0.218	0.208	0.845
4	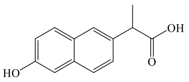	579	346	317	60.1	38.8	92.7
5	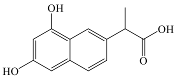	680	859	731	165	95	194
6	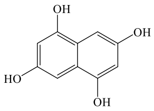	747	396	221	67.3	31.8	49.7
7	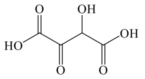	957	434	668	344	287	1063

[a] Toxicity was classified based on the Globally Harmonized System of Classification and Labeling of Chemicals. Very toxic: $LC_{50}/EC_{50}/ChV \leq 1$ mg L^{-1}, Toxic: 1 mg $L^{-1} < LC_{50}/EC_{50}/ChV \leq 10$ mg L^{-1}, Harmful: 10 mg $L^{-1} < LC_{50}/EC_{50}/ChV \leq 100$ mg L^{-1}, Not harmful: $LC_{50}/EC_{50}/ChV > 100$ mg L^{-1}.

Supplementary Table 11 | The position attribution of different characteristic peaks in the C *1s* spectrum under light and dark conditions.

Sample	Dark			Light		
	N-C=N	C-NH_x/C≡N	C-C	N-C=N	C-NH_x/C≡N	C-C
CN	287.81 eV	285.93 eV	284.45 eV	287.83 eV	286.06 eV	284.44 eV
CN-KI	287.61 eV	286.01 eV	284.40 eV	287.74 eV	286.02 eV	284.56 eV
CN-KI_3	288.00 eV	285.46 eV	284.62 eV	288.07 eV	285.72 eV	284.65 eV
CN-KI_3-KI_3	287.45 eV	285.70 eV	284.40 eV	287.63 eV	285.81 eV	284.39 eV
CN-KI_3-KI-MV	287.60 eV	285.71 eV	284.45 eV	287.70 eV	285.84 eV	284.51 eV

Supplementary Table 12 | The position attribution of different characteristic peaks in the N *1s* spectrum under light and dark conditions.

Sample	Dark			Light		
	C-N-H	N-C_3	C-N=C	C-N-H	N-C_3	C-N=C
CN	400.58 eV	398.85 eV	398.19 eV	400.49 eV	398.67 eV	398.18 eV
CN-KI	400.43 eV	398.25 eV	397.95 eV	400.30 eV	398.31 eV	397.77 eV
CN-KI_3	400.72 eV	398.66 eV	398.26 eV	400.66 eV	398.67 eV	398.29 eV
CN-KI_3-KI	400.47 eV	398.38 eV	397.84 eV	400.25 eV	398.13 eV	397.88 eV
CN-KI_3-KI-MV	400.47 eV	398.55 eV	397.94 eV	400.35 eV	398.29 eV	397.84 eV

Supplementary Table 13 | The position attribution of different characteristic peaks in the I $3d_{3/2}$ spectrum under light and dark conditions.

Sample	Dark (I $3d_{3/2}$)		Light (I $3d_{3/2}$)	
	I^-	I_3^-	I^-	I_3^-
CN-KI	629.68 eV	/	629.74 eV	630.66 eV
CN-KI_3	629.87 eV	630.69 eV	629.94 eV	630.59 eV
CN-KI_3-KI	629.76 eV	630.56 eV	629.85 eV	630.50 eV
CN-KI_3-KI-MV	629.27 eV	630.45 eV	629.41 eV	630.32 eV

Supplementary Table 14 | Changes in binding energies of C *1s*, N *1s*, and I $3d_{3/2}$ spectrum after illumination.

Sample	Δ Binding energy (eV) (Dark→Visible light)			
	C *1s*	N *1s*	I^- (I $3d_{3/2}$)	I_3^- (I $3d_{3/2}$)
CN	+0.14 eV	-0.28 eV	/	/
CN-KI	+0.24 eV	-0.37 eV	+0.06 eV	/
CN-KI_3	+0.36 eV	-0.02 eV	+0.08 eV	-0.10 eV
CN-KI_3-KI	+0.28 eV	-0.43 eV	+0.09 eV	-0.06 eV
CN-KI_3-KI-MV	+0.33 eV	-0.48 eV	+0.14 eV	-0.13 eV

References:

1. Zhao, S., Guo, T., Li, X., Xu, T., Yang, B. & Zhao, X. Carbon nanotubes covalent combined with graphitic carbon nitride for photocatalytic hydrogen peroxide production under visible light. *Appl. Catal. B-Environ*. **224**, 725-732 (2018).
2. Mahvelati-Shamsabadi, T., Fattahimoghaddam, H., Lee, B., Bae, S. & Ryu, J. Synthesis of hexagonal rosettes of g-C_3N_4 with boosted charge transfer for the enhanced visible-light photocatalytic hydrogen evolution and hydrogen peroxide production. *J. Colloid. Interf. Sci*. **597**, 345-360 (2021).
3. Lei, J., Chen, B., Lv, W., Zhou, L., Wang, L., Liu, Y. & Zhang, J. Robust Photocatalytic H_2O_2 Production over Inverse Opal g-C_3N_4 with Carbon Vacancy under Visible Light. *ACS Sustain. Chem. Eng*. **7**, 16467–16473 (2019).
4. Zuo, G. et al.Finely dispersed Au nanoparticles on graphitic carbon nitride as highly active photocatalyst for hydrogen peroxide production. *Catal. Commun*. **123**, 69 (2019).
5. Fattahimoghaddam, H., Mahvelati-Shamsabadi, T. & Lee, B. Enhancement In Photocatalytic H_2O_2 Production Over g-C_3n_4 Nanostructures: A Collaborative Approach Of Nitrogen Deficiency And Supramolecular Precursors. *ACS Sustain. Chem. Eng*. **9**, 4520-4530 (2021).
6. Moon, G., Fujitsuka, M., Kim, S., Majima, T., Wang, X. & Choi, W. Eco-Friendly Photochemical Production of H_2O_2 through O_2 Reduction over Carbon Nitride Frameworks Incorporated with Multiple Heteroelements. *ACS Catal*. **7**, 2886-2895 (2017).
7. Tian, J., Wu, T., Wang, D., Pei, Y., Qiao, M. & Zong, B. One-pot synthesis of potassium and phosphorus-doped carbon nitride catalyst derived from urea for highly efficient visible light-driven hydrogen peroxide production. *Catal. Today*. **330**, 171-178 (2019).
8. Li, X. et al. Enhanced photocatalytic degradation and H_2/H_2O_2 production performance of S-pCN/$WO_{2.72}$ S-scheme heterojunction with appropriate surface oxygen vacancies. *Nano. Energ*. **81**, 105671 (2021).

9. Wang, Y., Di Meng & Zhao, X. Visible-light-driven H_2O_2 production from O_2 reduction with nitrogen vacancy-rich and porous graphitic carbon nitride. *Appl. Catal. B-Environ.* **273**, 119064 (2020).
10. Liu, Y., Zhao, Y., Wu, Q., Wang, X. & Kang, Z. Charge storage of carbon dot enhances photo-production of H_2 and H_2O_2 over Ni_2P/carbon dot catalyst under normal pressure. *Chem. Eng. J.* **409**, 128184 (2020).
11. Wei, Z., Liu, M., Zhang, Z., Yao, W., Tan, H. & Zhu, Y. Efficient visible-light-driven selective oxygen reduction to hydrogen peroxide by oxygen-enriched graphitic carbon nitride polymers. *Energ. Environ. Sci.* **11**, 2581-2589 (2018).
12. Qu, X., Hu, S., Bai, J., Li, P., Lu, G. & Kang, X. Synthesis of band gap-tunable alkali metal modified graphitic carbon nitride with outstanding photocatalytic H202 production ability via molten salt method. *J. Mater. Sci. Technol.* **34**, 1932-1938 (2018).
13. Hu, S. et al. Photocatalytic oxygen reduction to hydrogen peroxide over copper doped graphitic carbon nitride hollow microsphere: The effect of Cu(I)-N active sites. *Chem. Eng. J.* **334**, 410-418 (2018).
14. Liang, F., Sun, X., Hu, S., Ma, H., Wang, F. & Wu, G. Photocatalytic water splitting to simultaneously produce H_2 and H_2O_2 by two-electron reduction process over Pt loaded Na^+ introduced g-C_3N_4 catalyst. *Diam. Relat. Mater.* **108**, 107971 (2020).
15. Xiong, C., Jiang, S., Song, S., Wu, X., Li, J. & Le, Z. Solid-Solution-Like O-C_3N_4/Ag_2SO_4 Nanocomposite as a Direct Z-Scheme Photocatalytic System for Photosynthesis of Active Oxygen Species. *ACS Sustain. Chem. Eng.* **6**, 10905-10913 (2018).
16. Yang, Y. et al. Ti_3C_2 Mxene/porous g-C_3N_4 interfacial Schottky junction for boosting spatial charge separation in photocatalytic H_2O_2 production. *Appl. Catal. B-Environ.* **258**, 117956 (2019).
17. Zhang, P. et al. Heteroatom dopants-promoted two-electron O_2 reduction for photocatalytic production of H_2O_2 on polymeric carbon nitride. *Angew. Chem. Int.*

Edit. **59**, 16209 (2020).

18. Li, X. et al. Preparation of N-vacancy-doped g-C_3N_4 with outstanding photocatalytic H_2O_2 production ability by dielectric barrier discharge plasma treatment. *Chinese. J. Catal*. **39**, 1090-1098 (2018).
19. Zhang, J. et al. Modulation of Lewis acidic-basic sites for efficient photocatalytic H_2O_2 production over potassium intercalated tri-s-triazine materials. *Appl. Catal. B-Environ*. **277**, 119225 (2020).
20. Che, H., Gao, X., Chen, J., Hou, J., Ao, Y. & Wang, P. Iodide-Induced Fragmentation of Polymerized Hydrophilic Carbon Nitride for High-Performance Quasi-Homogeneous Photocatalytic H_2O_2 Production. *Angew Chem. Int. Edit*. **60**, 25546 (2021).
21. Chen, L., Chen, C., Yang, Z., Li, S., Chu, C. & Chen, B. Simultaneously Tuning Band Structure and Oxygen Reduction Pathway toward High-Efficient Photocatalytic Hydrogen Peroxide Production Using Cyano-Rich Graphitic Carbon Nitride. *Adv. Funct. Mater*. **31**, 2105731 (2021).
22. Li, S. et al. Multiple heteroatom-doped urea and thiourea-derived polymeric carbon nitride for high-performance visible light-driven photocatalytic O_2 reduction to H_2O_2. *Appl. Catal. B-Environ*. **335**, 122879 (2023).
23. Wu, S., Yu, H., Chen, S. & Quan, X. Enhanced Photocatalytic H_2O_2 Production Over Carbon Nitride By Doping And Defect Engineering. *ACS Catal*. **10**, 14380–14389 (2020).
24. Wang, R. et al. Energy-level dependent H_2O_2 production on metal-free, carbon-content tunable carbon nitride photocatalysts. *J. Energy. Chem*. **27**, 343-350 (2018).
25. Zhao, H. et al. Rational design of carbon nitride for remarkable photocatalytic H_2O_2 production. *Chem. Catal*. **2**, 1720-1733 (2022).
26. Liu, L., Chen, F., Wu, J., Chen, J. & Yu, H. Synergy of crystallinity modulation and intercalation engineering in carbon nitride for boosted H_2O_2 photosynthesis. *P. Natl. Acad. Sci. Usa*. **120**, 156912 (2023).

27. Liu, W. et al. Unraveling the Mechanism on Ultrahigh Efficiency Photocatalytic H_2O_2 Generation for Dual-Heteroatom Incorporated Polymeric Carbon Nitride. *Adv. Funct. Mater*. **32**, 2205119 (2022).
28. Zhuang, C. et al. Monodispersed aluminum in carbon nitride creates highly efficient nitrogen active sites for ultra-high hydrogen peroxide photoproduction. *Nano Energ*. **108**, 108225 (2023).
29. Zhang, X. et al. Developing Ni single-atom sites in carbon nitride for efficient photocatalytic H_2O_2 production. *Nat. Commun.* **14**, 7115 (2023).
30. Teng, Z. et al. Atomically dispersed antimony on carbon nitride for the artificial photosynthesis of hydrogen peroxide. *Nat. Catal.* **4**, 374-384 (2021).
